# Framework for Characterizing Longitudinal Antibody Response in Children After *Plasmodium falciparum* Infection

**DOI:** 10.3389/fimmu.2021.617951

**Published:** 2021-03-02

**Authors:** Eric Rogier, Doug Nace, Pedro R. Dimbu, Brian Wakeman, Jan Pohl, James G. Beeson, Chris Drakeley, Kevin Tetteh, Mateusz Plucinski

**Affiliations:** ^1^Malaria Branch, Division of Parasitic Diseases and Malaria, Centers for Disease Control and Prevention, Atlanta, GA, United States; ^2^National Malaria Control Program, Luanda, Angola; ^3^Division of Scientific Resources, Centers for Disease Control and Prevention, Atlanta, GA, United States; ^4^Burnet Institute, Melbourne, VIC, Australia; ^5^Central Clinical School, Monash University, Melbourne, VIC, Australia; ^6^Department of Medicine, University of Melbourne, Melbourne, VIC, Australia; ^7^London School of Hygiene and Tropical Medicine, London, United Kingdom; ^8^U.S. President's Malaria Initiative, Centers for Disease Control and Prevention, Atlanta, GA, United States

**Keywords:** *Plasmodium faciparum*, malaria, antibody dynamics, antigens, antibody classes

## Abstract

Human *Plasmodium* infection produces a robust adaptive immune response. Time courses for 104 children followed for 42 days after initiation of *Plasmodium falciparum* chemotherapy were assayed for antibody levels to the five isotypes of human immunoglobulins (Ig) and 4 subclasses of IgG for 32 *P. falciparum* antigens encompassing all 4 parasite stages of human infection. IgD and IgE against these antigens were undetectable at 1:100 serum concentration, but other Ig isotypes and IgG subclasses were consistently observed against all antigens. Five quantitative parameters were developed to directly compare Ig response among isotypes and antigens: C_max_, maximum antibody level; Δ_C_, difference between C_max_ and the antibody level at Day 0; t_max_, time in days to reach C_max_; t_1/2_, Ig signal half-life in days; t_neg_, estimated number of days until complete loss of Ig signal. Classical Ig patterns for a bloodborne pathogen were seen with IgM showing early t_max_ and IgG production highest among Ig isotypes. However, some unexpected trends were observed such as IgA showing a biphasic pattern for many antigens. Variability among these dynamics of Ig acquisition and loss was noted for different *P. falciparum* antigens and able to be compared both quantitatively and statistically. This parametrization methodology allows direct comparison of Ig isotypes produced against various *Plasmodium* antigens following malaria infection, and the same methodology could be applied to other longitudinal serologic studies from *P. falciparum* or different pathogens. Specifically for *P. falciparum* seroepidemiological studies, reliable and quantitative estimates regarding the IgG dynamics in human populations can better optimize modeling efforts for serological outputs.

## Introduction

The vertebrate hosts of malaria have been required to endure malaria infection for tens of millions of years, and fossil evidence for *Plasmodium* and their predecessors far precedes the rise of the genus *Homo* (1, 2). The introduction of standardized antimalarial medications and therapeutics has only occurred very recently in human history (3), and intense evolutionary pressure has been placed on the human genome to alter traits of red blood cells as well as ensure a vigorous, yet measured, immune response against the parasite is elicited (4). Numerous mechanisms have been elucidated that combat *Plasmodium* infection within both the innate and adaptive arms of the immune system (5–9). Understanding these mechanism has been crucial in the development of the next generation of malaria vaccines (and adjuvants), and the potential for novel therapeutics (3, 10–12). Outside of understanding antimalarial immune responses for prophylactic or therapeutic purposes, these immune functions also result in informative biomarkers for exposure estimates and epidemiological investigations (13–15).

Antibody-mediated immune responses to malaria have been studied extensively in mammalian models and humans, and association between malaria infection and production of antibodies has been investigated since the mid-twentieth century (16–18). More recently, purified malaria antigens in the form of peptides and recombinant proteins have been characterized for their specific abilities to induce B cell responses and antibody production in exposed populations (14, 19–22). The pre-dominance of antigens in laboratory use today are produced during blood stage *Plasmodium* infection and would be expected to be at abundant levels in the host blood during the merozoite multiplication cycles. Outside of these erythrocyte-stage antigens, antigens eliciting antibody and other adaptive responses have also been identified for the other human *Plasmodium falciparum* infection stages: sporozoite (23, 24), liver (hepatocyte) (25), and sexual (gametocyte) (26). Class-switched populations of both effector and memory B cells develop after malaria exposure in humans, and more recently, memory B cells activated by *Plasmodium* antigens have been further subdivided into “classical” and “atypical” (27, 28). While classical B cell populations would more likely be developed over time by repeated exposures and have undergone affinity maturation (29), atypical populations of these antibody-secreting cells have been shown by multiple studies to be potentially shorter-lived, and it has been proposed these should not be considered memory B cells at all (30, 31).

Here we present data showing the dynamics of all classes of human antibody responses to a panel of 32 *P. falciparum* antigens in convalescent children who had been successfully treated for *P. falciparum* infection. Selected antigens covered all stages of *P. falciparum* infection in the human host, and additional antigens representing non-falciparum *Plasmodia* and other non-malaria pathogens were included to assess cross-binding or non-specific antibody/antigen interaction. Children were sampled weekly until 42 days after treatment, with all five immunoglobulin isotypes and the four subclasses of IgG assayed for in blood samples for specific antigen binding. Parameterization and direct comparison of Ig levels may allow a more systematic examination of antibody responses post-infection for and provide valuable frameworks for vaccine evaluation and seroepidemiological studies. Quantitatively contrasting the defined parameters for a study population among Ig isotypes and among antigens can assist in elucidating the true differences in humoral responses to infection or vaccination.

## Materials and Methods

### Study Design

We analyzed blood samples from children followed longitudinally after successful antimalarial treatment. We determined the presence of and quantified the concentration of antibodies to a panel of 37 antigens separately for IgA, IgD, IgE, IgG_1_, IgG_2_, IgG_3_, IgG_4_, and IgM.

### Sample Collection

Samples from therapeutic efficacy trials from 2017 in three sentinel sites in Angola (32) were analyzed. Participants were children 6 months to 11 years of age with uncomplicated *P. falciparum* single-species infection who were treated with artemisinin-based combination therapy, with follow-up blood draws at 2, 3, 7, 14, 21, 28, 35, and 42 days post-treatment (Supplementary Figure 1). Based on parasite presence over the course of follow-up, participants were classified as having adequate clinical and parasitological response or recurrence of parasitemia. Data from patients with recurrence of parasitemia were censored to only include time points preceding recurrence of parasitemia. Following parental or guardian written informed consent for each participant before enrollment, blood samples were collected on Whatman 903 filter paper, dried overnight, and stored in individual plastic bags with desiccant. The dried blood spots (DBS) were transported at ambient temperature to Centers for Disease Control and Prevention (CDC) laboratories in Atlanta and processed at CDC within 12 months post-collection in the field. A malaria naive panel of blood specimens was acquired from blood donors without history of travel to malaria endemic regions.

### Antigens in Multiplex Bead Assay Panel and Covalent Binding to Beads

Antibody response to a panel of 37 antigens was measured (Supplementary Table 1). These included 32 antigens from all stages of the *P. falciparum* parasite in the human host: sporozoite, hepatic (liver) stage, erythrocytic (blood) stage, and gametocyte (sexual) stage. The panel also included *P. vivax* and *P. malariae* MSP1-19 antigens to represent other human malarias for which this population of Angolan children had the possibility of previous exposure. Additionally, the panel contained three non-malaria control antigens, including a non-*Plasmodium* apicomplexan [SAG2A antigen from *Toxoplasma gondii* (33)], a disease without natural humoral immunity [tetanus toxoid from *Clostridium tetani* (34), Massachusetts Biological Laboratories, Boston, MA], and a generic laboratory purification protein (glutathione-*S*-transferase, GST, from *Schistosoma japonicum*) as an internal assay control. Parameter estimates were generated for these control antigens as well for comparative purposes.

Antigens were previously optimized for coupling conditions and covalently bound to magnetic microbeads (MagPlex, Luminex Corp., Austin, TX) by the EDC/Sulfo-NHS intermediate reaction as described previously (35). Reactive esters were formed on the carboxylated beads in the presence of 5 mM EDAC [1-Ethyl-3-(3′-dimethylaminopropyl)carbodiimide](EMD Millipore) and 5 mM Sulfo-NHS [N-hydroxysulfosuccinimide] (ThermoScientific) under rotation for 20 min. Carboxyl to antigen amine crosslinking took place in buffers and concentrations which had previously been optimized for each antigen (Supplementary Table 1) either at pH 5 (0.85% NaCl and 0.05 M 4-morpholineethanesulfonic acid, MES) or at pH 7.2 (phosphate buffered saline, PBS) under rotation for 2 h. Non-specific protein binding was blocked by BSA incubation (PBS pH 7.2, +1% BSA) for 30 min and beads were resuspended in blocking buffer (PBS, 0.05% Tween20, 1% BSA) with the addition of 0.02% NaN_3_ and protease inhibitors as described previously (35).

### Dried Blood Spot Processing and Antibody Detection Immunoassay

A 6 mm circular punch of the dried blood spot (DBS) corresponding to approximately 10 μL whole blood (and 5 uL serum with the assumption of 50% hematocrit) was taken from the center of each DBS for elution. DBS punches were placed in elution buffer which consisted of PBS, 0.5% Polyvinyl alcohol (Sigma), 0.8% Polyvinylpyrrolidine (Sigma), 0.1% casein (ThermoFisher), 0.5% BSA (Millipore), 0.3% Tween-20, 0.05% sodium azide, and 3 ug/mL *E. coli* extract to prevent non-specific binding. This elution step diluted the samples to 1:20 whole blood, which corresponded to a serum dilution of ~1:40. For the immunoassay, samples were added to assay plates at a final serum dilution of 1:100.

Separate immunoassays were performed for the same samples to detect and quantify IgA, IgD, IgE, IgM, IgG_1_, IgG_2_, IgG_3_, and IgG_4_ responses. For washing between incubation steps, plates were affixed to a plate magnet (Luminex Corp.) and gently tapped for 1–2 min before evacuating liquid. A mixture of all antigen conjugated beads was prepared in reagent diluent (PBS, 0.05% Tween20, 0.5% BSA, 0.025% NaN_3_), pipetted to each well (~625 beads per well per region) on assay plate (BioPlex Pro plates, BioRad, Hercules, CA), and washed 2 ×. Beads were incubated with 50 μL samples in assay wells for 90 min under gentle shaking at room temperature protected from light. After 3 washes, beads were incubated with 50 μL biotinylated detection antibodies specific for each Ig class or subclass at a concentration of 1:500 for 45 min (Southern Biotech, Birmingham, AL: IgA, 9130; IgM, 9020; IgD, 9030; IgE, 9250; IgG_1_, 9052; IgG_2_, 9060; IgG_3_, 9210; IgG_4_, 9200). Following 3 washes, beads were incubated with 50 μL streptavidin-phycoerythrin (Invitrogen, Waltham, MA) at a concentration of 1:200 for 30 min. Following 3 washes, beads were incubated with 50 μL reagent diluent for 30 min to remove any loosely bound antibodies. After one wash, beads were resuspended in 100 μL PBS, briefly shaken, and read immediately on a MAGPIX® machine (Luminex Corp.) by collecting the median fluorescence intensity (MFI) for a target of 50 beads per region. From blank wells on each plate, the background (bg) assay signal was removed from each samples' MFI value, to provide a final assay signal of MFI-bg which was used for analysis.

### Statistical Analysis

**Figure 1:**
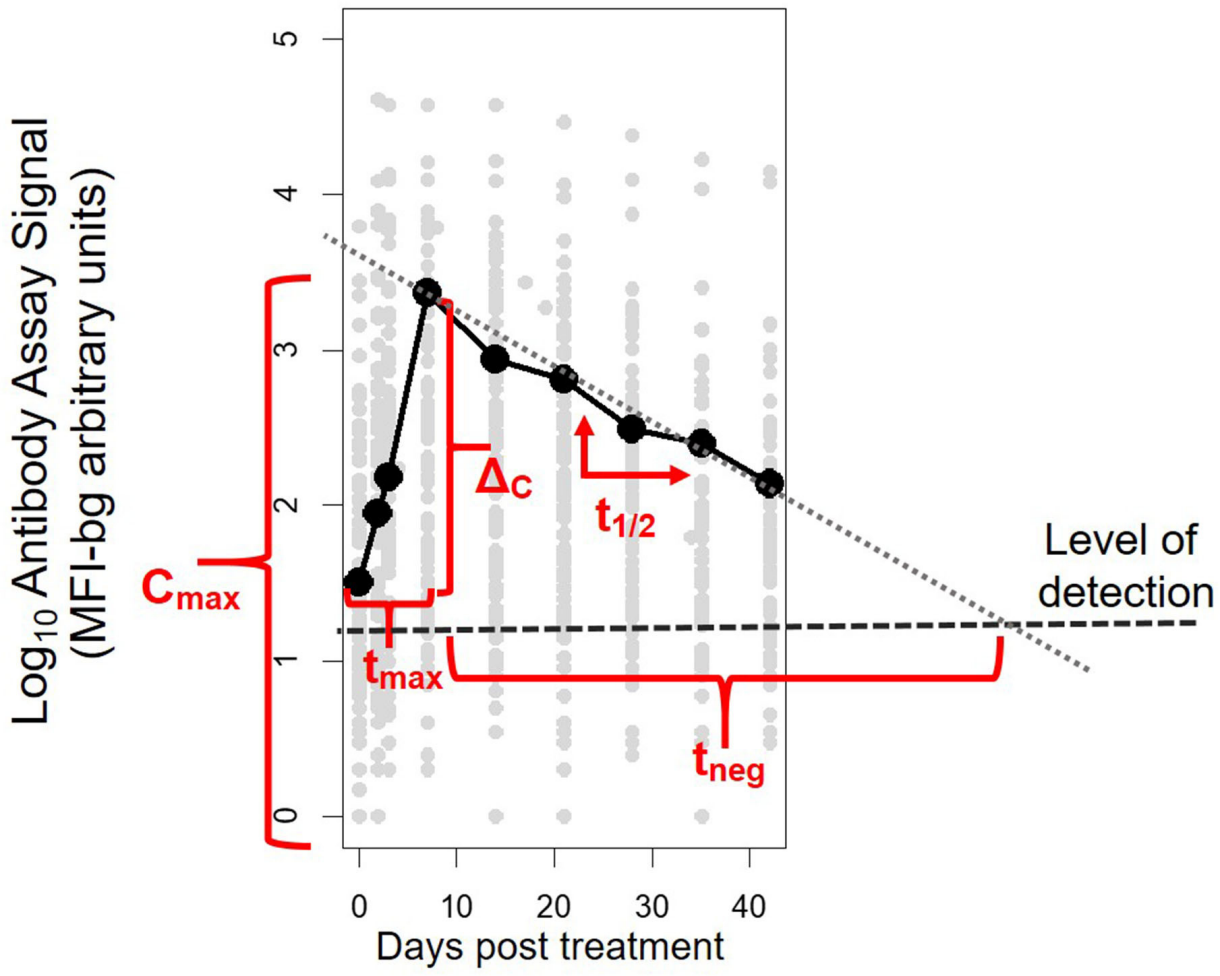
Illustrative example of the five parameters created to characterize post-treatment antibody (Ab) dynamics to *Plasmodium* antigens for each individual. The parameters represent: C_max_, maximum Ab signal; Δ_C_, maximum change in Ab signal; t_max_, time to maximum Ab signal; t_1/2_, half-life of Ab signal (in days) after maximum signal has been reached; and t_neg_, modeled time until signal is reduced to below the level of detection of the assay.

Antibody assay signal vs. patient time from initiation of study enrollment (first day of receipt of antimalarials) was plotted by antigen and antibody class/subclass. A LOESS non-parametric curve was fit to model the expected dynamics of antibody acquisition and decay. For each patient's time course for each antigen and antibody isotype/subclass, five parameters were calculated: C_max_, the maximum antibody concentration (as indicated by the assay fluorescence signal); Δ_C_, the difference between C_max_ and the antibody concentration at Day 0, t_max_, the time to reach maximum concentration; t_1/2_, the post-peak half-life assuming first-order decay kinetics; and t_neg_, the expected number of days until the signal would be expected to be below the level of detection of the assay (parameters illustrated in Figure 1). To estimate a level of detection for these specific assay conditions, for each antibody response against each antigen, a panel of 96 serum specimens from persons without history of travel to malaria endemic regions was assayed at 1:100 concentration under the same assay conditions as described above. The lognormal mean plus 3 standard deviations for the assay signals of this sample set was used at the MFI-bg threshold to denote a true positive assay signal for each Ig and antigen.

The distributions of the five parameters were plotted separately for each antibody class/subclass across all antigens. The distributions of the parameters were compared using the Kolmogorov-Smirnov-test, with IgG_1_ as the reference distribution (as the Ig consistently exhibiting the highest assay signals). Finally, the distribution of the parameters was compared for different antigens, stratifying by antibody class/subclass. All analysis was done in R version 3.6.0 (R Foundation for Statistical Computing, Austria, Vienna).

### Ethical Review

Study participants consented to collection of malaria data from provided blood samples. Secondary analysis of anonymized samples was approved by the office of the Associate Director of Science in the Center for Global Health at the CDC (Project ID: 0900f3eb8193aa9d).

## Results

### Serological Panel and Enrolled Participants

A panel of 37 antigens expressed by *P. falciparum* and other pathogens (Supplementary Table 1) was utilized for multiplex antibody detection in blood samples. A total of 889 samples from time courses for 104 patients were assayed for antibodies with patient characteristics provided in Supplementary Table 2. The median age of enrollment was 3 years (range: 0.5–11) and median parasite density at enrollment (baseline; Day 0) was 22,065 parasites/μL blood (range: 3,916–200,000). Enrollment was the day the child presented to the health facility with symptomatic malaria. Approximately half of all patients were female (47%), and all were microscopy negative for malaria parasites at 3 days after initiation of malaria chemotherapy. Of the 104 patients included in this study, 89 (85.6%) provided the complete nine part follow-up blood sampling series for Days 0, 2, 3, 7, 14, 21, 28, 35, and 42 (Supplementary Figure 1).

### Development of Parameters and Examples by Specific Antigens

**Figure 2:**
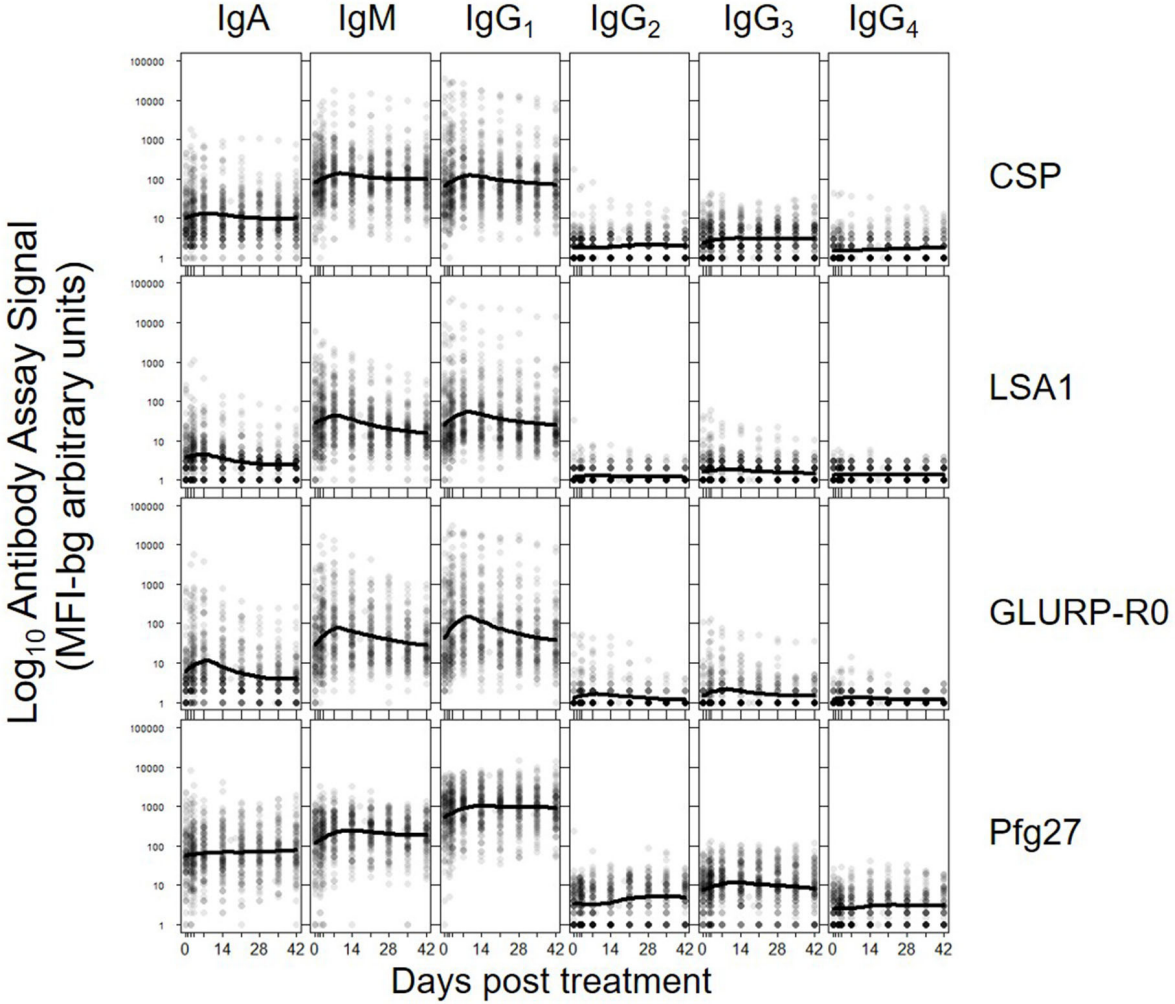
Post-treatment dynamics of antibodies stratifying by Ig isotype/subclass to: sporozoite- stage CSP, hepatic-stage LSA1, erythrocytic-stage GLURP-R0 and sexual-stage Pfg27. Points on each plot represent individual sample assay signals, and solid lines represents LOESS fitting to all assay signal data for the specific antigen and Ig class/subclass. Plots for all Igs against all antigens shown in Supplementary Figure 2.

The five parameters created for the antibody comparisons in this study are illustrated in Figure 1 and further described in Methods. While no measurable IgD or IgE binding was detected for antigens in the multiplex panel at the 1:100 serum dilution, there were measurable antibody responses of the IgA, IgM and all 4 IgG subclasses to all antigens. Figure 2 shows the post-treatment dynamics for antibodies for these classes and subclasses for four illustrative antigens spanning four biological compartments of *P. falciparum*: sporozoite CSP, hepatic-stage LSA1, erythrocytic-stage GLURP-R0, and sexual-stage Pfg27 (corresponding plots for all antigens are shown in Supplementary Figure 2). Antibody levels for IgM and all 4 IgG subclasses typically rose in the first 2 weeks post-treatment, before declining by mirroring first-order rate kinetics (evidenced by a linear decay on a semi-log plot). In contrast to IgM and IgG, IgA levels consistently showed a different pattern, rising in the first 2 weeks, hitting a nadir at 3–4 weeks post-treatment, before rising again. This unique pattern in IgA dynamics was observed for responses to nearly all malarial antigens (Supplementary Figure 2).

### Dynamics of Antibodies to Control Antigens

Antibodies to PvMSP1-19 and PmMSP1-19 also showed post-treatment increases, potentially suggestive of Ig cross-binding potential among the PfMSP1-19 antigen and other species' isoforms. Notably, there were also consistent slight rises in antibody responses to the SAG2A *Toxoplasma gondii* antigen among different Ig classes. However, Ig responses to the tetanus toxoid and the generic GST antigens were largely unchanged among the 42-day longitudinal follow-up samplings.

### Parameter Estimates by Ig Isotype

**Figure 3:**
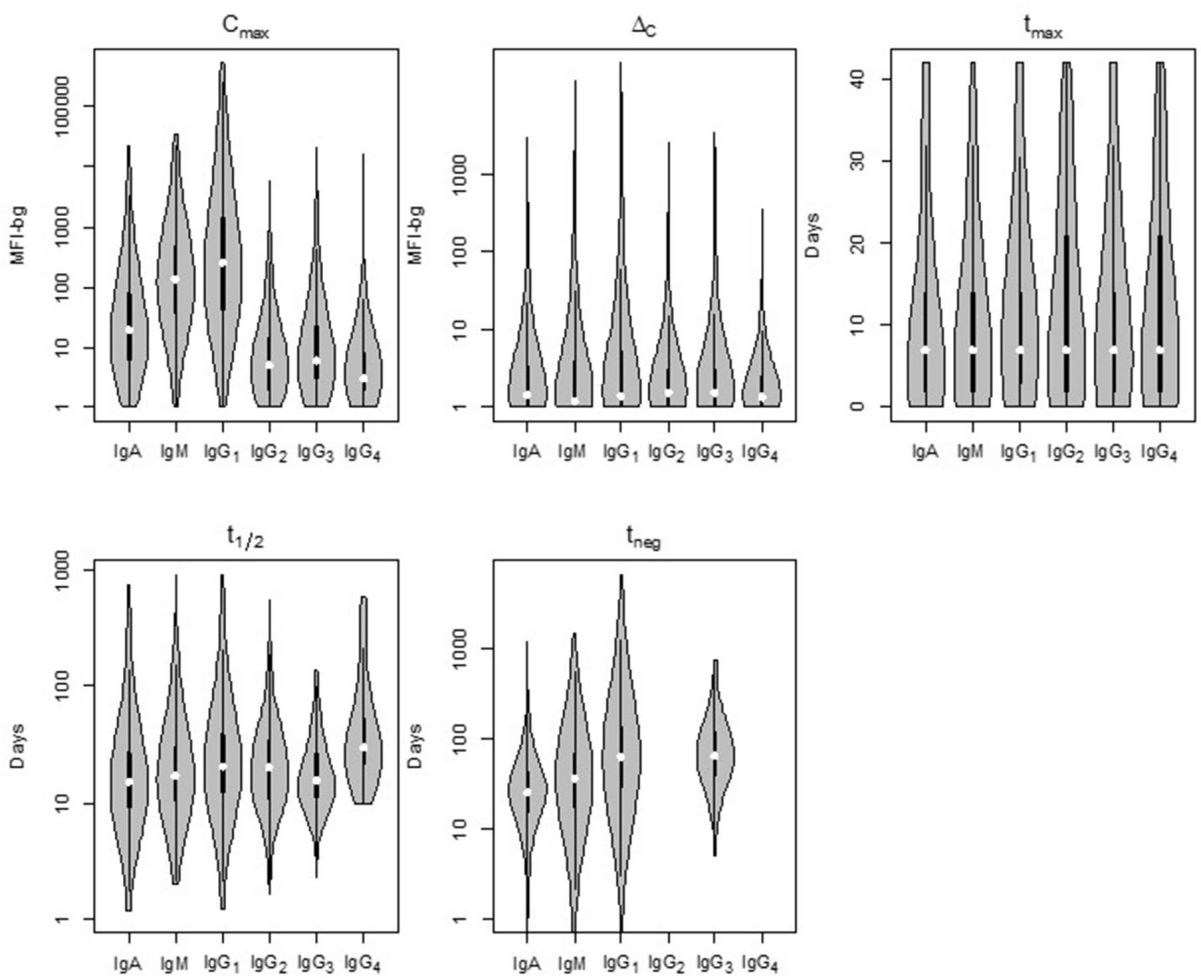
Distribution of the five post-treatment clearance parameters by Ig isotype/subclass for antibodies to malaria antigens in Angolan children treated for *Plasmodium falciparum* malaria. Violin plots display data for all *P. falciparum* antigens with the median estimate as a white point, black bars as the interquartile range (IQR), and whiskers extending 1.5 × IQR. Plots occasionally do not exist for IgG_2_ and/or IgG_4_ as not enough children samples provided quantifiable assay signals for these subclasses. Statistical comparison among isotypes shown in Supplementary Table 2. MFI-bg: median fluorescence intensity minus background assay signal.

Comparison of the five parameters by Ig isotype/subclass revealed important differences (Figure 3). Peak antibody response (C_max_) was highly variable among isotype and subclass, and statistically-significant differences was observed among the mean of all antigen responses except in comparing IgG_2_ to IgG_4_ and in comparing IgG_3_ to IgG_4_ responses (Table 1). C_max_ was generally highest for IgG_1_ followed by IgM, IgA, and the three other IgG sub-classes (in descending order: IgG_3_, IgG_2_, and IgG_4_). Amongst the IgG subclasses, the IgG_1_ C_max_ responses dwarfed the other IgG sub-classes by 1-2 orders of magnitude, and the low observed signals for IgG_2_, IgG_3_, and IgG_4_ responses precluded determination of t_1/2_ and t_neg_ for most time-courses and antigens. The median increase in antibody response (Δ_C_) was lowest for IgM, though there was a high level of variation among the antigen panel for all isotypes. Some antigens' IgG_1_ Δ_C_ response registered the largest Δ_C_ estimates for the antigen panel. Overall, the time for individuals to reach peak response for IgG_1_ antibodies (t_max_) was statistically longer (mean: 12.2 days) compared to IgA (10.0 days) and IgM (10.4 days) (*p* < 0.00067; Table 1). The modeled half-life (t_1/2_) for temporal decrease in assay signal was longest for the IgG_1_ responses compared to IgM and IgA, and this finding was reemphasized in the higher estimated persistence of detectable antibody response (t_neg_) of IgG_1_ vs. the IgM and IgA classes.

**Table 1:** Differences in empirical distributions of decay parameters by immunoglobulin class/subclass, Kolmogorov-Smirnov *p*-value with red cells highlighting statistically significant differences (p-val < 0.00067, Bonferroni corrected).

	**IgA**	**IgM**	**IgG_**1**_**	**IgG_**2**_**	**IgG_**3**_**	**IgG_**4**_**
**Test in difference between C**_**max**_						
IgA	-	0.000	0.000	0.000	0.000	0.000
IgM		-	0.000	0.000	0.000	0.000
IgG_1_			-	0.000	0.000	0.000
IgG_2_				-	0.000	0.000
IgG_3_					-	0.000
IgG_4_						-
**Test in difference between** **Δ_C_**						
IgA	-	0.000	0.000	0.004	0.012	0.000
IgM		-	0.006	0.000	0.000	0.000
IgG_1_			-	0.000	0.000	0.000
IgG_2_				-	0.164	0.009
IgG_3_					-	0.000
IgG_4_						-
**Test in difference between t**_**max**_						
IgA	-	0.000	0.000	0.000	0.000	0.000
IgM		-	0.000	0.000	0.000	0.000
IgG_1_			-	0.000	0.000	0.000
IgG_2_				-	0.000	0.188
IgG_3_					-	0.204
IgG_4_						-
**Test in difference between t**_**1/2**_						
IgA	-	0.004	0.000	0.072	0.085	0.000
IgM		-	0.000	0.517	0.282	0.000
IgG_1_			-	0.779	0.000	0.015
IgG_2_				-	0.151	0.023
IgG_3_					-	0.000
IgG_4_						-
**Test in difference between t**_**neg**_						
IgA	-	0.000	0.000	-	0.000	-
IgM		-	0.000	-	0.000	-
IgG_1_			-	-	0.037	-
IgG_2_				-	-	-
IgG_3_					-	-
IgG_4_						-

### Parameter Estimates Among 37 Antigens on Multiplex Panel

**Figure 4:**
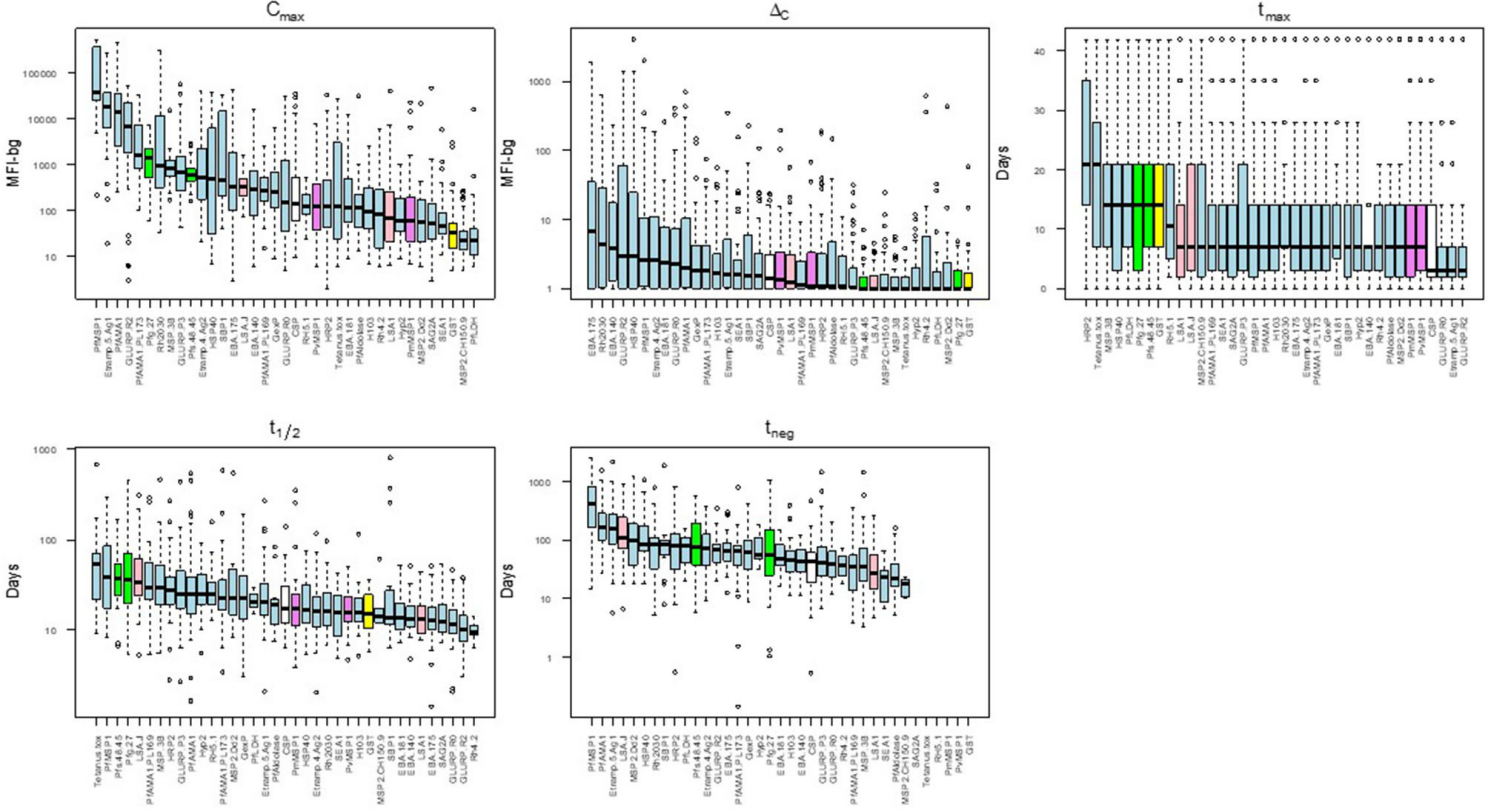
Distribution of acquisition and decay parameters for IgG_1_ to different antigens in Angolan children treated for malaria. Antigens are color-coded by category: sporozoite (white), hepatic (pink), erythrocytic (blue), gametocyte (green), non-falciparum *Plasmodium* (violet) and other control (yellow). Boxplots display median as black solid line with box length representing interquartile range (IQR) and whiskers 1.5 × IQR. Outliers above or below 1.5 × IQR represented as circles. MFI-bg: median fluorescence intensity-background. Plots for parameter estimates for IgM, IgA, and IgG_3_ shown in Supplementary Figures 3–5, respectively.

There was substantial variability in the five summary parameters when comparing estimates for the different individual antigens. Figure 4 shows the distribution of the five parameters for the IgG_1_ response as measured against all 37 antigens in this study (corresponding figures for the other isotypes/subclasses are shown in Supplementary Figures 3–5). The maximum assay signal (C_max_) showed large variation across different antigens, spanning >4 orders of magnitude for IgG_1_. The highest median IgG_1_ C_max_ was to PfMSP1-19, followed by Etramp 5 ag1, and PfAMA1, and these three antigens also provoked the strongest IgA, IgM, and IgG_3_ responses. The maximum observed increase in antibody response (Δ_C_) was highest for the *P. falciparum* erythrocyte-stage antigens among all Ig classes and sub-classes. For IgG_1_ and IgG_3_, time to maximum concentration (t_max_) was longest for HRP2, with median t_max_ of 21 days for both subclasses. The t_max_ for IgG_1_ was shortest for GLURP-R2 which peaked on overage only 3 days post-treatment, with nearly all individual responses peaking before 7 days. Estimated Ig half-lives against different antigens spanned 2 orders of magnitude for IgG_1_ but remained within one order of magnitude for the other Ig isotypes/subclasses. Excluding the control antigens, the longest t_1/2_ was observed in antibodies against PfMSP1-19, with a median of 38 days, and the shortest for antibodies to the two GLURP antigens and Rh4.2 (10–12 days). Post-treatment duration of antibody positivity (t_neg_) followed this similar pattern for IgG_1_, with a median of 408 days of seropositivity for PfMSP1-19, more than double the next-most persistent response (153 days for PfAMA1). For all t_neg_ estimates among different isotypes/subclasses, antigens representing the different stages of human *P. falciparum* infection did not show generalizable trends by infection stage and are intermingled throughout. Selected examples of the personal variation in Ig dynamics are given for single persons' Ig response to multiple antigens across the 42-day follow-up (Supplementary Figure 6).

## Discussion

This study investigated the binding of all isotypes of human antibodies (and 4 subclasses of IgG) to a panel of 32 *P. falciparum* antigens in children treated for *P. falciparum* infection and followed up for 42 days. As only children older than 6 months were enrolled in this study (32), and evidence has been shown that any maternal-derived antibodies have waned by this point in life (36, 37), the antibody data presented here would be reasonably assumed to come from natural malaria exposure and host adaptive immune responses. These results reveal stark heterogeneities in antibody dynamics across different antigens, among Ig isotype/subclass, and among individual persons. At a 1:100 serum dilution, no measurable IgD or IgE binding was detected for any of the *P. falciparum* or control antigens, but IgA, IgM and all 4 IgG subclasses were ubiquitously detected with the exception of IgG_2_ and IgG_4_. Five response parameters were created and estimated by this study to allow systematic comparison of magnitude of response as well as the shape of the acquisition and decay curve among antigens, antibody isotypes, and persons. Among antibody classes, and even within the same class (i.e., IgG_1_), there was substantial variability across the 32 *P. falciparum* antigens [as suggested in some studies in other populations (38, 39)] with all key parameters describing the shape of the antibody dynamics varying by antigen, often over several orders of magnitude. These data reinforce many of the classical assumptions regarding the anti-pathogen humoral response to bloodborne pathogens in terms of both Ig class dynamics and interpersonal variability. Interestingly, IgM decay was not markedly more rapid than IgG, similar to a recent report in other populations (40). Furthermore, for the set of antigens in this panel, the estimated half-life (t_1/2_) for IgG_3_ was not shorter than IgG_1_, despite having an intrinsically quicker clearance from blood.

This heterogeneity in the five parameters could potentially be beneficial to the study of natural (or induced) immunity to malaria, as it opens the possibility of picking and choosing different Ig classes and antigens to design assays aimed at characterizing previous malaria exposure. Our results suggest antibodies to PfMSP1-19, a known marker for immunity against *P. falciparum* (41), may be a putative “long-term” marker of exposure to *P. falciparum* with IgG antibodies predicted to last for years after infection (42). Individuals had robust responses to PfMSP1-19 and the aggregated estimate of the IgG_1_ half-life was 38 days, suggesting that individuals with exposure would retain seropositivity several 100 days after the infection. Similarly, PfAMA1 elicited a similarly strong IgG_1_ response with a long half-life estimates (range: 23–30 days). Other antigens such as Rh2_2020 and Etramp 5 ag 1 showed nearly as strong responses, but the half-life of these responses was substantially shorter, on the order of 16–21 days. On the other end of the spectrum, responses to the GLURP antigens were also substantial, but with rapid decay. An estimated half-life of around 10 days suggests that the presence of a strong GLURP response is evidence of active or very recent infection (43). Some vaccine candidates showed good antibody induction by infection (e.g., PfAMA1, Rh2, PfMSP1, GLURP), whereas others had a weaker response (e.g., RH5, CSP), which has been reported in other populations (39). Other groups have also presented estimates for antibody half-life as well as time to seronegativity in other populations. A previous 3-year longitudinal study of naturally-exposed Kenyan children estimated total IgG against PfAMA1 and PfMSP2 antigens would have a half-life of approximately 1 year or longer, and the majority of children were found to retain seropositivity to these two antigens 1 year after no detectable *P. falciparum* infections (44). However, even only measuring IgG against this smaller panel of antigens, high interpersonal variation was still observed in these study populations (45). This study expands the analytical capacity of predictive Ig response for vaccine or seroepidemiological studies by introducing standardized parameters in which to compare among a broad panel of 32 *P. falciparum* antigens among all human Ig isotypes.

The results revealed several unreported patterns in the antibody dynamics following clearance of *P. falciparum* infection. First, IgA responses against many antigens appear to be biphasic, with a secondary increase in IgA response 3–4 weeks post-treatment—thus not fitting well to a monotonic first-order decay assumption. Second, we observed evidence of non-specific Ig signals, with increases in non-*P. falciparum* malaria and non-*Plasmodium* spp. responses for all Ig isotypes/subclasses. Some limited cross-reactivity between responses to the *Plasmodium* spp. MSP1-19 antigens has been shown before (35, 46), so the increase in *Plasmodium* spp. MSP1 responses is likely a combination of non-specific immune activation and cross-binding among homologs.

The estimates for the parameters presented here are not absolute, as the specific assay conditions used in this study could confound the results, precluding definitive conclusions regarding the expected antibody clearance dynamics. For the assay, blood dried on filter paper was the only sample type utilized, and potential exists for reduction of binding potential of Ig after blood desiccation, though this sample type has been shown to be robust in previous studies (35, 46, 47). Additionally, the highest concentration of serum used in this assay was 1:100, which may explain why anti-malarial IgE was not detected in this current study whereas it has been observed in low quantities in previous malaria serostudies (48, 49), and that IgG_2_ and IgG_4_ were frequently undetectable in these samples. The multiplex assay beads used in the study were coupled at antigen concentrations optimized by our group for each antigen, so direct comparison of one antigen's assay signal to another is not necessarily an absolute indication of the concentration of those antibodies in a blood sample. Regarding the host side, neither the number of previous malaria episodes nor the duration of the current infection was known for the participants, and thus the antibody dynamics described here are potentially a conflation of responses in both naive and non-naive individuals with various degrees of immunological memory, different durations of *P. falciparum* infection, and different antibody affinities. Given this heterogeneity in host factors, it is therefore even more remarkable that clear patterns emerge from the data. The maximum duration of follow-up was only 42 days, and thus the antibody dynamics beyond this time point could not be characterized with specimen data and needed to be predictively modeled. Specifically, what happens to IgA responses for all antigens and the IgG responses to HRP2, both of which showed tendencies to increase prior to day 42, remain unanswered. Other groups have followed malaria convalescent individuals or persons immunized with malaria antigens for longer periods of time (14, 19, 50), and generation of these five parameter estimates from those datasets of longer study periods could provide a comparison to estimates obtained here. A similarity among all enrolled participants in this study was the relative young age of this study population with the median age of 3 years and the oldest participant at 11 years old. The findings presented here should be interpreted in light of this study population, as the humoral immune responses in this young population may not be representative of other study populations with a broader range of ages and living under different transmission pressures.

Similar analyses on samples from patients followed during other longitudinal studies in additional settings and of other ages, and/or samples from controlled human infections, could serve as a comparator dataset to determine if consistency would exist in these parameter estimates for the same antigens among different studies and populations. The present study highlights the advantage of high-throughput antibody detection using the multiplex bead-based assay and the amount of antibody data that could be collected for an individual specimen. Overall, being able to have predictive capacity around the humoral response of a populace to *P. falciparum* infection can provide the best information and assumptions needed for seroepidemiological modeling and outputs to estimate falciparum malaria in a population. In utilizing a multiplex assay format, multiple targets, and multivariate analyses, an antibody profile could be created for an individual which precisely estimates (with confidence intervals) the time since last *P. falciparum* exposure, and this information at community and population levels would be valuable for estimation of historical transmission in an area. With being able to create parameters for both the acquisition of, as well as loss, of Ig signal following *P. falciparum* infection, this work moves toward a scenario where investigators can infer the timing of the last *P. falciparum* exposure with increased accuracy and precision based on serology alone.

## Data Availability Statement

The raw data supporting the conclusions of this article will be made available by the authors, without undue reservation.

## Ethics Statement

The studies involving human participants were reviewed and approved by Associate Director of Science in the Center for Global Health at the CDC (Project ID: 0900f3eb8193aa9d). Written informed consent to participate in this study was provided by the participants' legal guardian/next of kin.

## Author Contributions

PD and MP designed coordinated the field study. ER and MP designed the laboratory study, conceptualized the experiments, drafted the manuscript, and performed statistical analyses. ER and DN performed laboratory assays. BW, JP, JB, CD, and KT provided antigens and scientific expertise. All authors reviewed and approved the final version of the manuscript.

## Conflict of Interest

The authors declare that the research was conducted in the absence of any commercial or financial relationships that could be construed as a potential conflict of interest.

## References

[1] Outlaw DC Ricklefs RE. Rerooting the evolutionary tree of malaria parasites. Proc Natl Acad Sci USA. (2011) 108:13183–7. 10.1073/pnas.1109153108 21730128 PMC3156215

[2] Bensch S Hellgren O Krizanauskiene A Palinauskas V Valkiunas G Outlaw D. How can we determine the molecular clock of malaria parasites? Trends Parasitol. (2013) 29:363–9. 10.1016/j.pt.2013.03.011 23648374

[3] Tse EG Korsik M Todd MH. The past, present and future of anti-malarial medicines. Malar J. (2019) 18:93. 10.1186/s12936-019-2724-z 30902052 PMC6431062

[4] Kwiatkowski DP. How malaria has affected the human genome and what human genetics can teach us about malaria. Am J Hum Genet. (2005) 77:171–92. 10.1086/432519 16001361 PMC1224522

[5] Stevenson MM Riley EM. Innate immunity to malaria. Nat Rev Immunol. (2004) 4:169–80. 10.1038/nri1311 15039754

[6] Perkins DJ Were T Davenport GC Kempaiah P Hittner JB Ong'echa JM. Severe malarial anemia: innate immunity and pathogenesis. Int J Biol Sci. (2011) 7:1427–42. 10.7150/ijbs.7.1427 22110393 PMC3221949

[7] Beeson JG Drew DR Boyle MJ Feng G Fowkes FJ Richards JS. Merozoite surface proteins in red blood cell invasion, immunity and vaccines against malaria. FEMS Microbiol Rev. (2016) 40:343–72. 10.1093/femsre/fuw001 26833236 PMC4852283

[8] Wolf AS Sherratt S Riley EM. NK Cells: uncertain allies against malaria. Front Immunol. (2017) 8:212. 10.3389/fimmu.2017.00212 28337195 PMC5343013

[9] Gowda DC Wu X. Parasite recognition and signaling mechanisms in innate immune responses to malaria. Front Immunol. (2018) 9:3006. 10.3389/fimmu.2018.03006 30619355 PMC6305727

[10] Hoffman SL Nussenzweig V Sadoff JC Nussenzweig RS. Progress toward malaria preerythrocytic vaccines. Science. (1991) 252:520–1. 10.1126/science.2020852 2020852

[11] Wipasa J Riley EM. The immunological challenges of malaria vaccine development. Expert Opin Biol Ther. (2007) 7:1841–52. 10.1517/14712598.7.12.1841 18034650

[12] Lyke KE. Steady progress toward a malaria vaccine. Curr Opin Infect Dis. (2017) 30:463–70. 10.1097/QCO.0000000000000393 28731898

[13] Corran P Coleman P Riley E Drakeley C. Serology: a robust indicator of malaria transmission intensity? Trends Parasitol. (2007) 23:575–82. 10.1016/j.pt.2007.08.023 17988945

[14] Helb DA Tetteh KK Felgner PL Skinner J Hubbard A Arinaitwe E. Novel serologic biomarkers provide accurate estimates of recent *Plasmodium falciparum* exposure for individuals and communities. Proc Natl Acad Sci USA. (2015) 112:E4438–47. 10.1073/pnas.1501705112 26216993 PMC4538641

[15] Kwenti TE Kukwah TA Kwenti TDB Nyassa BR Dilonga MH Enow-Orock G. Comparative analysis of IgG and IgG subclasses against *Plasmodium falciparum* MSP-119 in children from five contrasting bioecological zones of Cameroon. Malar J. (2019) 18:16. 10.1186/s12936-019-2654-9 30670064 PMC6341684

[16] Coggeshall L Kumm H. Demonstration of passive immunity in experimental monkey malaria. J Exp Med. (1937) 66:177–90. 10.1084/jem.66.2.177 19870655 PMC2133598

[17] Coggeshall L. The occurrence of malaria antibodies in human serum following induced infection with Plasmodium knowlesi. J Exp Med. (1940) 72:21. 10.1084/jem.72.1.21 19871005 PMC2135013

[18] Kuvin SF Tobie JE Evans CB Coatney GR Contacos PGJS. Antibody production in human malaria as determined by the fluorescent antibody technique. Science. (1962) 135:1130–1. 10.1126/science.135.3509.1130 14460984

[19] Crompton PD Kayala MA Traore B Kayentao K Ongoiba A Weiss GE. A prospective analysis of the Ab response to *Plasmodium falciparum* before and after a malaria season by protein microarray. Proc Natl Acad Sci USA. (2010) 107:6958–63. 10.1073/pnas.1001323107 20351286 PMC2872454

[20] Richards JS Arumugam TU Reiling L Healer J Hodder AN Fowkes FJ. Identification and prioritization of merozoite antigens as targets of protective human immunity to *Plasmodium falciparum* malaria for vaccine and biomarker development. J Immunol. (2013) 191:795–809. 10.4049/jimmunol.1300778 23776179 PMC3702023

[21] Kerkhof K Canier L Kim S Heng S Sochantha T Sovannaroth S. Implementation and application of a multiplex assay to detect malaria-specific antibodies: a promising tool for assessing malaria transmission in Southeast Asian pre-elimination areas. Malar J. (2015) 14:338. 10.1186/s12936-015-0868-z 26337785 PMC4558921

[22] Fonseca AM Quinto L Jiménez A González R Bardají A Maculuve S. Multiplexing detection of IgG against *Plasmodium falciparum* pregnancy-specific antigens. PLoS ONE. (2017) 12:e0181150. 10.1371/journal.pone.0181150 28715465 PMC5513451

[23] Bergmann-Leitner ES Mease RM De La Vega P Savranskaya T Polhemus M Ockenhouse C. Immunization with pre-erythrocytic antigen CelTOS from *Plasmodium falciparum* elicits cross-species protection against heterologous challenge with Plasmodium berghei. PLoS ONE. (2010) 5:e12294. 10.1371/journal.pone.0012294 20808868 PMC2924390

[24] Oyen D Torres JL Wille-Reece U Ockenhouse CF Emerling D Glanville J. Structural basis for antibody recognition of the NANP repeats in *Plasmodium falciparum* circumsporozoite protein. Proc Natl Acad Sci USA. (2017) 114:E10438–45. 10.1073/pnas.1715812114 29138320 PMC5715787

[25] Mikolajczak SA Sacci J John B De La Vega P Camargo N Vanbuskirk K. Disruption of the *Plasmodium falciparum* liver-stage antigen-1 locus causes a differentiation defect in late liver-stage parasites. Cell Microbiol. (2011) 13:1250–60. 10.1111/j.1462-5822.2011.01617.x 21569184 PMC4155577

[26] Ouédraogo AL Eckhoff PA Luty AJ Roeffen W Sauerwein RW Bousema T. Modeling the impact of *Plasmodium falciparum* sexual stage immunity on the composition and dynamics of the human infectious reservoir for malaria in natural settings. PLoS Pathog. (2018) 14:e1007034. 10.1371/journal.ppat.1007034 29742161 PMC5962096

[27] Murugan R Buchauer L Triller G Kreschel C Costa G Martí GP. Clonal selection drives protective memory B cell responses in controlled human malaria infection. Sci Immunol. (2018) 3:eaap8029. 10.1126/sciimmunol.aap8029 29453292

[28] Silveira EL Dominguez MR Soares IS. To B or not to B: understanding B cell responses in the development of malaria infection. Front Immunol. (2018) 9:2961. 10.3389/fimmu.2018.02961 30619319 PMC6302011

[29] Portugal S Pierce SK Crompton PD. Young lives lost as B cells falter: what we are learning about antibody responses in malaria. J Immunol. (2013) 190:3039–46. 10.4049/jimmunol.1203067 23526829 PMC3608210

[30] Portugal S Tipton CM Sohn H Kone Y Wang J Li S. Malaria-associated atypical memory B cells exhibit markedly reduced B cell receptor signaling and effector function. Elife. (2015) 4:e07218. 10.7554/eLife.07218.017 25955968 PMC4444601

[31] Pérez-Mazliah D Gardner PJ Schweighoffer E Mclaughlin S Hosking C Tumwine I. Plasmodium-specific atypical memory B cells are short-lived activated B cells. Elife. (2018) 7:e39800. 10.7554/eLife.39800 30387712 PMC6242553

[32] Davlantes E Dimbu PR Ferreira CM Joao MF Pode D Félix J. Efficacy and safety of artemether-lumefantrine, artesunate-amodiaquine, and dihydroartemisinin-piperaquine for the treatment of uncomplicated *Plasmodium falciparum* malaria in three provinces in Angola, 2017. Malar J. (2018) 17:144. 10.1186/s12936-018-2290-9 29615039 PMC5883595

[33] Priest JW Moss DM Arnold BF Hamlin K Jones CC Lammie PJ. Seroepidemiology of Toxoplasma in a coastal region of Haiti: multiplex bead assay detection of immunoglobulin G antibodies that recognize the SAG2A antigen. Epidemiol Infect. (2015) 143:618–30. 10.1017/S0950268814001216 25600668 PMC5844480

[34] Scobie HM Mao B Buth S Wannemuehler KA Sorensen C Kannarath C. Tetanus immunity among women aged 15 to 39 years in Cambodia: a national population-based serosurvey, 2012. Clin Vaccine Immunol. (2016) 23:546–54. 10.1128/CVI.00052-16 27053629 PMC4933773

[35] Plucinski MM Candrinho B Chambe G Muchanga J Muguande O Matsinhe G. Multiplex serology for impact evaluation of bed net distribution on burden of lymphatic filariasis and four species of human malaria in northern Mozambique. PLoS Neglected Trop Dis. (2018) 12:e0006278. 10.1371/journal.pntd.0006278 29444078 PMC5854460

[36] Kangoye DT Nebie I Yaro JB Debe S Traore S Ouedraogo O. *Plasmodium falciparum* malaria in children aged 0-2 years: the role of foetal haemoglobin and maternal antibodies to two asexual malaria vaccine candidates (MSP3 and GLURP). PLoS ONE. (2014) 9:e107965. 10.1371/journal.pone.0107965 25238160 PMC4169582

[37] Dent AE Malhotra I Wang X Babineau D Yeo KT Anderson T. Contrasting patterns of serologic and functional antibody dynamics to *Plasmodium falciparum* antigens in a Kenyan birth cohort. Clin Vaccine Immunol. (2016) 23:104–16. 10.1128/CVI.00452-15 26656119 PMC4744923

[38] Stanisic DI Good MF. Whole organism blood stage vaccines against malaria. Vaccine. (2015) 33:7469–75. 10.1016/j.vaccine.2015.09.057 26428451

[39] Mccallum FJ Persson KE Fowkes FJ Reiling L Mugyenyi CK Richards JS. Differing rates of antibody acquisition to merozoite antigens in malaria: implications for immunity and surveillance. J Leukoc Biol. (2017) 101:913–25. 10.1189/jlb.5MA0716-294R 27837017 PMC5346181

[40] Boyle MJ Chan JA Handayuni I Reiling L Feng G Hilton A. IgM in human immunity to *Plasmodium falciparum* malaria. Sci Adv. (2019) 5:eaax4489. 10.1126/sciadv.aax4489 31579826 PMC6760923

[41] Fowkes FJ Richards JS Simpson JA Beeson JG. The relationship between anti-merozoite antibodies and incidence of *Plasmodium falciparum* malaria: a systematic review and meta-analysis. PLoS Med. (2010) 7:e1000218. 10.1371/journal.pmed.1000218 20098724 PMC2808214

[42] Ondigo BN Hodges JS Ireland KF Magak NG Lanar DE Dutta S. Estimation of recent and long-term malaria transmission in a population by antibody testing to multiple *Plasmodium falciparum* antigens. J Infect Dis. (2014) 210:1123–32. 10.1093/infdis/jiu225 24737801 PMC4168304

[43] Van Den Hoogen LL Stresman G Presume J Romilus I Mondelus G Elisme T. Selection of antibody responses associated with *Plasmodium falciparum* infections in the context of malaria elimination. Front Immunol. (2020) 11:928. 10.3389/fimmu.2020.00928 32499783 PMC7243477

[44] Mugyenyi CK Elliott SR Yap XZ Feng G Boeuf P Fegan G. Declining malaria transmission differentially impacts the maintenance of humoral immunity to *Plasmodium falciparum* in children. J Infect Dis. (2017) 216:887–98. 10.1093/infdis/jix370 28973483 PMC5853596

[45] Kinyanjui SM Bejon P Osier FH Bull PC Marsh K. What you see is not what you get: implications of the brevity of antibody responses to malaria antigens and transmission heterogeneity in longitudinal studies of malaria immunity. Malar J. (2009) 8:242. 10.1186/1475-2875-8-242 19860926 PMC2773787

[46] Priest JW Plucinski MM Huber CS Rogier E Mao B Gregory CJ. Specificity of the IgG antibody response to *Plasmodium falciparum, Plasmodium vivax, Plasmodium malariae*, and *Plasmodium ovale* MSP1 19 subunit proteins in multiplexed serologic assays. Malar J. (2018) 17:417. 10.1186/s12936-018-2566-0 30413163 PMC6230236

[47] Corran PH Cook J Lynch C Leendertse H Manjurano A Griffin J. Dried blood spots as a source of anti-malarial antibodies for epidemiological studies. Malar J. (2008) 7:195. 10.1186/1475-2875-7-195 18826573 PMC2567984

[48] Ubillos I Jimenez A Vidal M Bowyer PW Gaur D Dutta S. Optimization of incubation conditions of *Plasmodium falciparum* antibody multiplex assays to measure IgG, IgG1-4, IgM and IgE using standard and customized reference pools for sero-epidemiological and vaccine studies. Malar J. (2018) 17:219. 10.1186/s12936-018-2369-3 29859096 PMC5984756

[49] Vidal M Aguilar R Campo JJ Dobano C. Development of quantitative suspension array assays for six immunoglobulin isotypes and subclasses to multiple *Plasmodium falciparum* antigens. J Immunol Methods. (2018) 455:41–54. 10.1016/j.jim.2018.01.009 29397157 PMC5843563

[50] Sirima SB Durier C Kara L Houard S Gansane A Loulergue P. Safety and immunogenicity of a recombinant *Plasmodium falciparum* AMA1-DiCo malaria vaccine adjuvanted with GLA-SE or Alhydrogel(R) in European and African adults: a phase 1a/1b, randomized, double-blind multi-centre trial. Vaccine. (2017) 35:6218–27. 10.1016/j.vaccine.2017.09.027 28947345

