# Supplementary material for: Framework for Characterizing Longitudinal Antibody Response in Children After Plasmodium falciparum Infection
Source: Front Immunol. 2021 Mar 2;12:617951. doi: 10.3389/fimmu.2021.617951 (PMC7960919; doi:10.3389/fimmu.2021.617951)

***Supplementary Material***

**Supplementary Table 1.** Laboratory information and references for antigen panel.

| **Antigen**  **Alias** | ***P. falciparum* Host infection stage** | **Full name and information** | **Production** | **Expression**  **Tag** | **Conditions for Bead**  **Conjugation** | **Source**  **References** |  |
| --- | --- | --- | --- | --- | --- | --- | --- |
| PfCSP | Sporozoite | Circumsporozoite protein NANP repeat | Peptide | Crosslinked with GST | 50mM MES at pH 5, 60ug/mL | (Priest et al., 2018) |  |
| LSA1 | Hepatocyte | Liver-stage antigen PL1043 epitope | Peptide | NA* | 50mM MES at pH 5, 30ug/mL | (Yang et al., 1995) |  |
| LSA.J | Hepatocyte | Liver-stage antigen C-terminal region | Peptide | NA | ADH modification^†^, 100mM MES^#^ at pH 6, 10ug/mL | (Fidock et al., 1994) |  |
| MSP2_CH150/9 | Erythrocyte | Merozoite surface protein 2 | Recombinant, *E. coli* | GST | 50mM MES at pH 5, 5.0ug/mL | (Osier et al., 2008) |  |
| MSP2_Dd2 | Erythrocyte | Merozoite surface protein 2 | Recombinant, *E. coli* | GST | 50mM MES at pH 5, 20ug/mL | (Osier et al., 2008) |  |
| MSP.3B | Erythrocyte | Merozoite surface protein 3, non-repeat region | Peptide | NA | ADH modification, 100mM MES at pH 6, 10ug/mL | (Mahajan et al., 2010) |  |
| PfMSP1-19 | Erythrocyte | Merozoite surface protein 1, 19kD | Recombinant, *E. coli* | GST | 50mM MES at pH 5, 20ug/mL | (Rogier et al., 2015) |  |
| PfAMA1 | Erythrocyte | Apical membrane antigen 1, N terminal region | Recombinant, *E. coli* | GST | 50mM MES at pH 5, 30ug/mL | (Dutta et al., 2002) |  |
| PfAMA1.PL173 | Erythrocyte | Apical membrane antigen 1, 173-subunit | Peptide | NA | ADH modification, 100mM MES at pH 6, 10ug/mL | (Udhayakumar et al., 2001) |  |
| PfAMA1.PL169 | Erythrocyte | Apical membrane antigen 1 , 169-subunit | Peptide | NA | ADH modification, 100mM MES at pH 6, 10ug/mL | (Udhayakumar et al., 2001) |  |
| HRP2 | Erythrocyte | Histidine rich protein 2, Type B | Recombinant, *E. coli* | GST | 50mM MES at pH 5, 20ug/mL | (Rogier et al., 2017) |  |
| Etramp 5 ag 1 | Erythrocyte | Early transcribed membrane protein 5, antigen 1 | Recombinant, *E. coli* | GST | 0.01M Na PBS at pH 7.2, 100ug/mL | (Wu et al., 2019;Achan et al., 2020) |  |
| HSP40 ag 1 | Erythrocyte | Heat shock protein 40 | Recombinant, *E. coli* | GST | 0.01M Na PBS at pH 7.2, 100ug/mL | (Wu et al., 2019) |  |
| H103/MSP11 | Erythrocyte | Merozoite surface protein 11 | Recombinant, *E. coli* | GST | 0.01M Na PBS at pH 7.2, 100ug/mL | (Pearce et al., 2005) |  |
| Rh2_2030 | Erythrocyte | Reticulocyte binding homologue 2 | Recombinant, *E. coli* | GST | 50mM MES at pH 5, 50ug/mL | (Richards et al., 2013;Achan et al., 2020) |  |
| EBA175 | Erythrocyte | Erythrocyte binding antigen 175, region III-V | Recombinant, *E. coli* | His tag | 0.01M Na PBS at pH 7.2, 200ug/mL | (Richards et al., 2013) |  |
| Etramp 4 ag 2 | Erythrocyte | Early transcribed membrane protein 4, antigen 2 | Recombinant, *E. coli* | GST | 0.01M Na PBS at pH 7.2, 115ug/mL | (Wu et al., 2019) |  |
| GEXP18 | Erythrocyte | Gametocyte-exported protein 18 | Recombinant, *E. coli* | GST | 0.01M Na PBS at pH 7.2, 200ug/mL | (Wu et al., 2019;Achan et al., 2020) |  |
| SEA-1 | Erythrocyte | Schizont egress antigen 1 | Recombinant, *E. coli* | GST | 50mM MES at pH 5, 20ug/mL | (Raj et al., 2014) |  |
| EBA181 | Erythrocyte | Erythrocyte binding antigen 181, region III-V | Recombinant, *E. coli* | GST | 0.01M Na PBS at pH 7.2, 100ug/mL | (Richards et al., 2013) |  |
| RH5.1 | Erythrocyte | Reticulocyte binding protein homologue 5 | Recombinant, *Drosophila* S2 cell | C-tag | 50mM MES at pH 5, 10ug/mL | (Jin et al., 2018) |  |
| SBP1 | Erythrocyte | Skeleton binding protein 1 | Recombinant, *E. coli* | GST | 50mM MES at pH 5, 15ug/mL | (Epp and Deitsch, 2006) |  |
| Hyp2 | Erythrocyte | Exported putative protein | Recombinant, *E. coli* | GST | 0.01M Na PBS at pH 7.2, 1000ug/mL | (Wu et al., 2019) |  |
| GLURP.R0 | Erythrocyte | Glutamate-rich protein R0 region | Peptide | NA | 50mM MES at pH 5, 30ug/mL | (Kerkhof et al., 2015) |  |
| GLURP.R2 | Erythrocyte | Glutamate-rich protein R2 region | Recombinant, *E. coli* | none | 0.01M Na PBS at pH 7.2, 15ug/mL | (Theisen et al., 1995;Wu et al., 2019;Achan et al., 2020) |  |
| GLURP.P3 | Erythrocyte | Glutamate-rich protein R0 P3 peptide | Peptide | NA | ADH modification, 100mM MES at pH 6, 10ug/mL | (Theisen et al., 2000) |  |
| EBA140 | Erythrocyte | Erythrocyte binding antigen 140, region III-V | Recombinant *Pichia pastoris* | His tag | 0.01M Na PBS at pH 7.2, 120ug/mL | (Richards et al., 2013) |  |
| Rh4.2 | Erythrocyte | Reticulocyte binding homologue 4 | *Drosophila* S2 cell line | GST | 50mM MES at pH 5, 20ug/mL | (Richards et al., 2013) |  |
| PfLDH | Erythrocyte | Lactate dehydrogenase | Recombinant, *E. coli* | GST | 50mM MES at pH 5, 20ug/mL | unpublished |  |
| PfAldolase | Erythrocyte | Aldolase enzyme | Recombinant, *E. coli* | His tag | 50mM MES at pH 5, 20ug/mL | unpublished |  |
| Pfs48/45 | Gametocyte | Gametocyte surface protein 48/45 | Peptide | NA | ADH modification, 100mM MES at pH 6, 10ug/mL | (Kumar et al., 1995) |  |
| Pfg.27 | Gametocyte | Gametocyte antigen 27 | Peptide | NA | ADH modification, 100mM MES at pH 6, 10ug/mL | (Ploton et al., 1995) |  |
| PvMSP1-19 | Erythrocyte | Merozoite surface protein 1, 19kD | Recombinant, *E. coli* | GST | 50mM MES at pH 5, 20ug/mL | (Priest et al., 2018) |  |
| PmMSP1-19 | Erythrocyte | Merozoite surface protein 1, 19kD | Recombinant, *E. coli* | GST | 50mM MES at pH 5, 20ug/mL | (Priest et al., 2018) |  |
| Tetanus toxoid | NA | Inactivated tetanus toxin | Commercial | - | 50mM MES at pH 5, 12.5ug/mL | (Scobie et al., 2016) |  |
| Sag2 | NA | Surface antigen glycoprotein 2A | Recombinant, *E. coli* | GST | 50mM MES at pH 5, 12.5ug/mL | (Priest et al., 2015) |  |
| GST | NA | Glutathione-*S*-transferase | Recombinant, *E. coli* | NA | 50mM MES at pH 5, 20ug/mL | (Moss et al., 2004) |  |
| * Not applicable | |  |  |  |  |  |  |
| ^†^ ADH modification: adipic dihydrazide incubation with beads to allow 10 atom spacer before peptide conjugation | | | | | | | |
| ^#^ MES, 2-(N-morpholinoethanesulfonic acid | | | | | | | |
|  |  |  |  |  |  |  |  |

Supplementary Table 1 References

Achan, J., Reuling, I.J., Yap, X.Z., Dabira, E., Ahmad, A., Cox, M., Nwakanma, D., Tetteh, K., Wu, L., Bastiaens, G.J.H., Abebe, Y., Manoj, A., Kaur, H., Miura, K., Long, C., Billingsley, P.F., Sim, B.K.L., Hoffman, S.L., Drakeley, C., Bousema, T., and D'alessandro, U. (2020). Serologic Markers of Previous Malaria Exposure and Functional Antibodies Inhibiting Parasite Growth Are Associated With Parasite Kinetics Following a Plasmodium falciparum Controlled Human Infection. *Clin Infect Dis* 70**,** 2544-2552.

Dutta, S., Lalitha, P.V., Ware, L.A., Barbosa, A., Moch, J.K., Vassell, M.A., Fileta, B.B., Kitov, S., Kolodny, N., Heppner, D.G., Haynes, J.D., and Lanar, D.E. (2002). Purification, characterization, and immunogenicity of the refolded ectodomain of the Plasmodium falciparum apical membrane antigen 1 expressed in Escherichia coli. *Infect Immun* 70**,** 3101-3110.

Epp, C., and Deitsch, K. (2006). Deciphering the export pathway of malaria surface proteins. *Trends Parasitol* 22**,** 401-404.

Fidock, D.A., Gras-Masse, H., Lepers, J.P., Brahimi, K., Benmohamed, L., Mellouk, S., Guerin-Marchand, C., Londono, A., Raharimalala, L., Meis, J.F., and Et Al. (1994). Plasmodium falciparum liver stage antigen-1 is well conserved and contains potent B and T cell determinants. *J Immunol* 153**,** 190-204.

Jin, J., Tarrant, R.D., Bolam, E.J., Angell-Manning, P., Soegaard, M., Pattinson, D.J., Dulal, P., Silk, S.E., Marshall, J.M., Dabbs, R.A., Nugent, F.L., Barrett, J.R., Hjerrild, K.A., Poulsen, L., Jorgensen, T., Brenner, T., Baleanu, I.N., Parracho, H.M., Tahiri-Alaoui, A., Whale, G., Moyle, S., Payne, R.O., Minassian, A.M., Higgins, M.K., Detmers, F.J., Lawrie, A.M., Douglas, A.D., Smith, R., De Jongh, W.A., Berrie, E., Ashfield, R., and Draper, S.J. (2018). Production, quality control, stability, and potency of cGMP-produced Plasmodium falciparum RH5.1 protein vaccine expressed in Drosophila S2 cells. *NPJ Vaccines* 3**,** 32.

Kerkhof, K., Canier, L., Kim, S., Heng, S., Sochantha, T., Sovannaroth, S., Vigan-Womas, I., Coosemans, M., Sluydts, V., and Ménard, D.J.M.J. (2015). Implementation and application of a multiplex assay to detect malaria-specific antibodies: a promising tool for assessing malaria transmission in Southeast Asian pre-elimination areas. 14**,** 338.

Kumar, N., Ploton, I., Koski, G., Ann-Lobo, C., and Contreras, C. (1995). Malaria transmission-blocking immunity. Identification of epitopes and evaluation of immunogenicity. *Adv Exp Med Biol* 383**,** 65-72.

Mahajan, B., Berzofsky, J.A., Boykins, R.A., Majam, V., Zheng, H., Chattopadhyay, R., De La Vega, P., Moch, J.K., Haynes, J.D., Belyakov, I.M., Nakhasi, H.L., and Kumar, S. (2010). Multiple antigen peptide vaccines against Plasmodium falciparum malaria. *Infect Immun* 78**,** 4613-4624.

Moss, D.M., Montgomery, J.M., Newland, S.V., Priest, J.W., and Lammie, P.J. (2004). Detection of cryptosporidium antibodies in sera and oral fluids using multiplex bead assay. *J Parasitol* 90**,** 397-404.

Osier, F.H., Fegan, G., Polley, S.D., Murungi, L., Verra, F., Tetteh, K.K., Lowe, B., Mwangi, T., Bull, P.C., Thomas, A.W., Cavanagh, D.R., Mcbride, J.S., Lanar, D.E., Mackinnon, M.J., Conway, D.J., and Marsh, K. (2008). Breadth and magnitude of antibody responses to multiple Plasmodium falciparum merozoite antigens are associated with protection from clinical malaria. *Infect Immun* 76**,** 2240-2248.

Pearce, J.A., Mills, K., Triglia, T., Cowman, A.F., and Anders, R.F. (2005). Characterisation of two novel proteins from the asexual stage of Plasmodium falciparum, H101 and H103. *Mol Biochem Parasitol* 139**,** 141-151.

Ploton, I.N., Wizel, B., Viscidi, R., and Kumar, N. (1995). Mapping of two overlapping linear epitopes in Pfg27 recognized by Plasmodium falciparum transmission-blocking monoclonal antibodies. *Vaccine* 13**,** 1161-1169.

Priest, J.W., Moss, D.M., Arnold, B.F., Hamlin, K., Jones, C.C., and Lammie, P.J. (2015). Seroepidemiology of Toxoplasma in a coastal region of Haiti: multiplex bead assay detection of immunoglobulin G antibodies that recognize the SAG2A antigen. *Epidemiol Infect* 143**,** 618-630.

Priest, J.W., Plucinski, M.M., Huber, C.S., Rogier, E., Mao, B., Gregory, C.J., Candrinho, B., Colborn, J., and Barnwell, J.W. (2018). Specificity of the IgG antibody response to Plasmodium falciparum, Plasmodium vivax, Plasmodium malariae, and Plasmodium ovale MSP1 19 subunit proteins in multiplexed serologic assays. *Malar J* 17**,** 417.

Raj, D.K., Nixon, C.P., Nixon, C.E., Dvorin, J.D., Dipetrillo, C.G., Pond-Tor, S., Wu, H.W., Jolly, G., Pischel, L., Lu, A., Michelow, I.C., Cheng, L., Conteh, S., Mcdonald, E.A., Absalon, S., Holte, S.E., Friedman, J.F., Fried, M., Duffy, P.E., and Kurtis, J.D. (2014). Antibodies to PfSEA-1 block parasite egress from RBCs and protect against malaria infection. *Science* 344**,** 871-877.

Richards, J.S., Arumugam, T.U., Reiling, L., Healer, J., Hodder, A.N., Fowkes, F.J., Cross, N., Langer, C., Takeo, S., Uboldi, A.D., Thompson, J.K., Gilson, P.R., Coppel, R.L., Siba, P.M., King, C.L., Torii, M., Chitnis, C.E., Narum, D.L., Mueller, I., Crabb, B.S., Cowman, A.F., Tsuboi, T., and Beeson, J.G. (2013). Identification and prioritization of merozoite antigens as targets of protective human immunity to Plasmodium falciparum malaria for vaccine and biomarker development. *J Immunol* 191**,** 795-809.

Rogier, E., Plucinski, M., Lucchi, N., Mace, K., Chang, M., Lemoine, J.F., Candrinho, B., Colborn, J., Dimbu, R., Fortes, F., Udhayakumar, V., and Barnwell, J. (2017). Bead-based immunoassay allows sub-picogram detection of histidine-rich protein 2 from Plasmodium falciparum and estimates reliability of malaria rapid diagnostic tests. *PLoS One* 12**,** e0172139.

Rogier, E., Wiegand, R., Moss, D., Priest, J., Angov, E., Dutta, S., Journel, I., Jean, S.E., Mace, K., Chang, M., Lemoine, J.F., Udhayakumar, V., and Barnwell, J.W. (2015). Multiple comparisons analysis of serological data from an area of low Plasmodium falciparum transmission. *Malar J* 14**,** 436.

Scobie, H.M., Mao, B., Buth, S., Wannemuehler, K.A., Sorensen, C., Kannarath, C., Jenks, M.H., Moss, D.M., Priest, J.W., Soeung, S.C., Deming, M.S., Lammie, P.J., and Gregory, C.J. (2016). Tetanus Immunity among Women Aged 15 to 39 Years in Cambodia: a National Population-Based Serosurvey, 2012. *Clin Vaccine Immunol* 23**,** 546-554.

Theisen, M., Soe, S., Jessing, S.G., Okkels, L.M., Danielsen, S., Oeuvray, C., Druilhe, P., and Jepsen, S. (2000). Identification of a major B-cell epitope of the Plasmodium falciparum glutamate-rich protein (GLURP), targeted by human antibodies mediating parasite killing. *Vaccine* 19**,** 204-212.

Theisen, M., Vuust, J., Gottschau, A., Jepsen, S., and Hogh, B. (1995). Antigenicity and immunogenicity of recombinant glutamate-rich protein of Plasmodium falciparum expressed in Escherichia coli. *Clin Diagn Lab Immunol* 2**,** 30-34.

Udhayakumar, V., Kariuki, S., Kolczack, M., Girma, M., Roberts, J.M., Oloo, A.J., Nahlen, B.L., and Lal, A.A. (2001). Longitudinal study of natural immune responses to the Plasmodium falciparum apical membrane antigen (AMA-1) in a holoendemic region of malaria in western Kenya: Asembo Bay Cohort Project VIII. *Am J Trop Med Hyg* 65**,** 100-107.

Wu, L., Hall, T., Ssewanyana, I., Oulton, T., Patterson, C., Vasileva, H., Singh, S., Affara, M., Mwesigwa, J., Correa, S., Bah, M., D'alessandro, U., Sepulveda, N., Drakeley, C., and Tetteh, K.K.A. (2019). Optimisation and standardisation of a multiplex immunoassay of diverse Plasmodium falciparum antigens to assess changes in malaria transmission using sero-epidemiology. *Wellcome Open Res* 4**,** 26.

Yang, C., Shi, Y.P., Udhayakumar, V., Alpers, M.P., Povoa, M.M., Hawley, W.A., Collins, W.E., and Lal, A.A. (1995). Sequence variations in the non-repetitive regions of the liver stage-specific antigen-1 (LSA-1) of Plasmodium falciparum from field isolates. *Mol Biochem Parasitol* 71**,** 291-294.

| **Supplementary Table 2.** Characteristics of participants assayed for antibody dynamics following antimalarial treatment | |
| --- | --- |
| Number of participants | 104 |
| Length of follow-up (days), median (range) | 42 (20-42) |
| Age (years), median (range) | 3 (0.5-11) |
| Parasitemia at Day 0 (parasites/µL), median (range) | 22,065 (3,916-200,000) |
| Female, % | 47% |
| Slide negative on Day 3, % | 100% |
| No recurrent parasitemia during follow-up, % | 89% |

**Supplementary Figure 1.** Number of samples analyzed at each day of follow up.

**Supplementary Figure 2.** Antibody dynamic plots for all antigens in the multiplex panel. Line on each plot displays local regression (LOESS) fitting for assay signal among different persons’ responses.

(Included as separate .pdf file)

**Supplementary Figure 3.** Distribution of acquisition and decay parameters for IgM to different antigens in Angolan children treated for malaria. Antigens are color-coded by category: sporozoite (white), hepatic (pink), erythrocytic (blue), gametocyte (green), non-falciparum *Plasmodium* (violet) and control (yellow). Boxplots display median as black solid line with box length representing interquartile range (IQR) and whiskers 1.5x IQR. Outliers above or below 1.5x IQR represented as circles. MFI-bg: median fluorescence intensity-background.


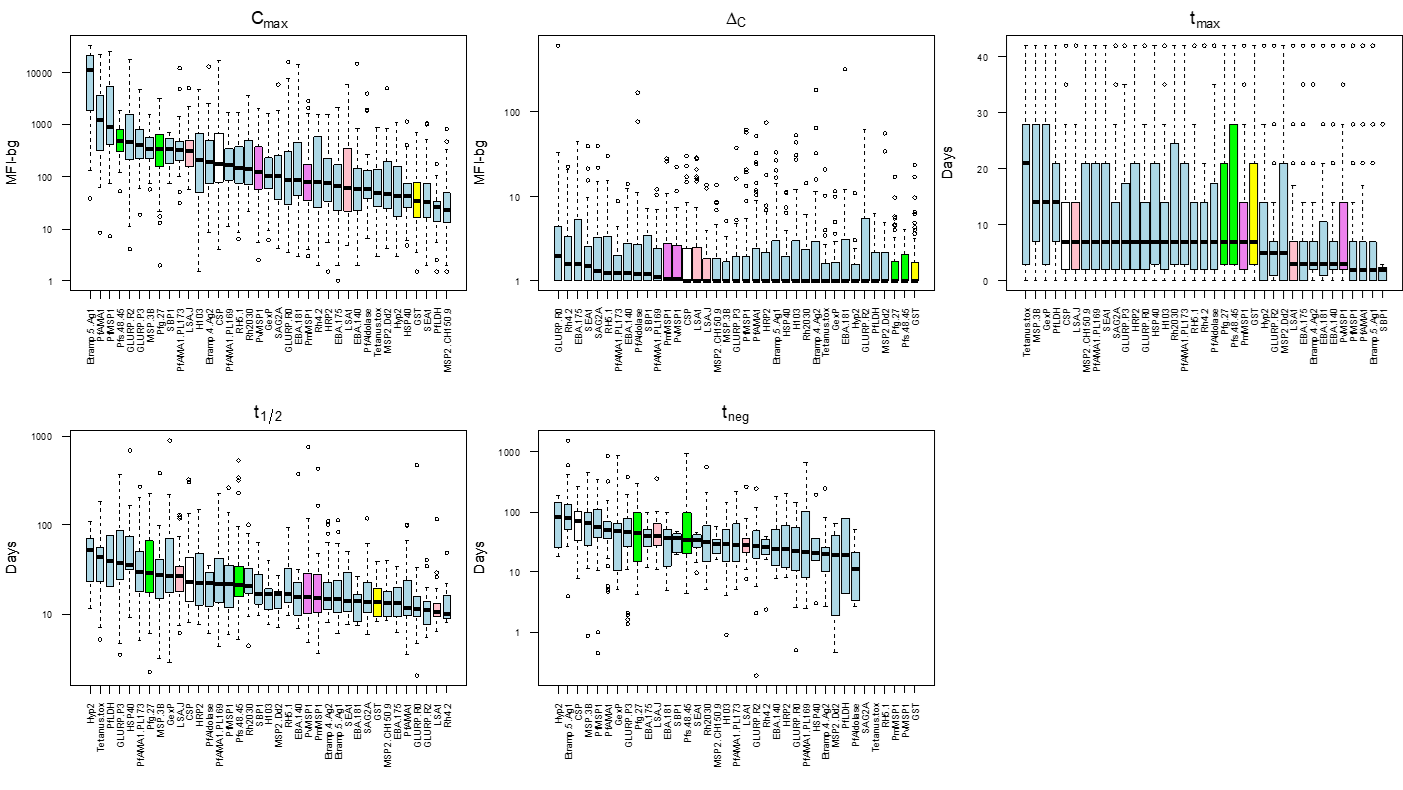


**Supplementary Figure 4.** Distribution of acquisition and decay parameters for IgA to different antigens in Angolan children treated for malaria. Antigens are color-coded by category: sporozoite (white), hepatic (pink), erythrocytic (blue), gametocyte (green), non-falciparum *Plasmodium* (violet) and control (yellow). Boxplots display median as black solid line with box length representing interquartile range (IQR) and whiskers 1.5x IQR. Outliers above or below 1.5x IQR represented as circles. MFI-bg: median fluorescence intensity-background.


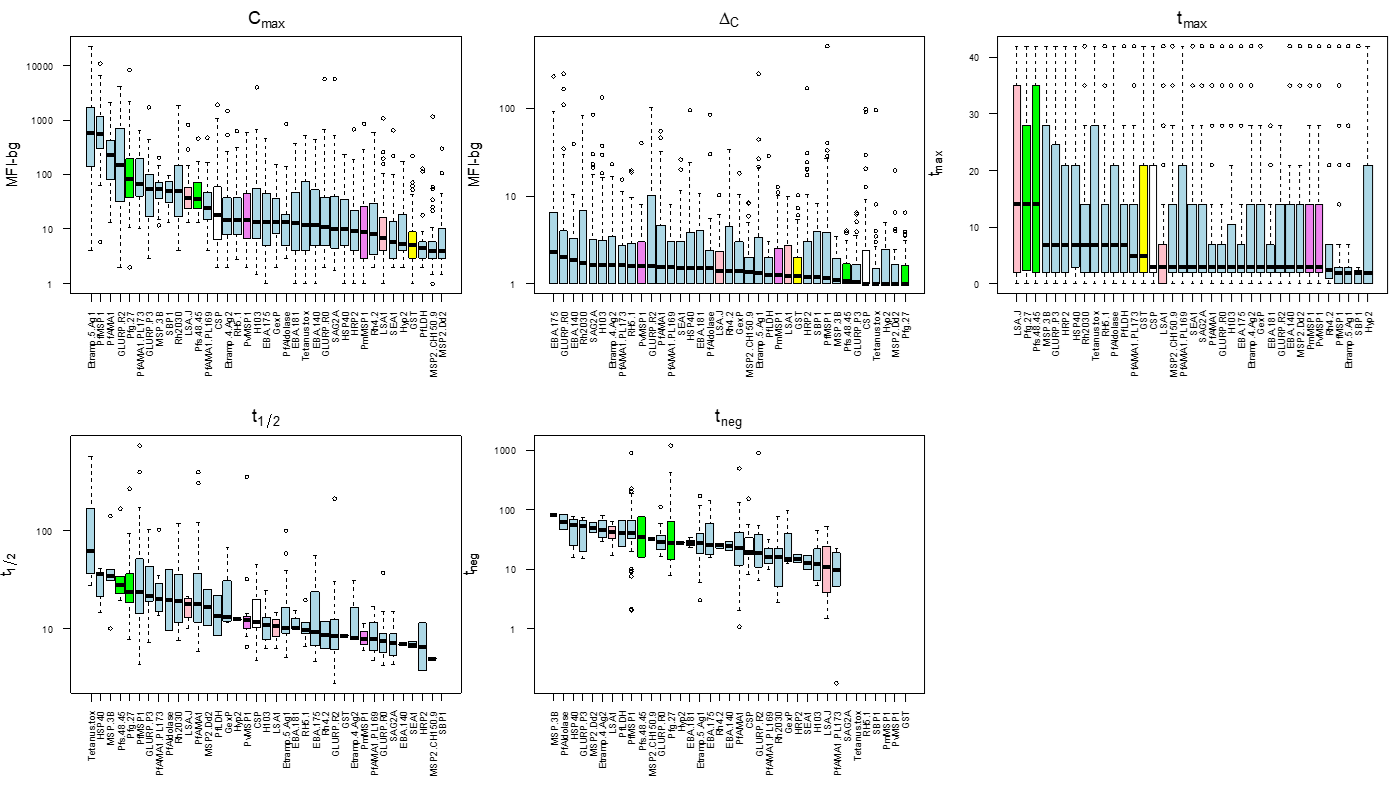


**Supplementary Figure 5.** Distribution of acquisition and decay parameters for IgG_3_ to different antigens in Angolan children treated for malaria. Antigens are color-coded by category: sporozoite (white), hepatic (pink), erythrocytic (blue), gametocyte (green), non-falciparum *Plasmodium* (violet) and control (yellow). Boxplots display median as black solid line with box length representing interquartile range (IQR) and whiskers 1.5x IQR. Outliers above or below 1.5x IQR represented as circles. MFI-bg: median fluorescence intensity-background.


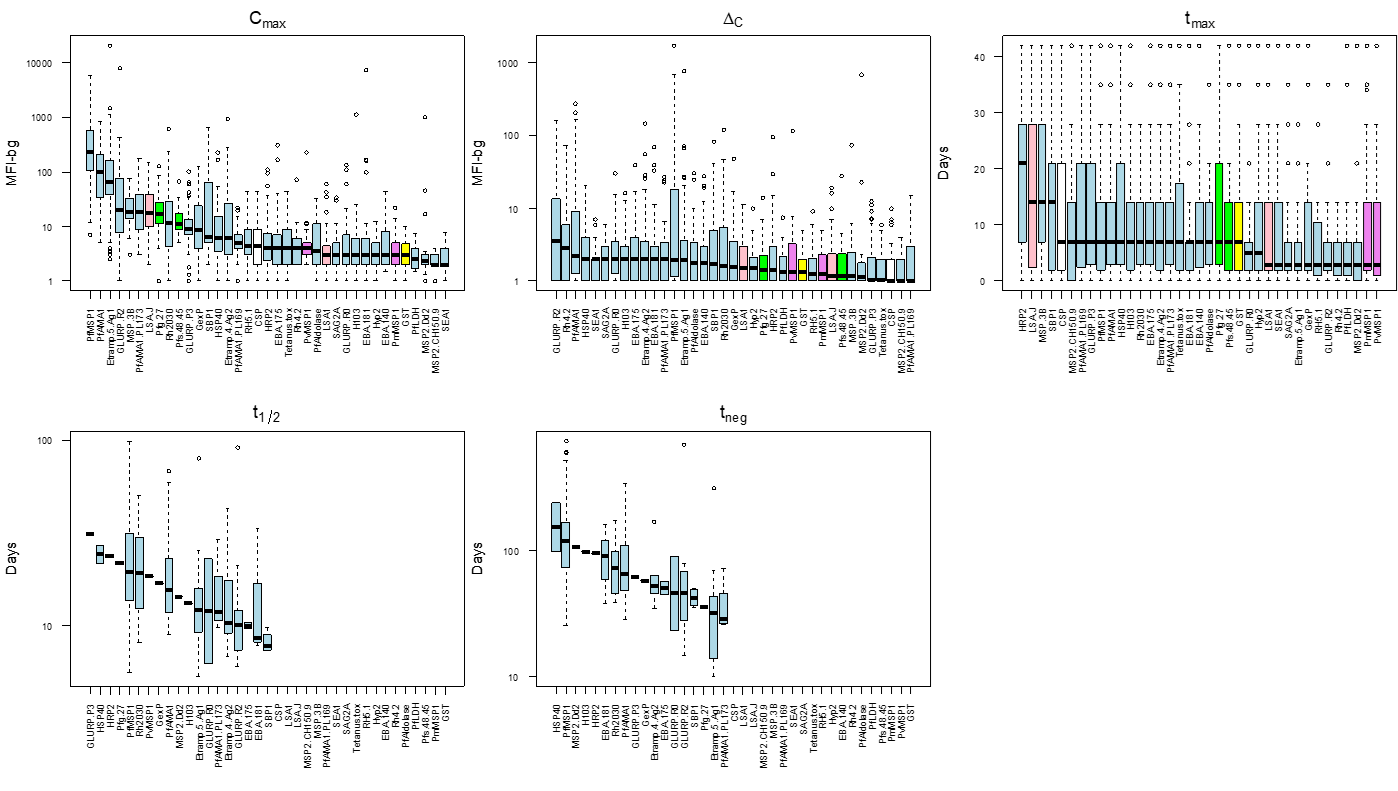


**Supplementary Figure 6.** Examples of Ig dynamics for one sporozoite (CSP), one liver-stage (LSA1), two blood stage (PfMSP1-19 and Etramp 5 ag 1) and one gametocyte (Pfs 48/45) antigens across sampling follow-up visits. Three childrens’ responses were selected to represent: blood stage antigens with delayed IgG_1_ t_max_ (Person 1), early t_max_ for all antigens and isotypes (Person 2), and notable IgG_3_ response and long t_neg_ for blood stage antigens (Person 3).


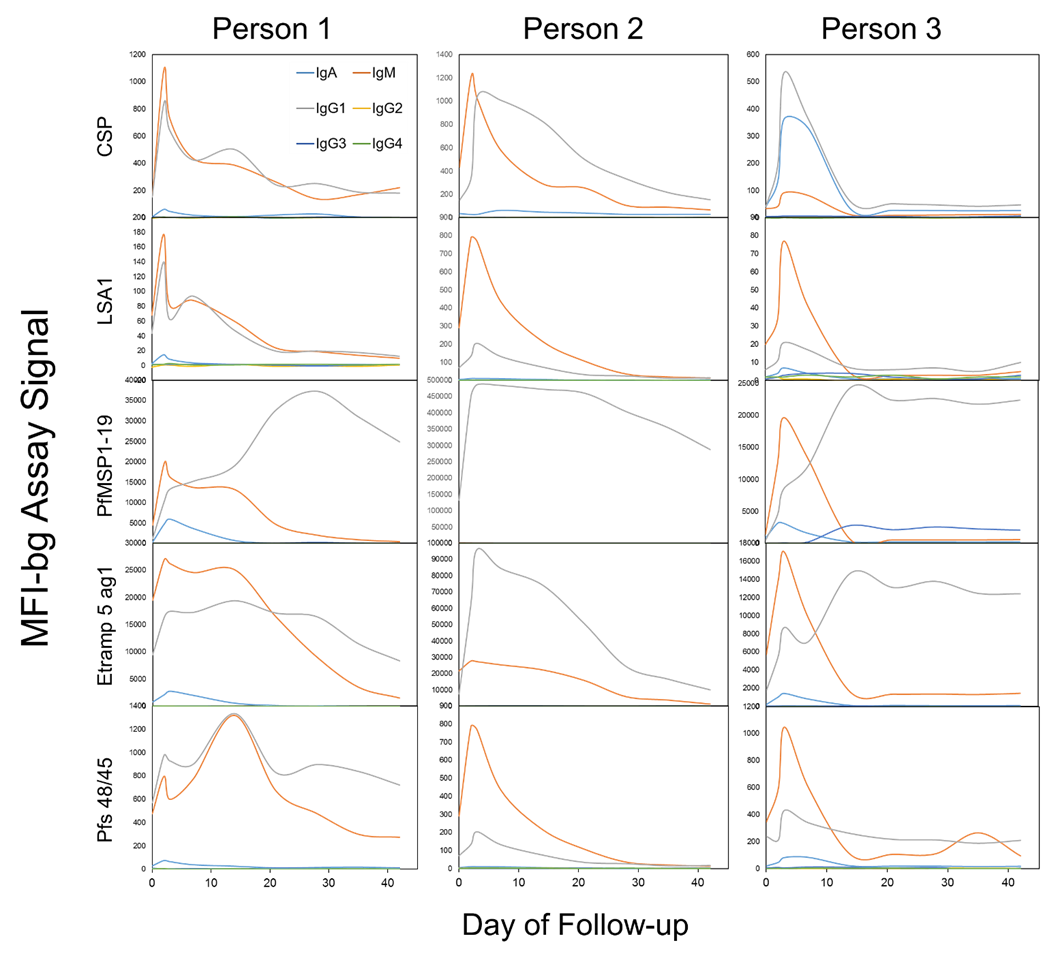

Supplement: Supplementary file 1 [file Data_Sheet_1.DOCX]
