# Supplementary material for: Framework for Characterizing Longitudinal Antibody Response in Children After Plasmodium falciparum Infection
Source: Front Immunol. 2021 Mar 2;12:617951. doi: 10.3389/fimmu.2021.617951 (PMC7960919; doi:10.3389/fimmu.2021.617951)
Supplement: Supplementary file 2 [file Data_Sheet_2.PDF]

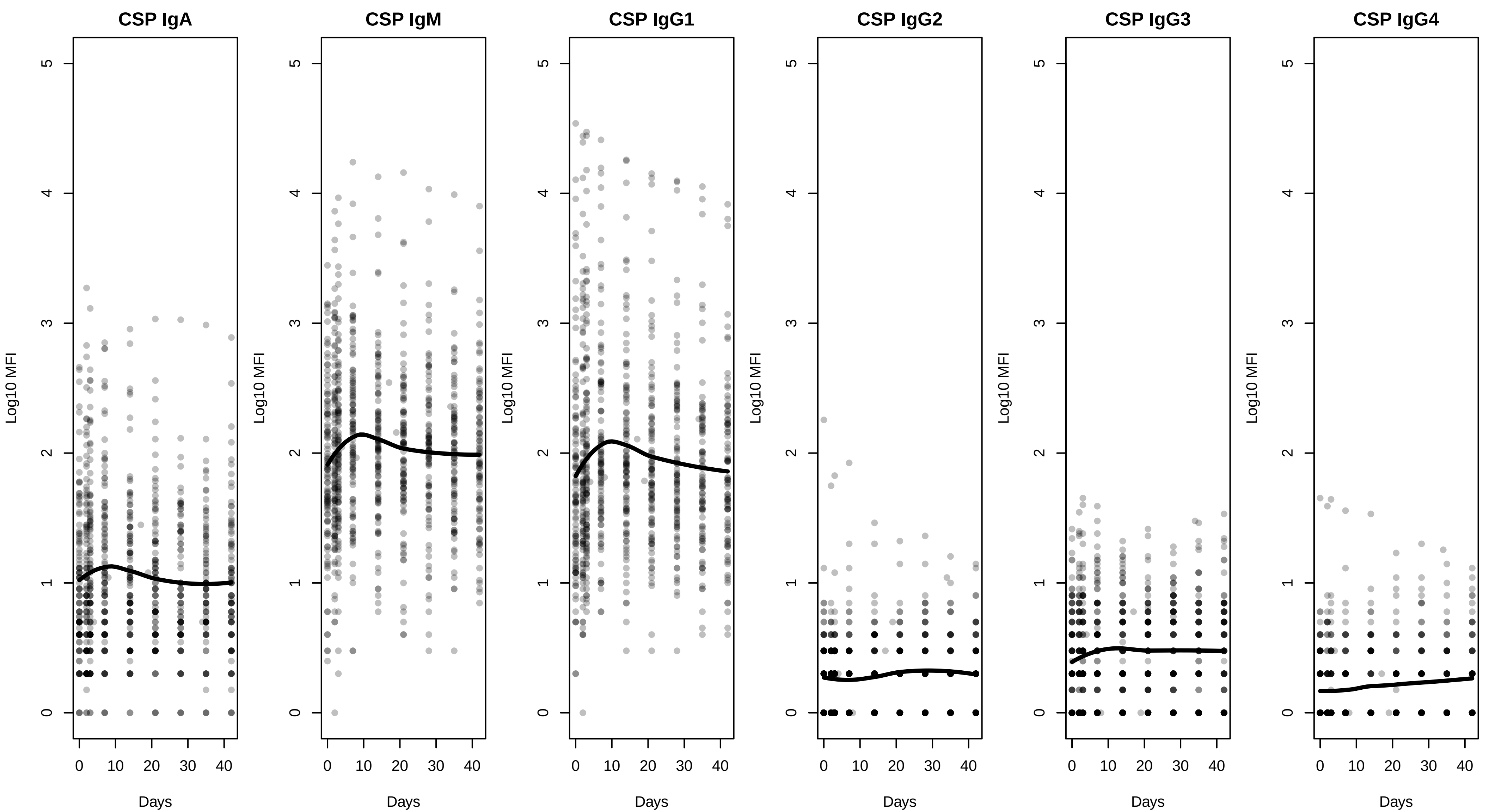
CSP IgA
CSP IgM
CSP IgG1
CSP IgG2
CSP IgG3
CSP IgG4
Log10 MFI
Days
0
1
2
3
4
5
0
10
20
30
40

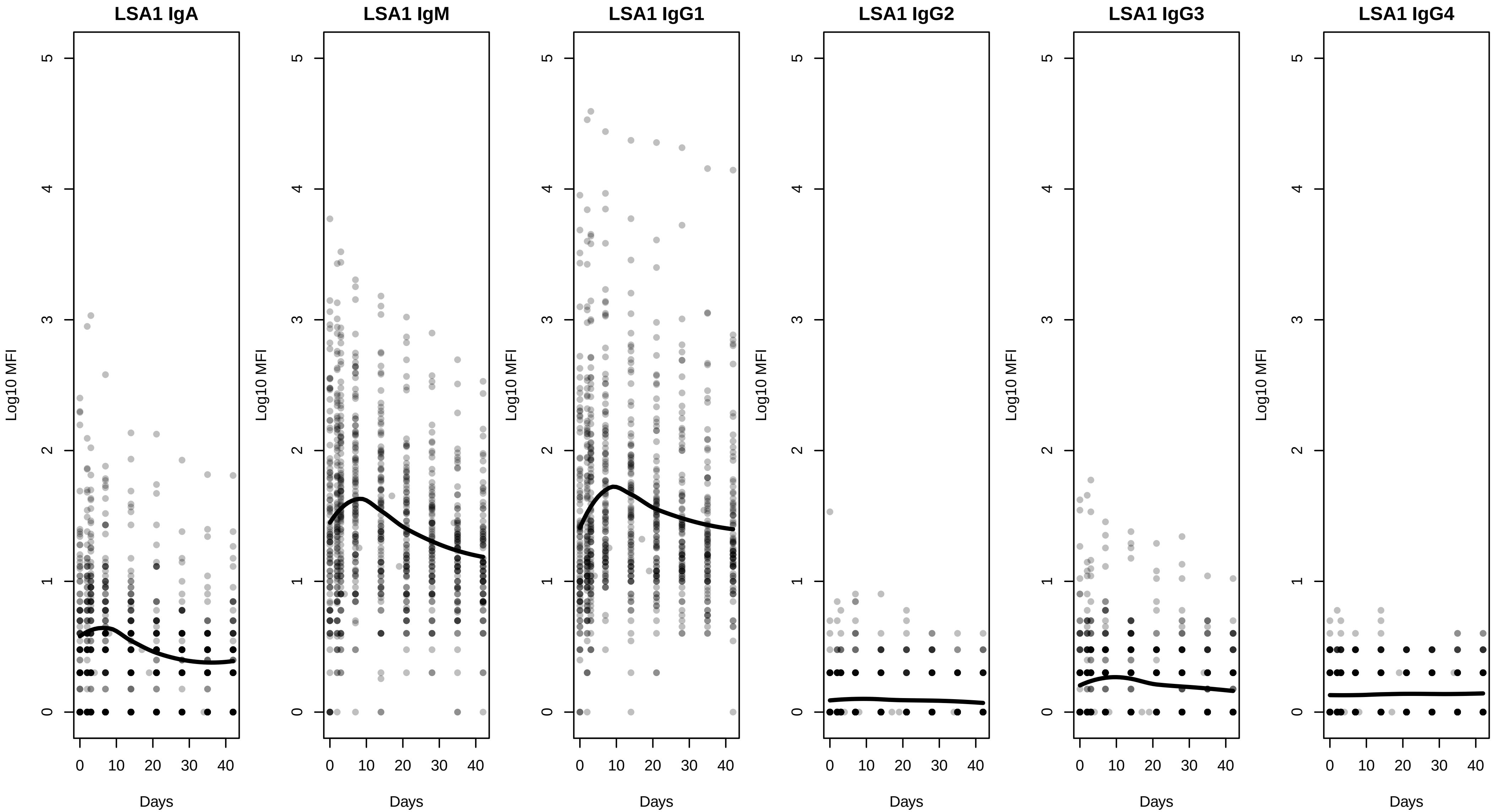

LSA1 IgA
LSA1 IgM
LSA1 IgG1
LSA1 IgG2
LSA1 IgG3
LSA1 IgG4
Log10 MFI
Days
0
1
2
3
4
5
0
10
20
30
40

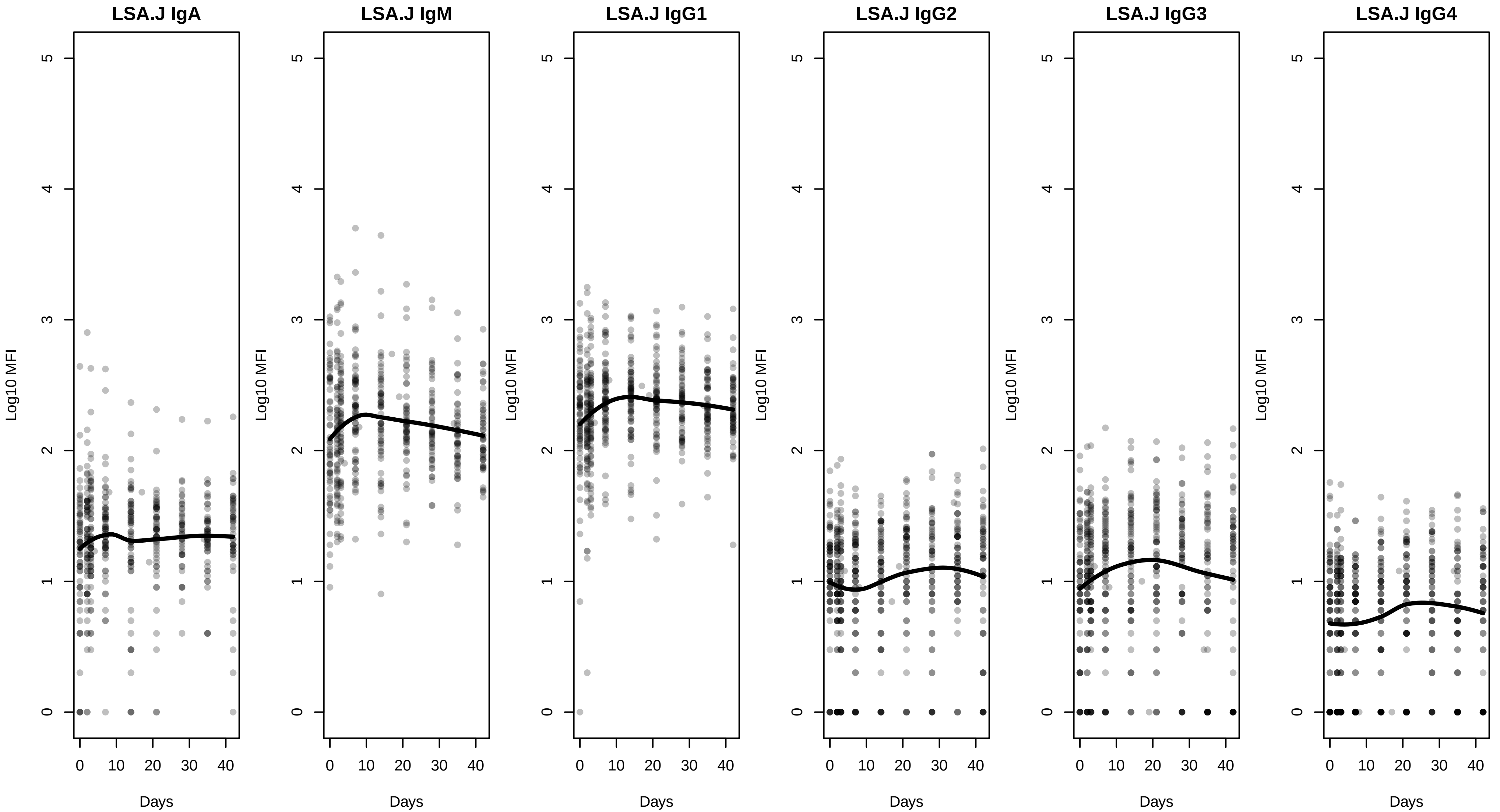

LSA.J IgA
LSA.J IgM
LSA.J IgG1
LSA.J IgG2
LSA.J IgG3
LSA.J IgG4
Log10 MFI
Days
0
1
2
3
4
5
0
10
20
30
40

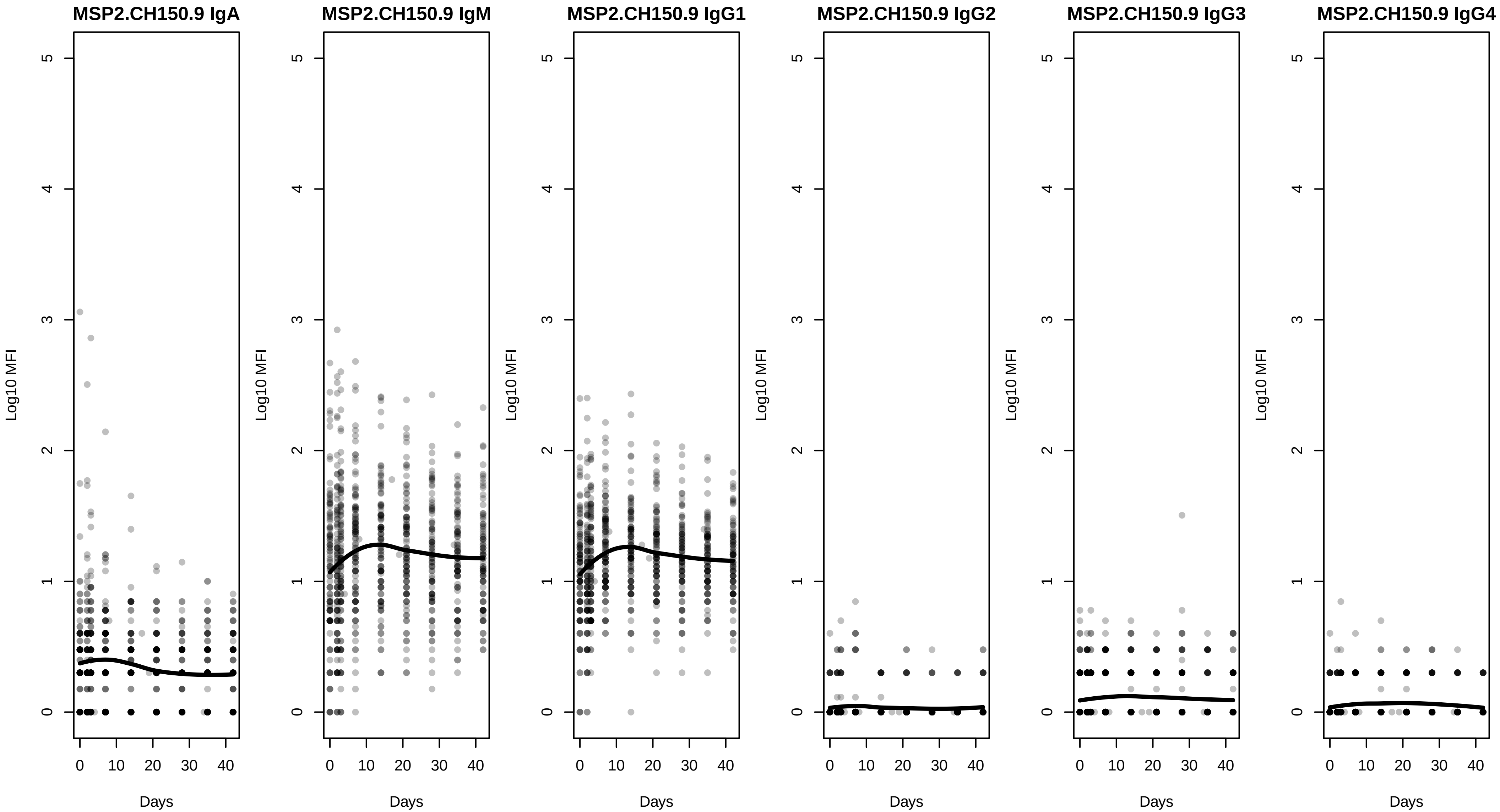
MSP2.CH150.9 IgA
MSP2.CH150.9 IgM
MSP2.CH150.9 IgG1
MSP2.CH150.9 IgG2
MSP2.CH150.9 IgG3
MSP2.CH150.9 IgG4
Log10 MFI
Days
0
1
2
3
4
5
0
10
20
30
40

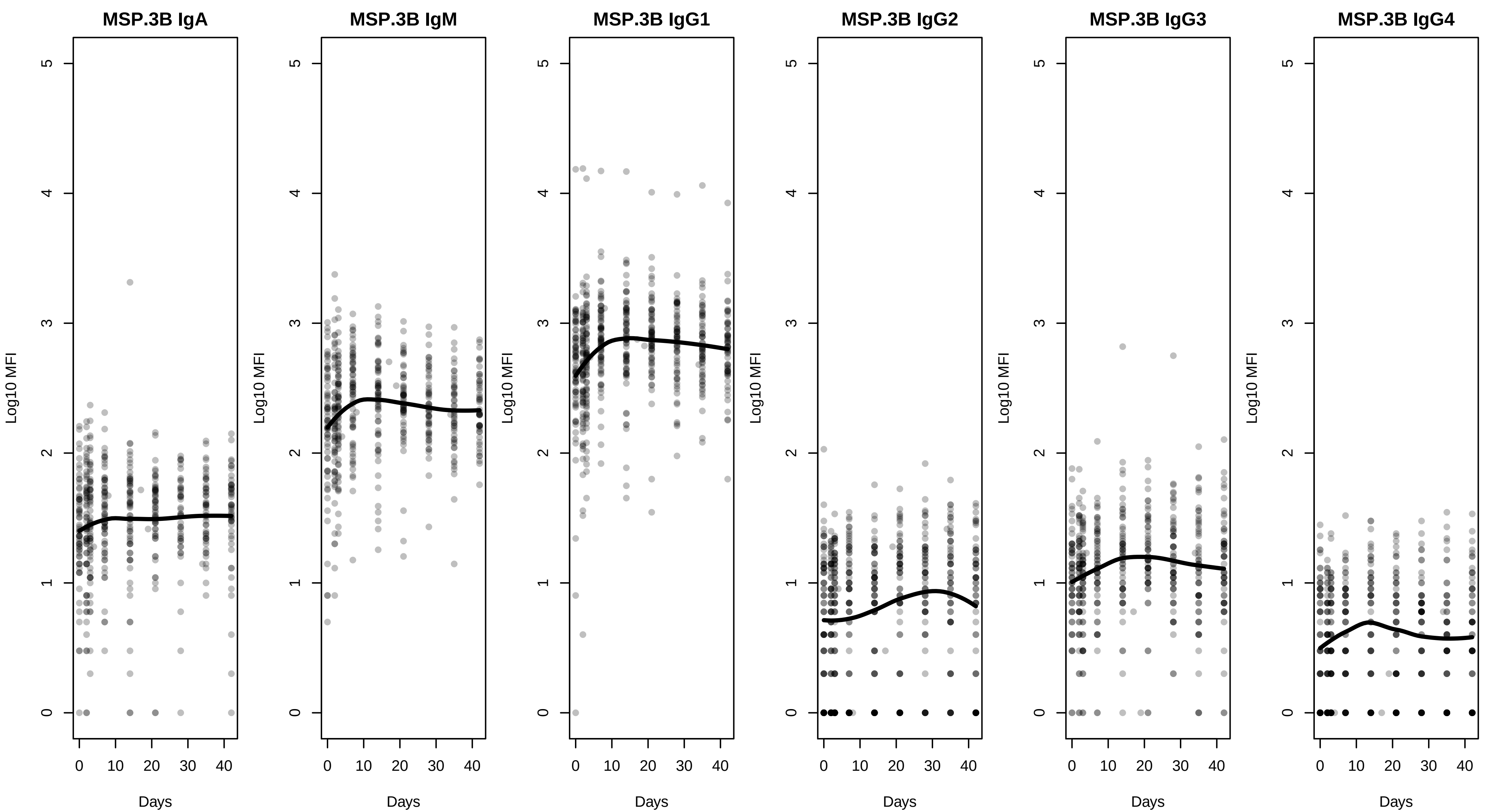
MSP.3B IgA
MSP.3B IgM
MSP.3B IgG1
MSP.3B IgG2
MSP.3B IgG3
MSP.3B IgG4
Log10 MFI
Days
0
1
2
3
4
5
0
10
20
30
40

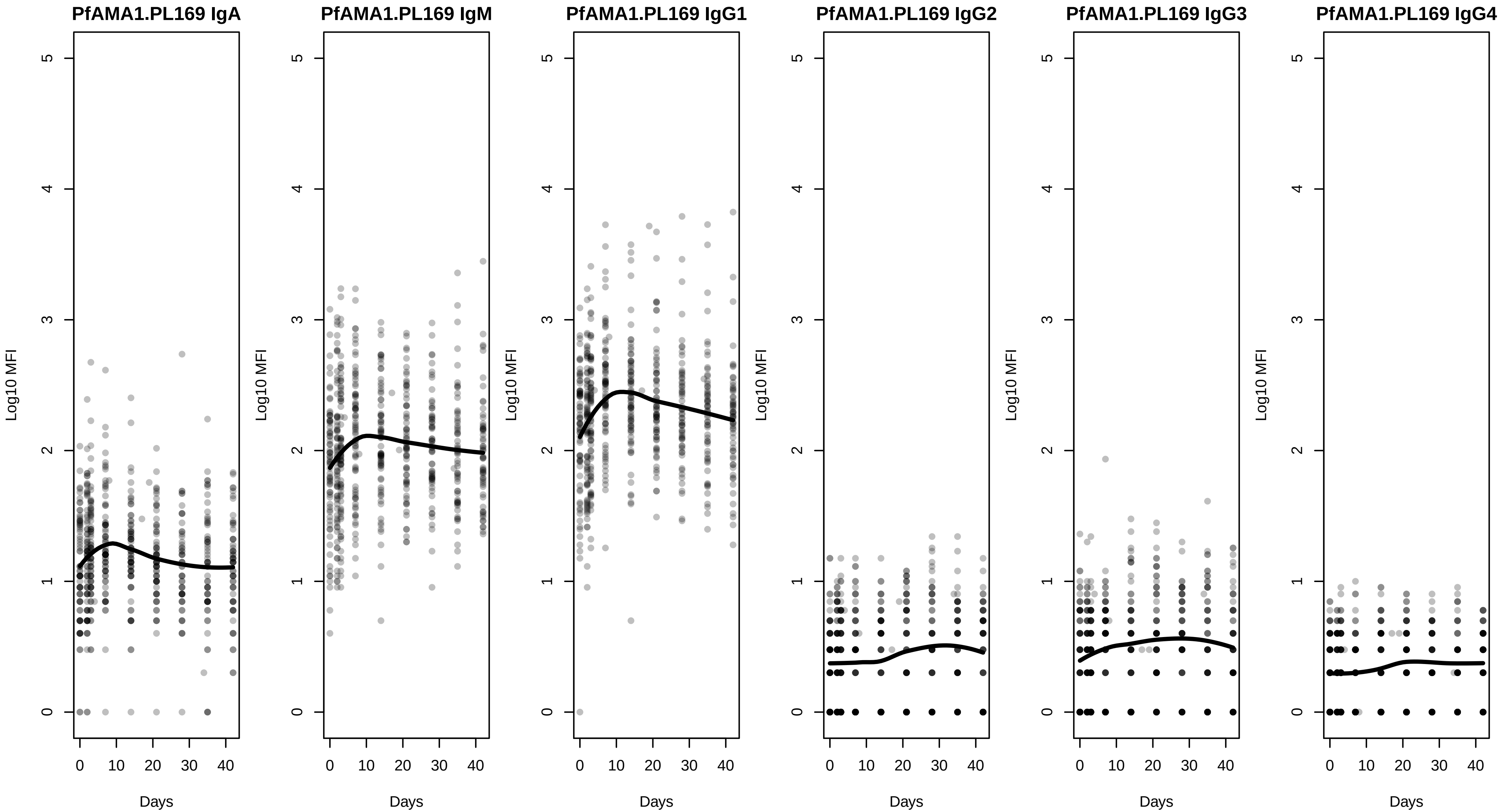
PfAMA1.PL169 IgA
PfAMA1.PL169 IgM
PfAMA1.PL169 IgG1
PfAMA1.PL169 IgG2
PfAMA1.PL169 IgG3
PfAMA1.PL169 IgG4
Log10 MFI
Days
0
1
2
3
4
5
10
20
30
40

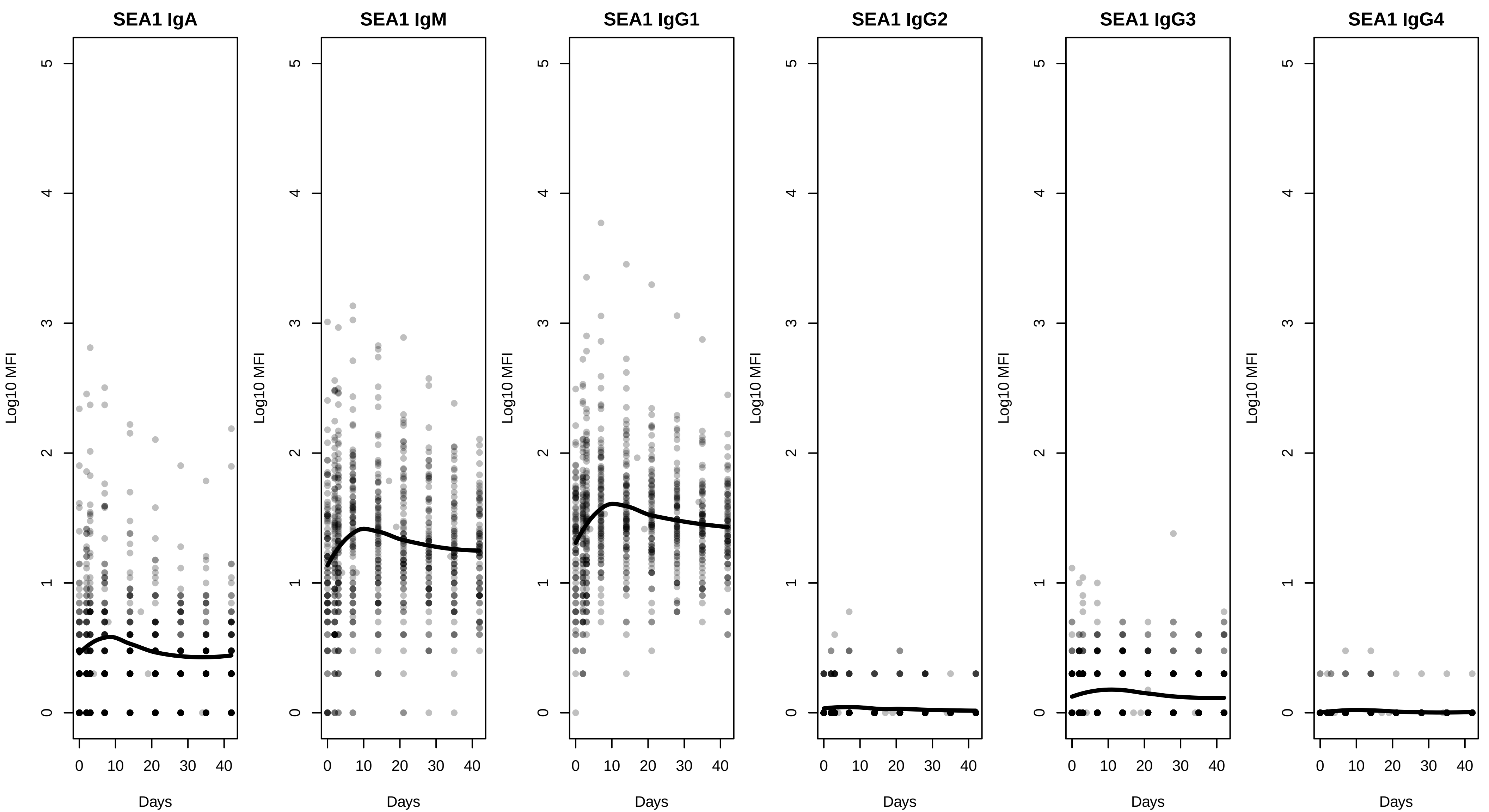
SEA1 IgA
SEA1 IgM
SEA1 IgG1
SEA1 IgG2
SEA1 IgG3
SEA1 IgG4
Log10 MFI
Days
0
1
2
3
4
5
10
20
30
40

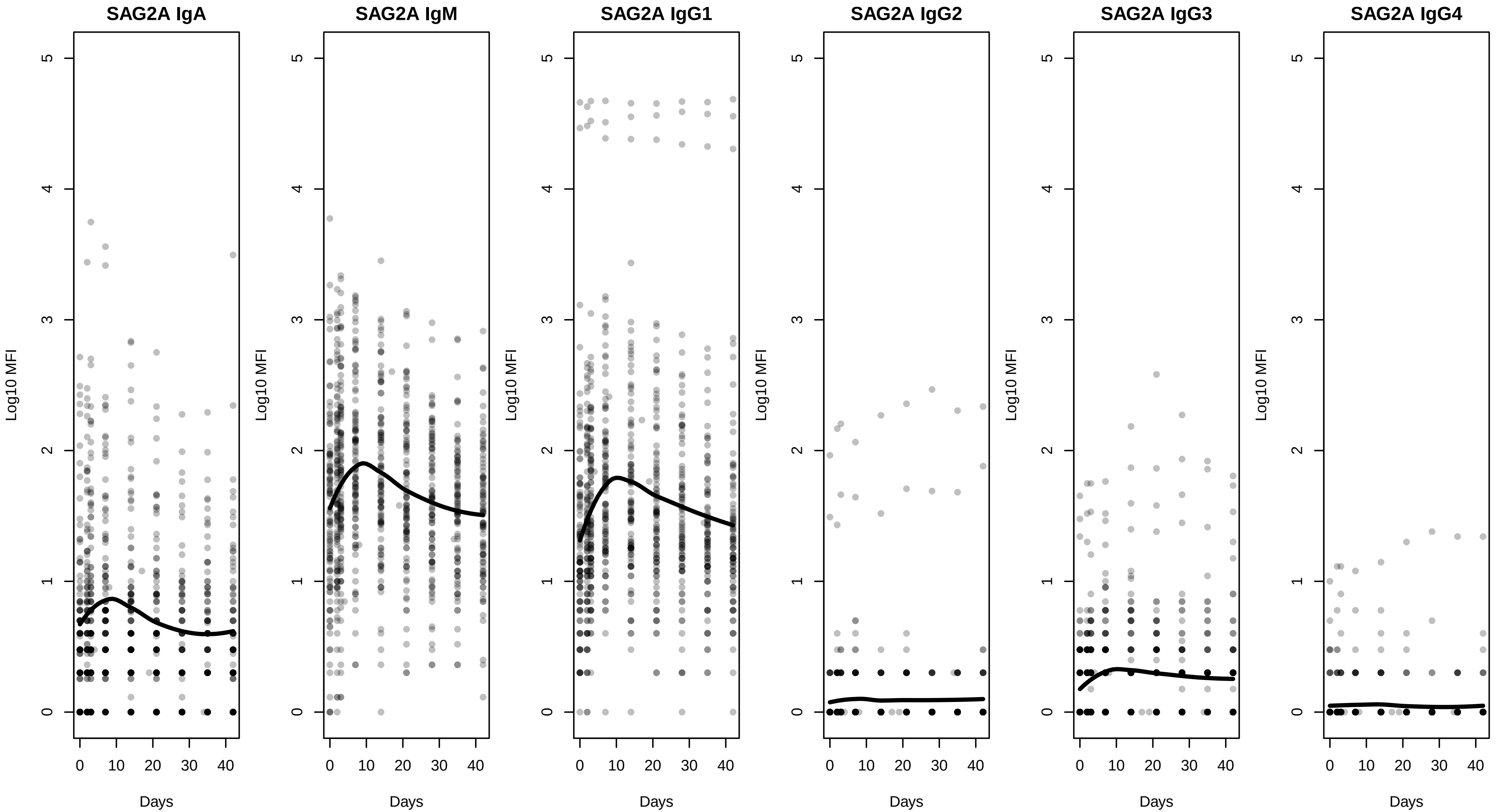

SAG2A IgA
SAG2A IgM
SAG2A IgG1
SAG2A IgG2
SAG2A IgG3
SAG2A IgG4
Log10 MFI
Days
0
1
2
3
4
5
0 10 20 30 40

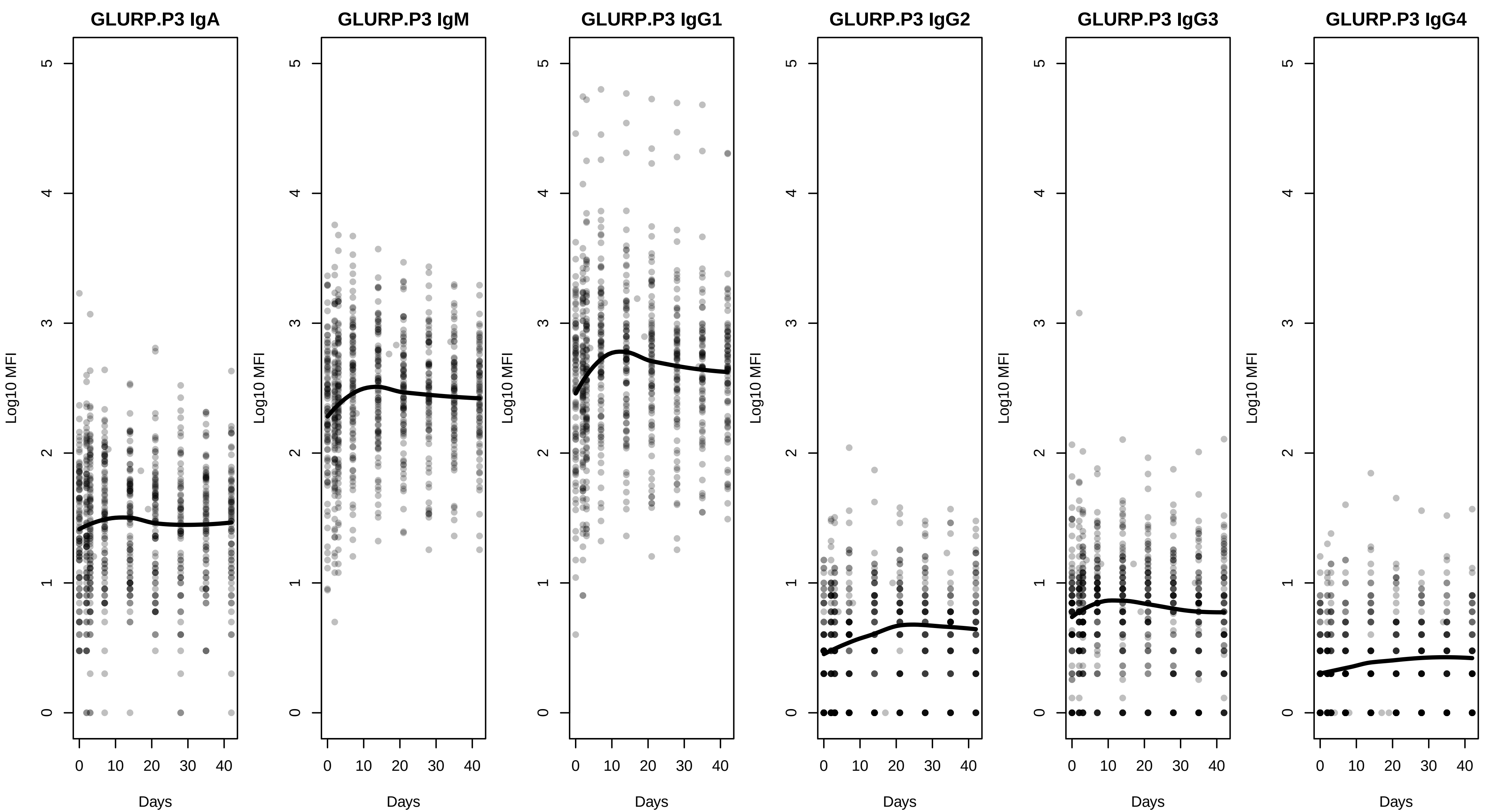
GLURP.P3 IgA
GLURP.P3 IgM
GLURP.P3 IgG1
GLURP.P3 IgG2
GLURP.P3 IgG3
GLURP.P3 IgG4
Log10 MFI
Days
0
1
2
3
4
5
0
10
20
30
40

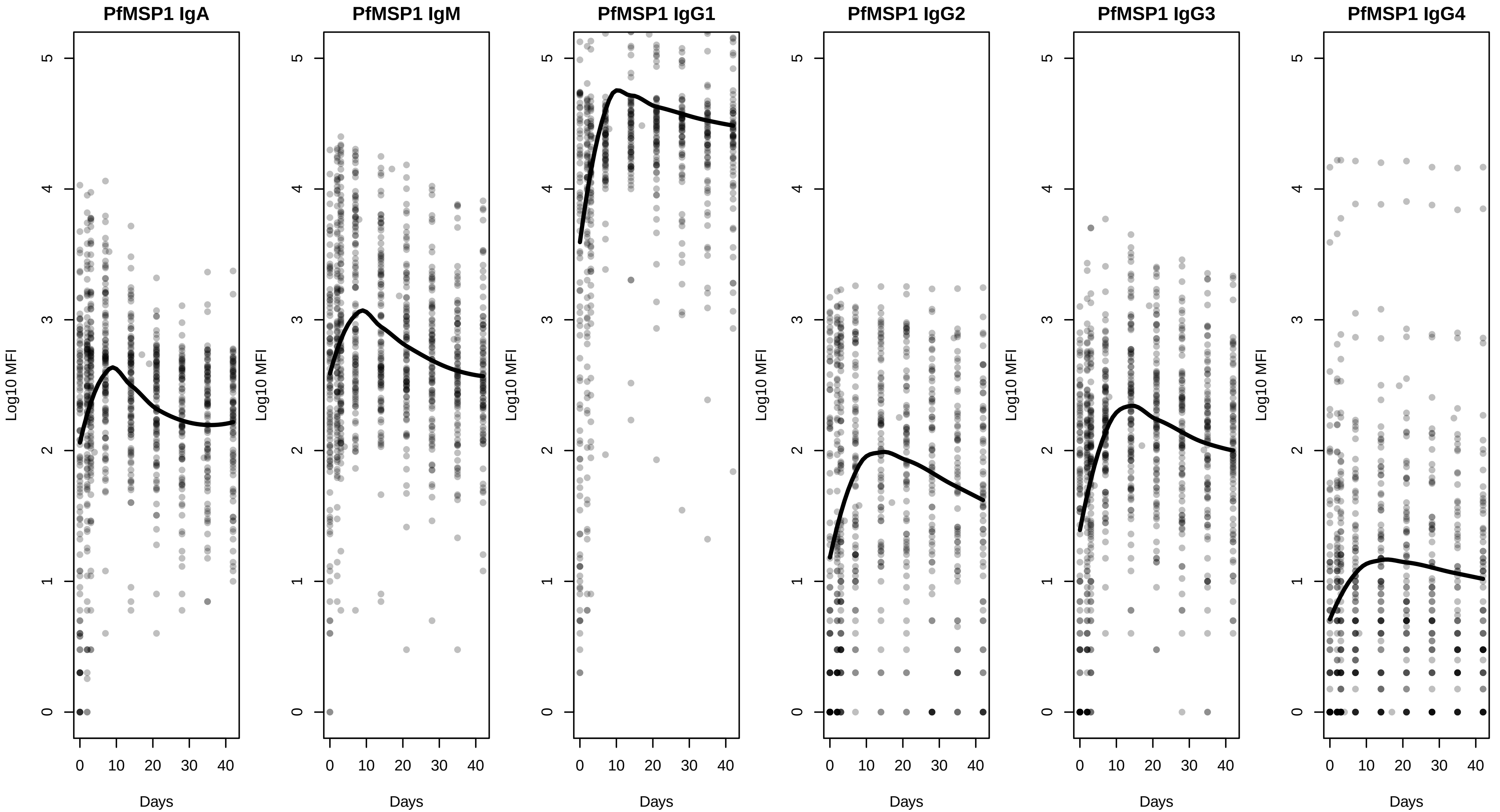
PfMSP1 IgA
PfMSP1 IgM
PfMSP1 IgG1
PfMSP1 IgG2
PfMSP1 IgG3
PfMSP1 IgG4
Log10 MFI
Days
0
1
2
3
4
5
0
10
20
30
40

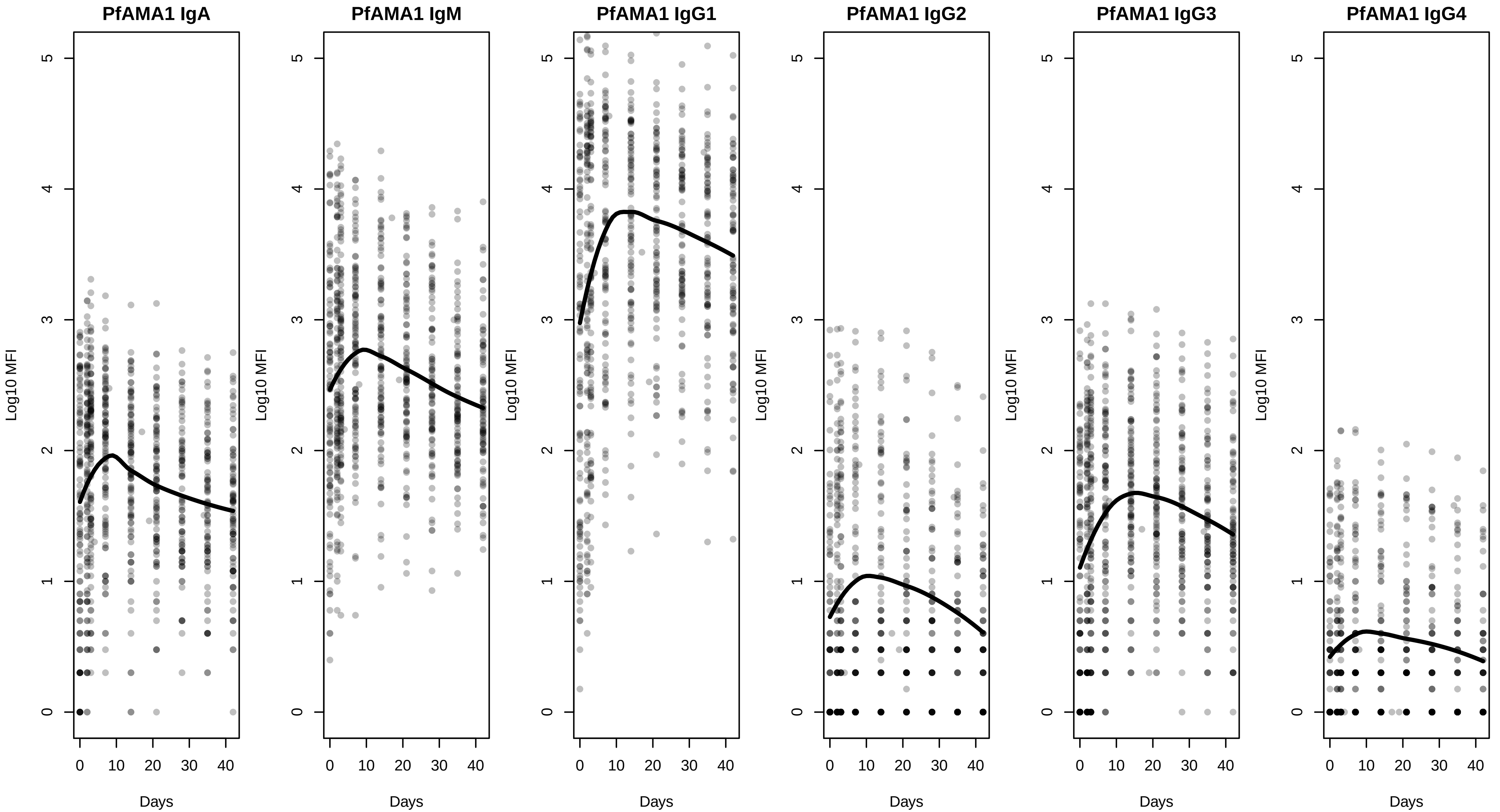

PfAMA1 IgA
PfAMA1 IgM
PfAMA1 IgG1
PfAMA1 IgG2
PfAMA1 IgG3
PfAMA1 IgG4
Log10 MFI
Days
0
1
2
3
4
5
0
10
20
30
40

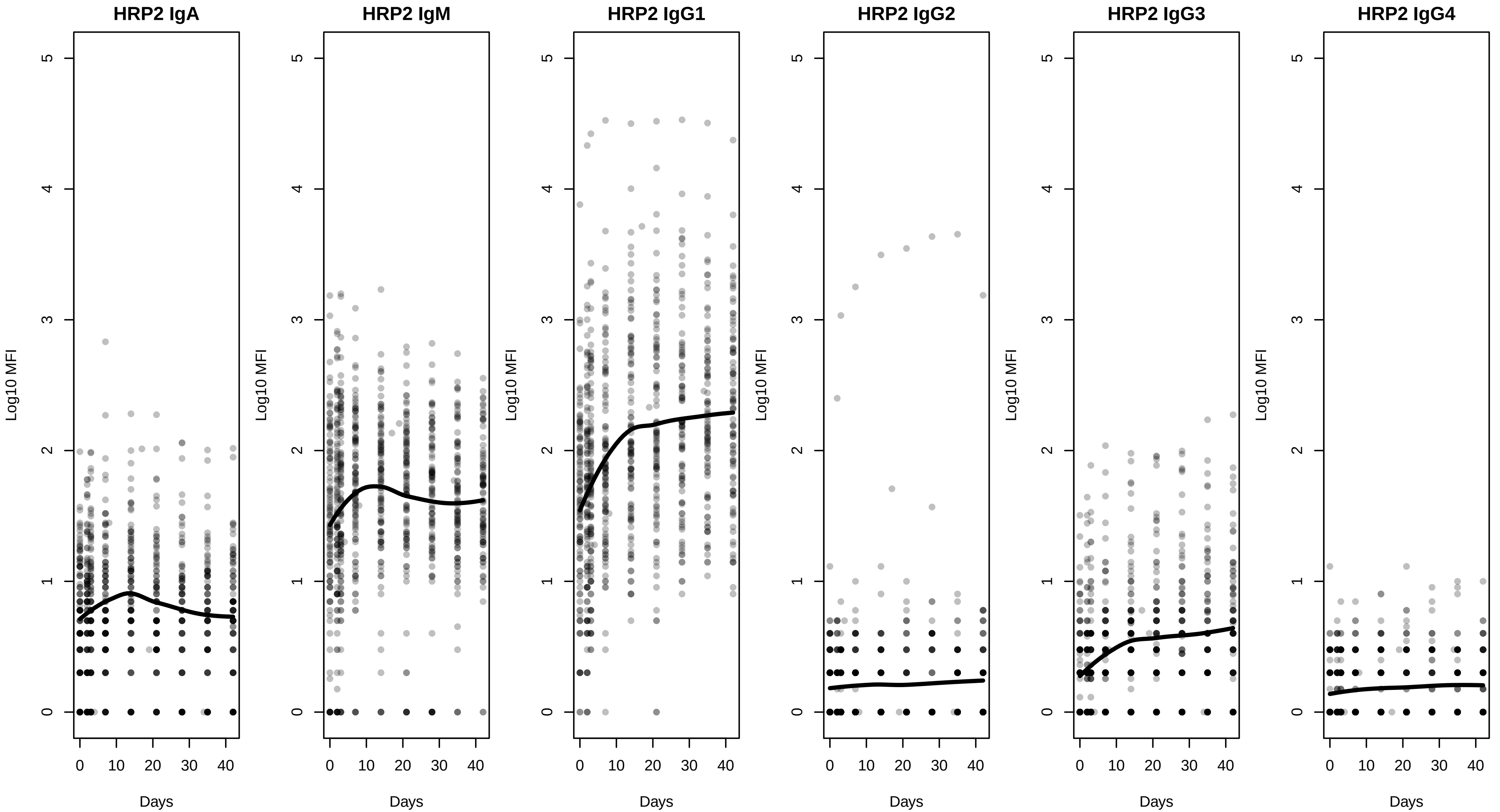
HRP2 IgA
HRP2 IgM
HRP2 IgG1
HRP2 IgG2
HRP2 IgG3
HRP2 IgG4
Log10 MFI
Days
0
1
2
3
4
5
0
10
20
30
40

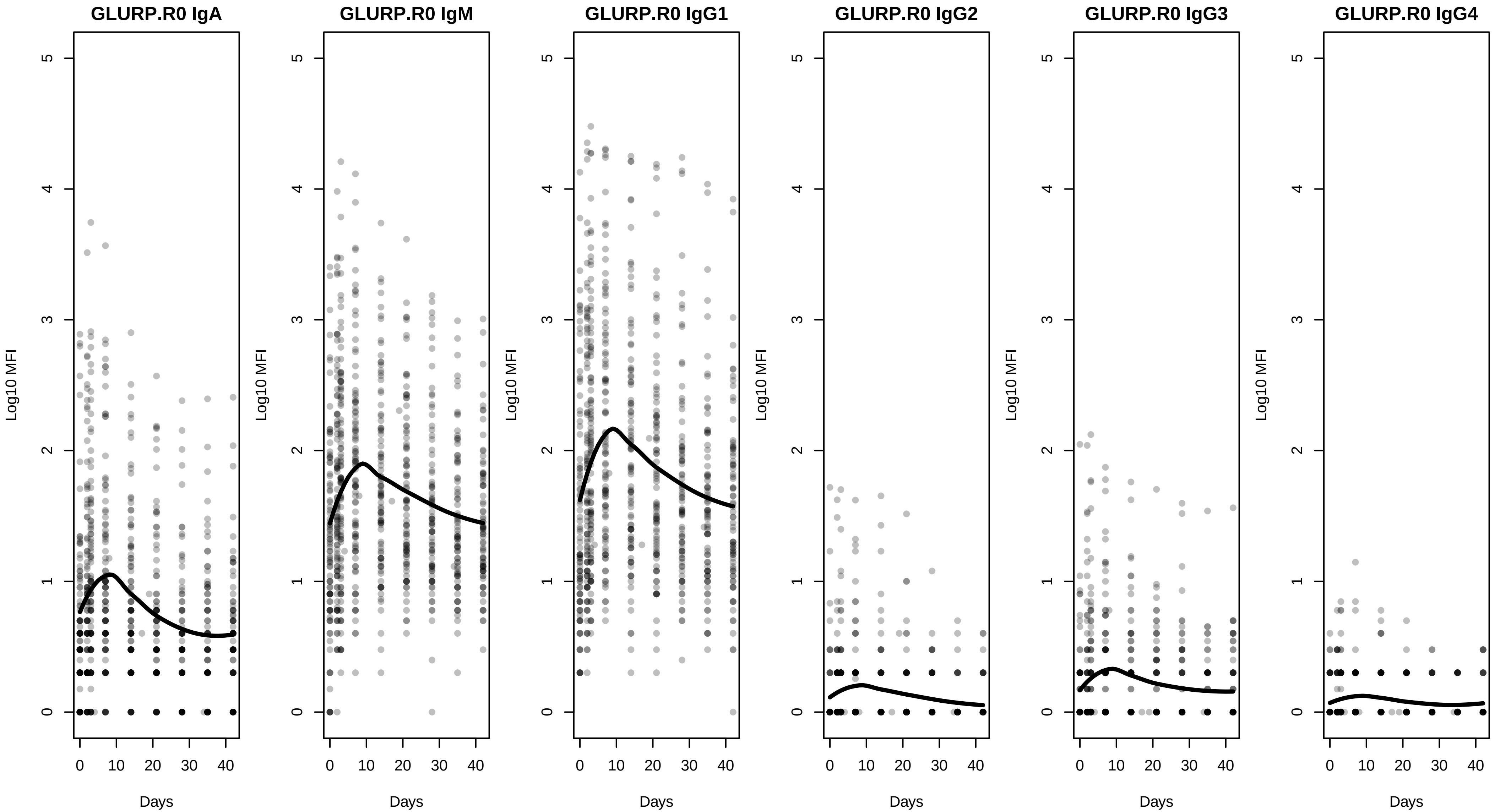

GLURP.R0 IgA
GLURP.R0 IgM
GLURP.R0 IgG1
GLURP.R0 IgG2
GLURP.R0 IgG3
GLURP.R0 IgG4
Log10 MFI
Days
0
1
2
3
4
5
0
10
20
30
40

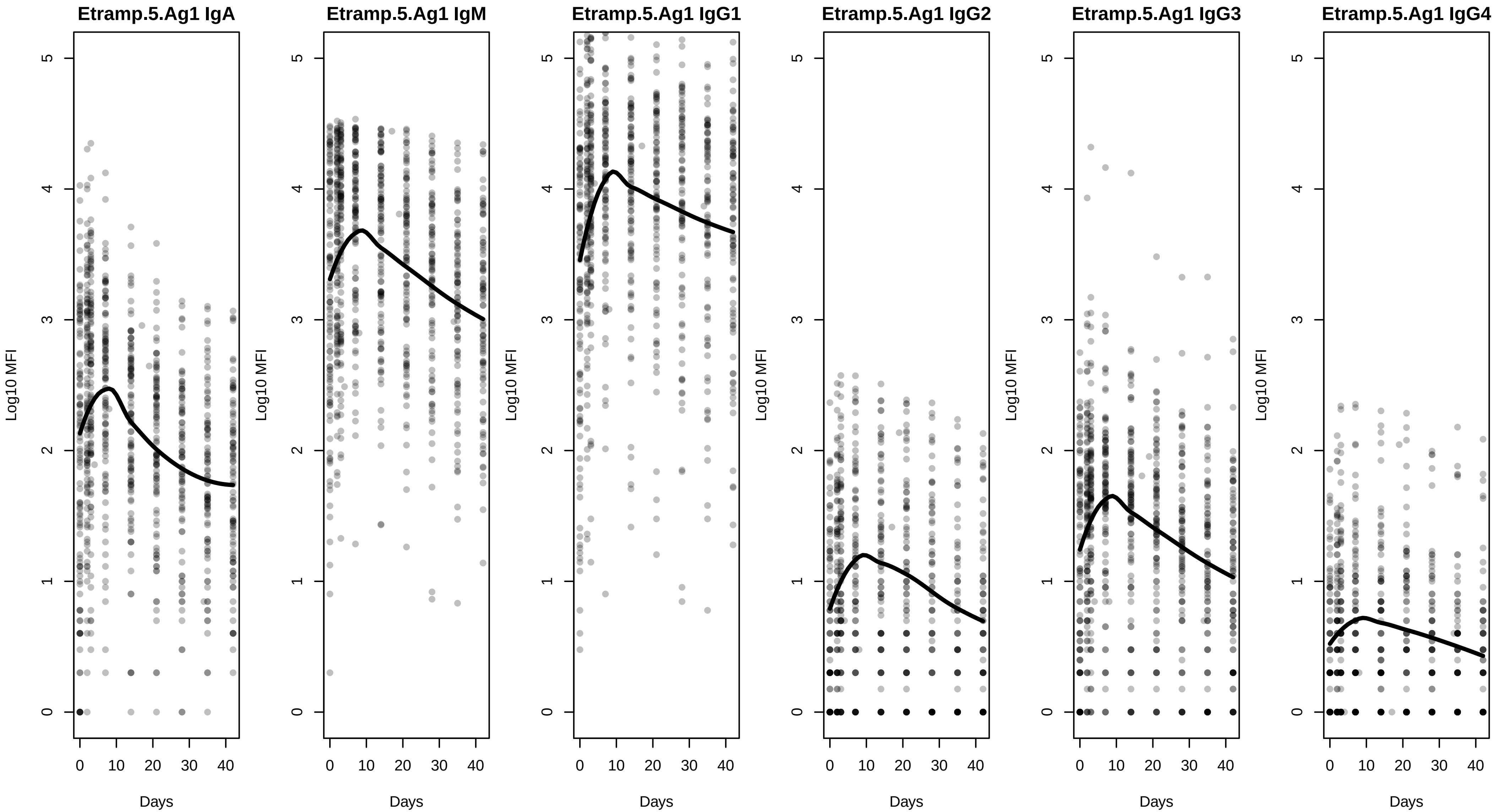

Etramp.5.Ag1 IgA
Etramp.5.Ag1 IgM
Etramp.5.Ag1 IgG1
Etramp.5.Ag1 IgG2
Etramp.5.Ag1 IgG3
Etramp.5.Ag1 IgG4
Log10 MFI
Days
0
1
2
3
4
5
0
10
20
30
40

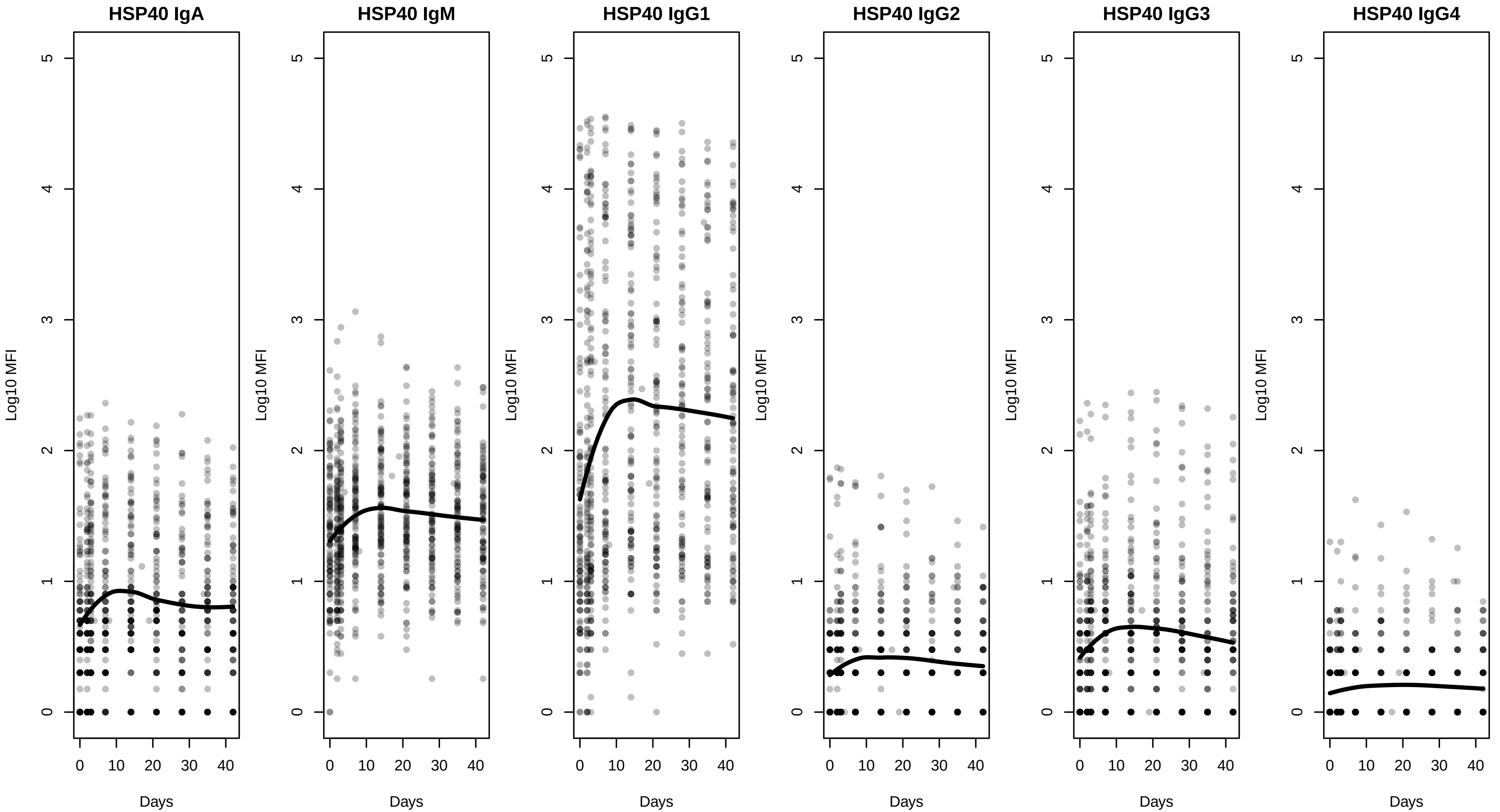

HSP40 IgA
HSP40 IgM
HSP40 IgG1
HSP40 IgG2
HSP40 IgG3
HSP40 IgG4
Log10 MFI
Days
0
1
2
3
4
5
0
10
20
30
40

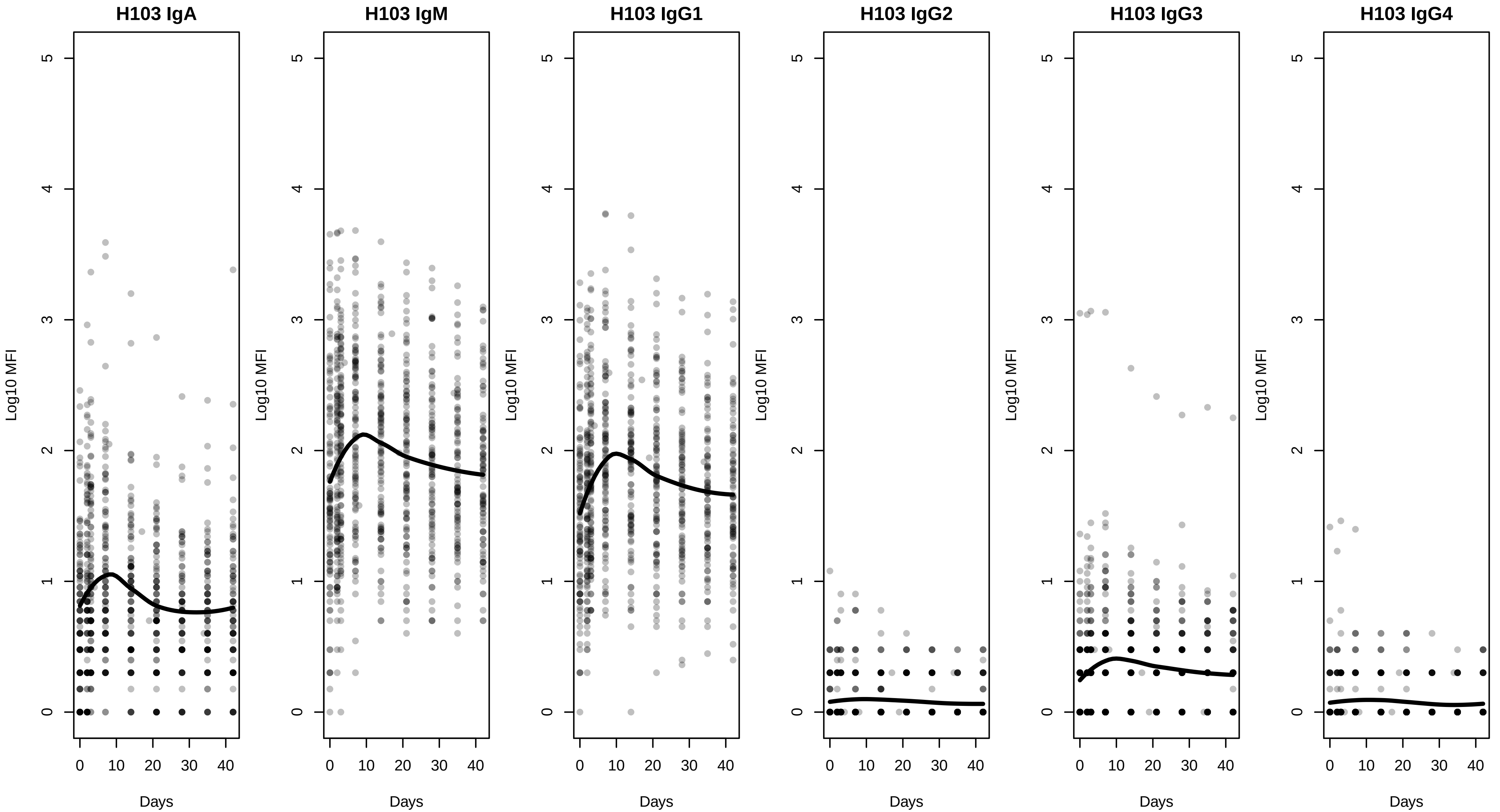
H103 IgA
H103 IgM
H103 IgG1
H103 IgG2
H103 IgG3
H103 IgG4
Log10 MFI
Days
0
1
2
3
4
5
0
10
20
30
40

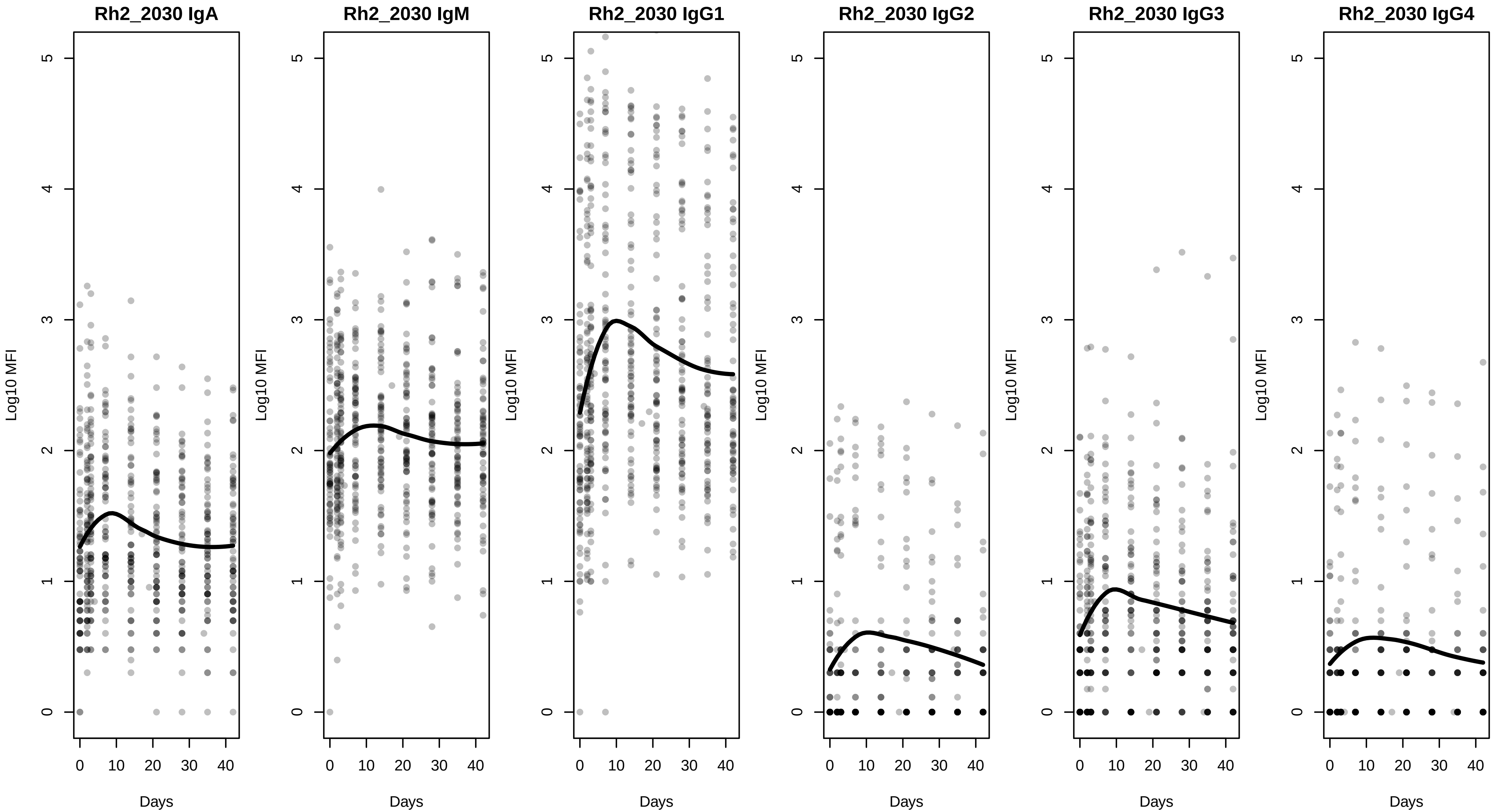

Rh2_2030 IgA
Rh2_2030 IgM
Rh2_2030 IgG1
Rh2_2030 IgG2
Rh2_2030 IgG3
Rh2_2030 IgG4
Log10 MFI
Days
0
1
2
3
4
5
0
10
20
30
40

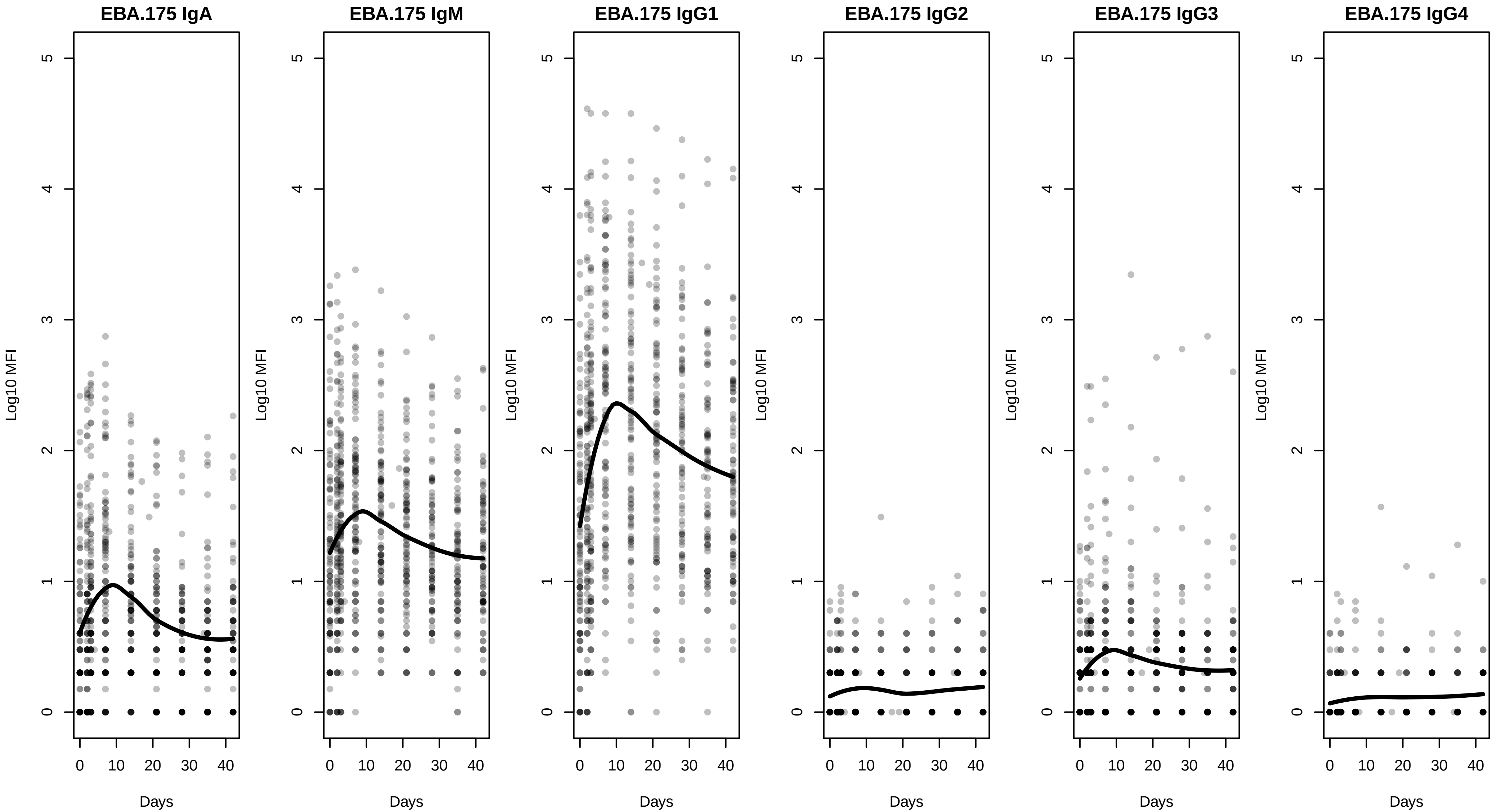

EBA.175 IgA
EBA.175 IgM
EBA.175 IgG1
EBA.175 IgG2
EBA.175 IgG3
EBA.175 IgG4
Log10 MFI
Days
0
1
2
3
4
5
0
10
20
30
40

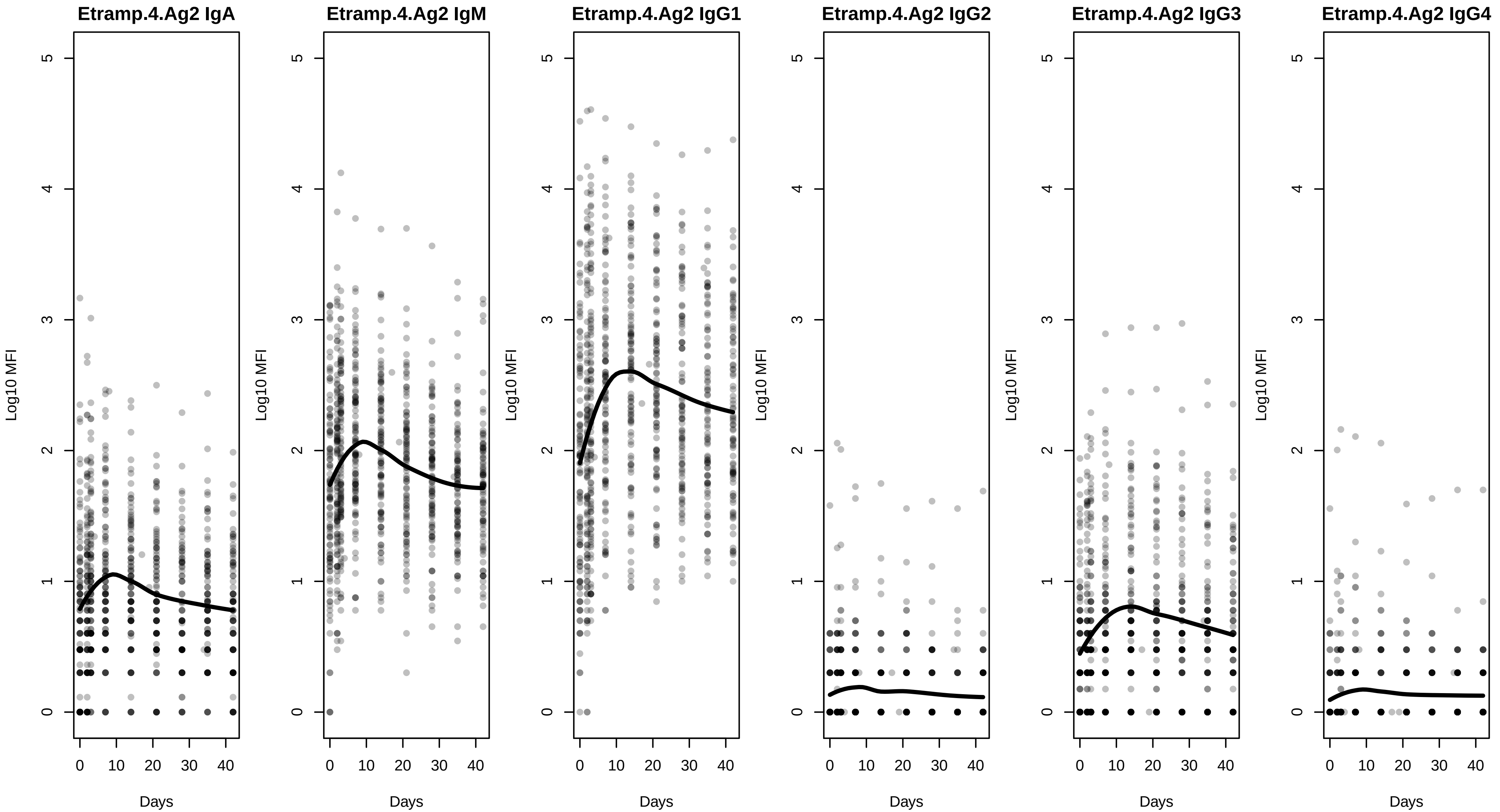
Etramp.4.Ag2 IgA
Etramp.4.Ag2 IgM
Etramp.4.Ag2 IgG1
Etramp.4.Ag2 IgG2
Etramp.4.Ag2 IgG3
Etramp.4.Ag2 IgG4
Log10 MFI
Days
0
1
2
3
4
5
0
10
20
30
40

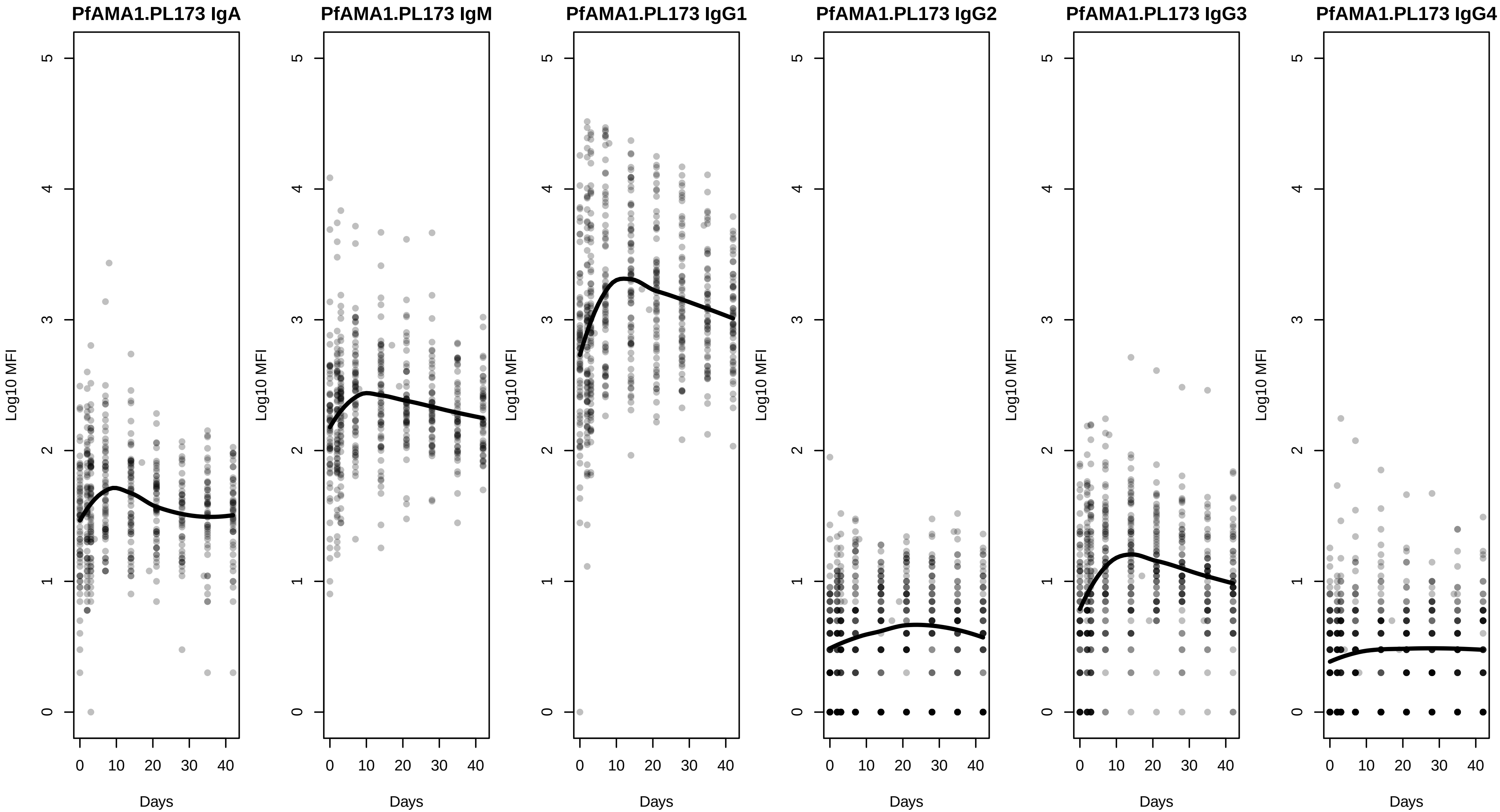
PfAMA1.PL173 IgA
PfAMA1.PL173 IgM
PfAMA1.PL173 IgG1
PfAMA1.PL173 IgG2
PfAMA1.PL173 IgG3
PfAMA1.PL173 IgG4
Log10 MFI
Days
0
1
2
3
4
5
0
10
20
30
40

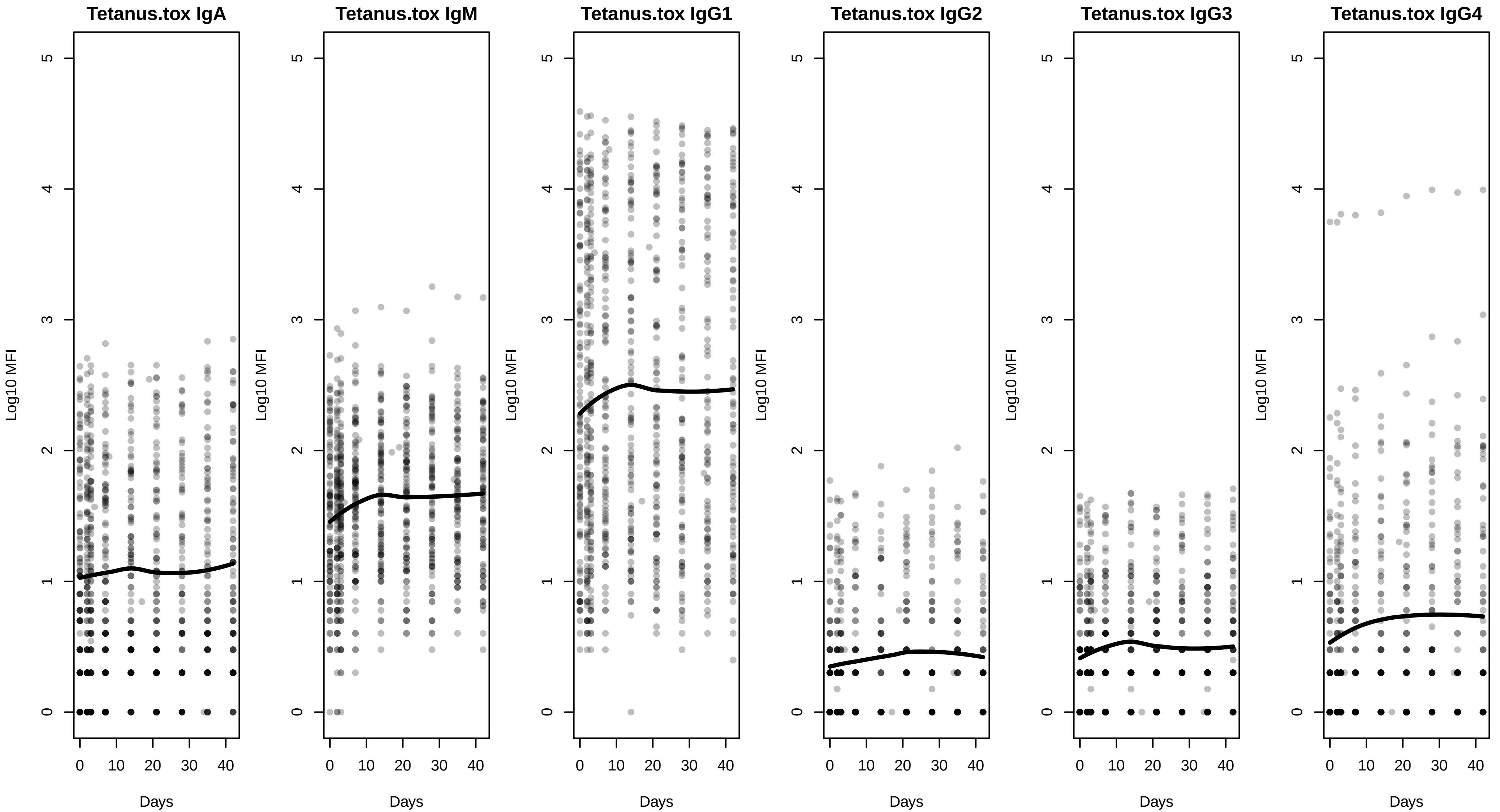

Tetanus.tox IgA
Tetanus.tox IgM
Tetanus.tox IgG1
Tetanus.tox IgG2
Tetanus.tox IgG3
Tetanus.tox IgG4
Log10 MFI
Days
0
1
2
3
4
5
0
10
20
30
40

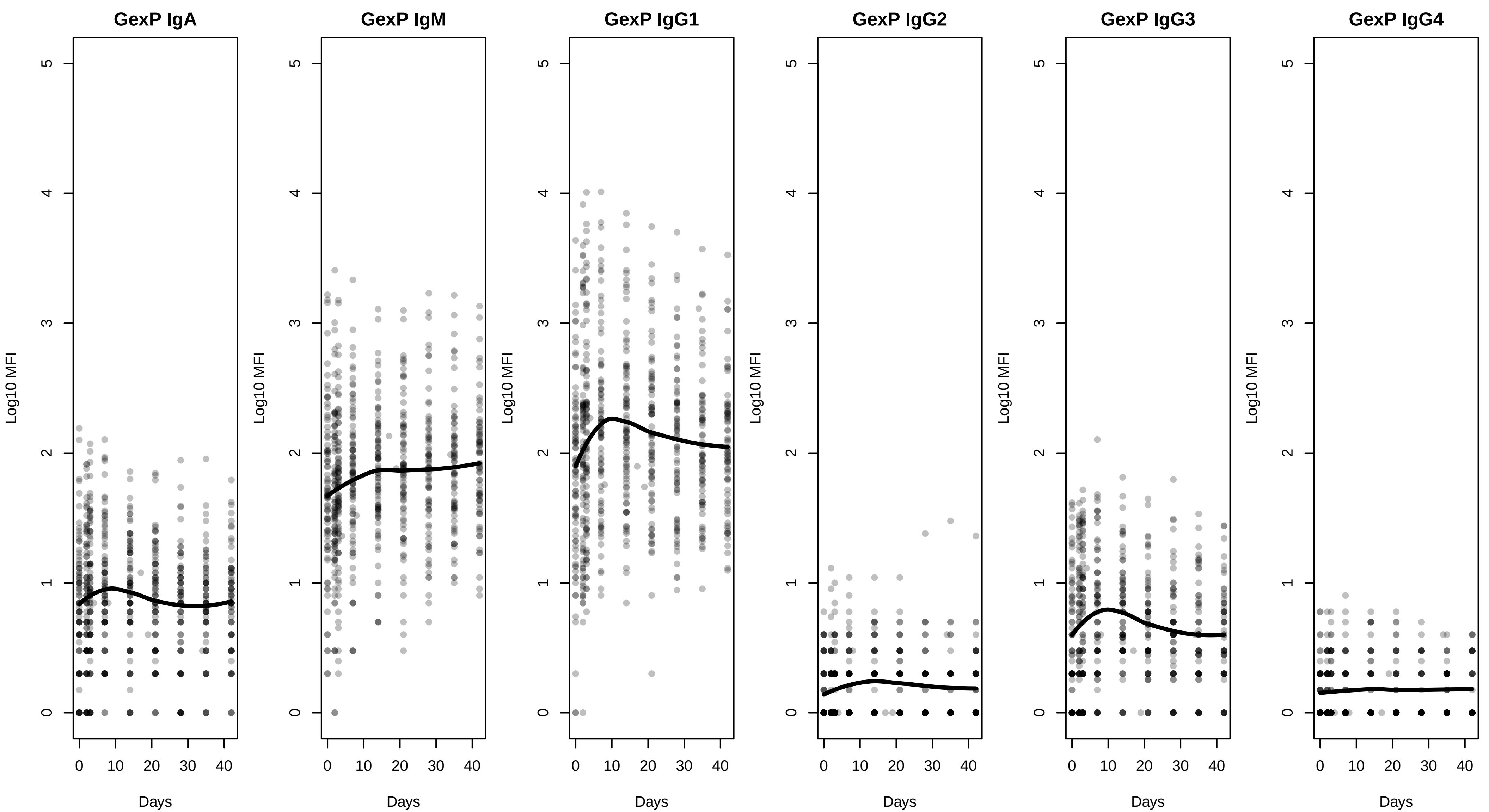

GexP IgA
GexP IgM
GexP IgG1
GexP IgG2
GexP IgG3
GexP IgG4
Log10 MFI
Days
0
1
2
3
4
5
0
10
20
30
40

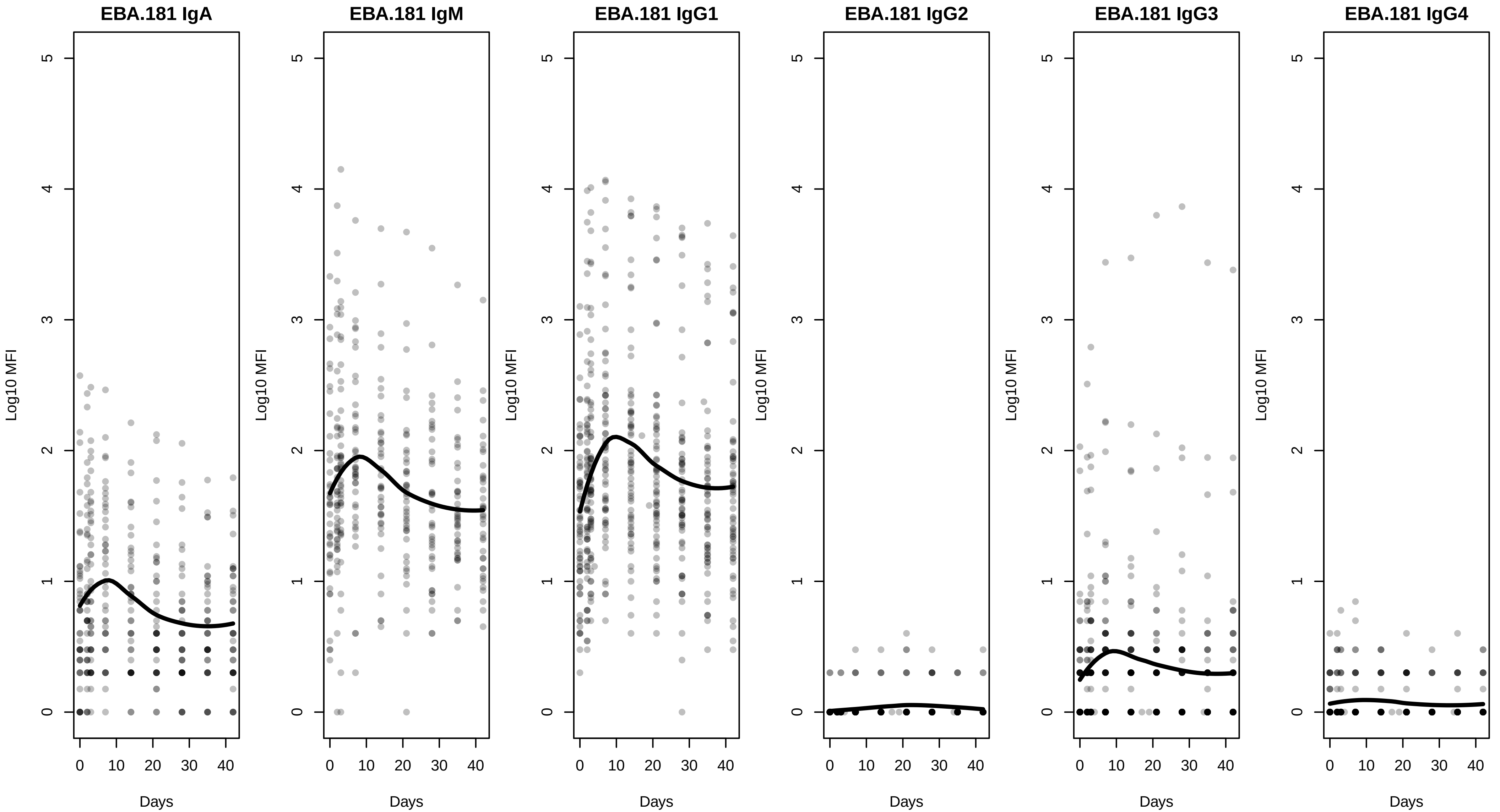
EBA.181 IgA
EBA.181 IgM
EBA.181 IgG1
EBA.181 IgG2
EBA.181 IgG3
EBA.181 IgG4
Log10 MFI
Days
0
1
2
3
4
5
0
10
20
30
40

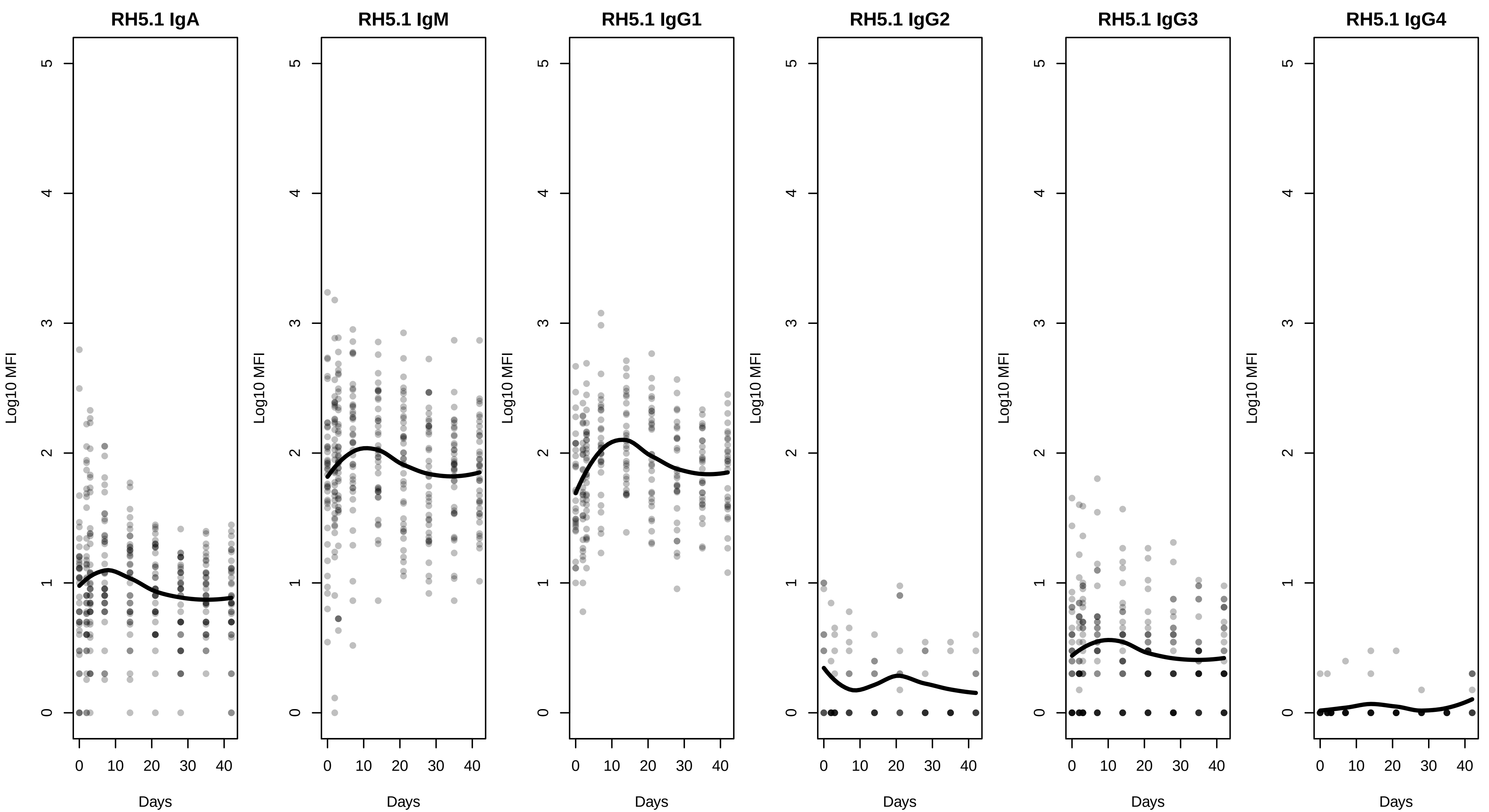

RH5.1 IgA
RH5.1 IgM
RH5.1 IgG1
RH5.1 IgG2
RH5.1 IgG3
RH5.1 IgG4
Log10 MFI
Days
0
1
2
3
4
5
0
10
20
30
40

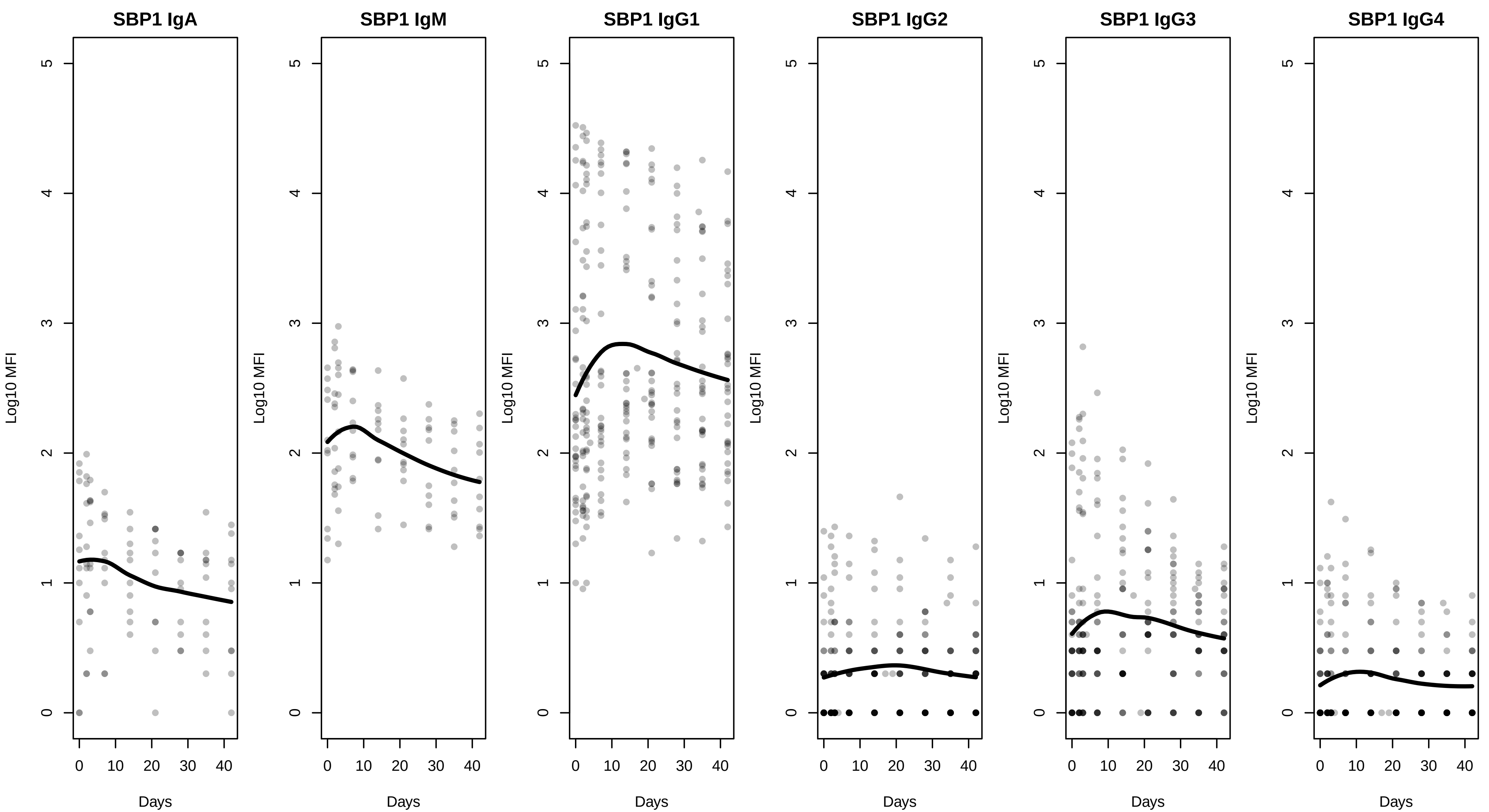
SBP1 IgA
SBP1 IgM
SBP1 IgG1
SBP1 IgG2
SBP1 IgG3
SBP1 IgG4
Log10 MFI
Days
0
1
2
3
4
5
0
10
20
30
40

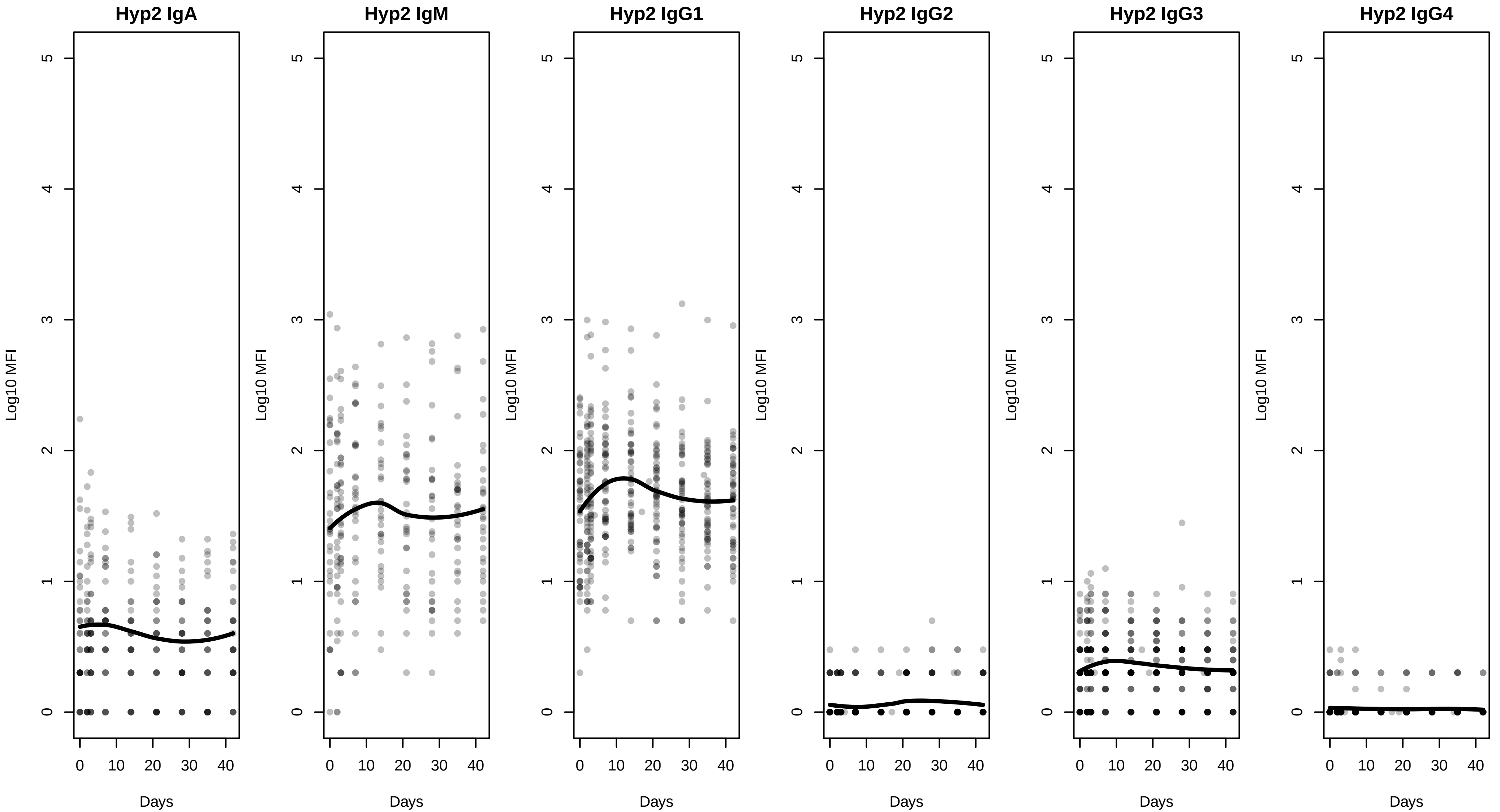

Hyp2 IgA
Hyp2 IgM
Hyp2 IgG1
Hyp2 IgG2
Hyp2 IgG3
Hyp2 IgG4
Log10 MFI
Days
0
1
2
3
4
5
0
10
20
30
40

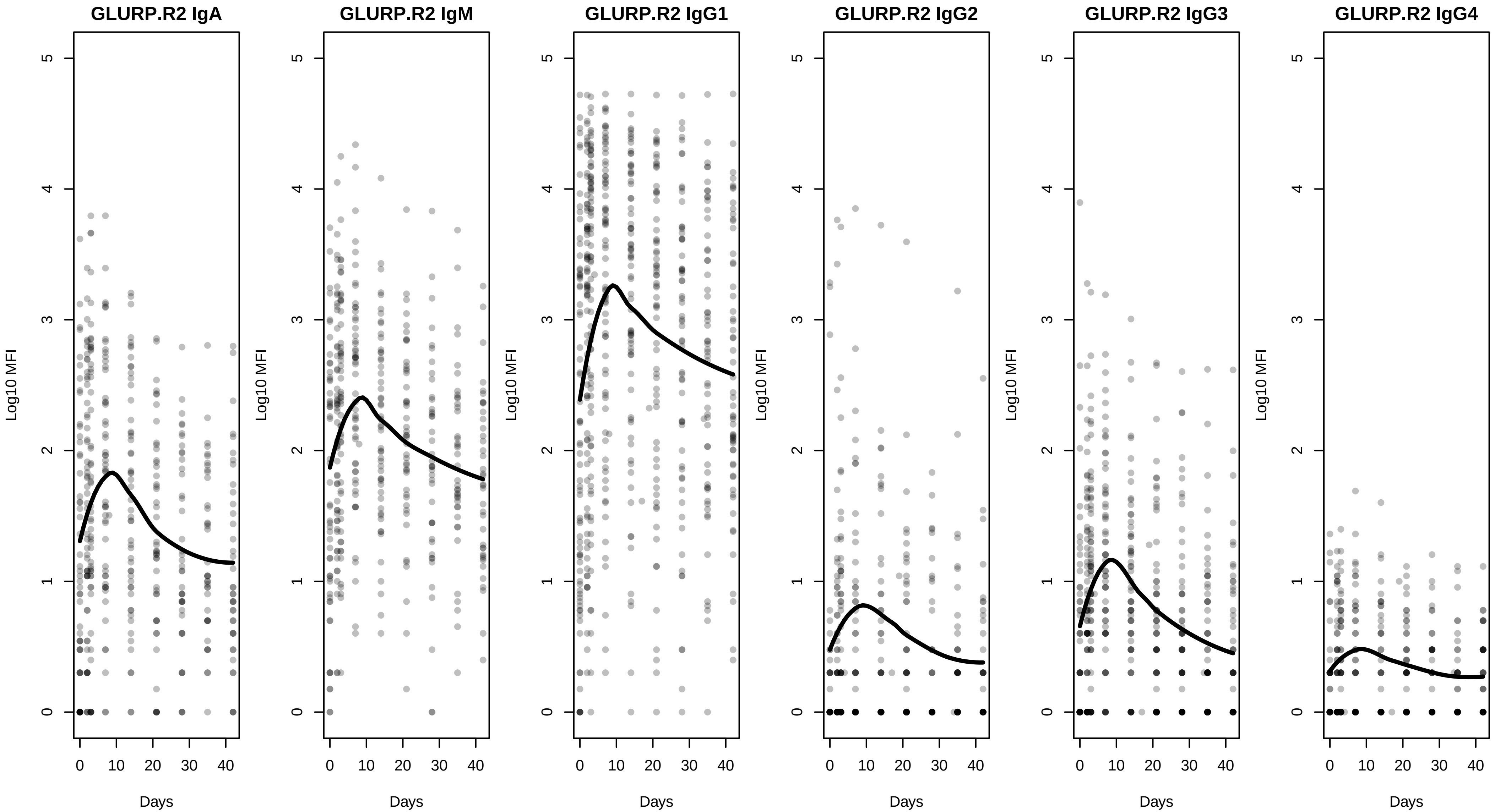

GLURP.R2 IgA
GLURP.R2 IgM
GLURP.R2 IgG1
GLURP.R2 IgG2
GLURP.R2 IgG3
GLURP.R2 IgG4
Log10 MFI
Days
0
1
2
3
4
5
0
10
20
30
40

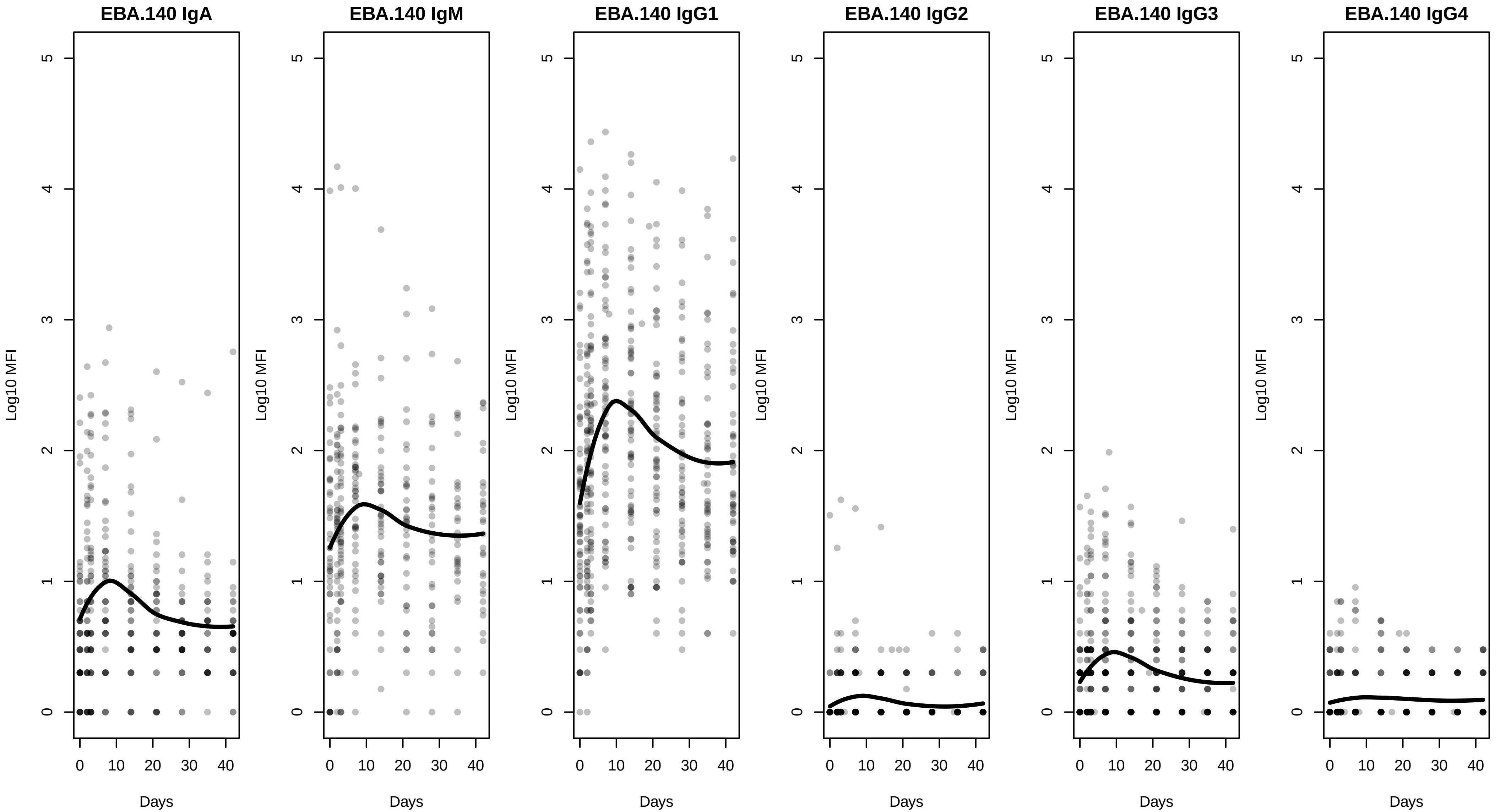

EBA.140 IgA
EBA.140 IgM
EBA.140 IgG1
EBA.140 IgG2
EBA.140 IgG3
EBA.140 IgG4
Log10 MFI
Days
0
1
2
3
4
5
0
10
20
30
40

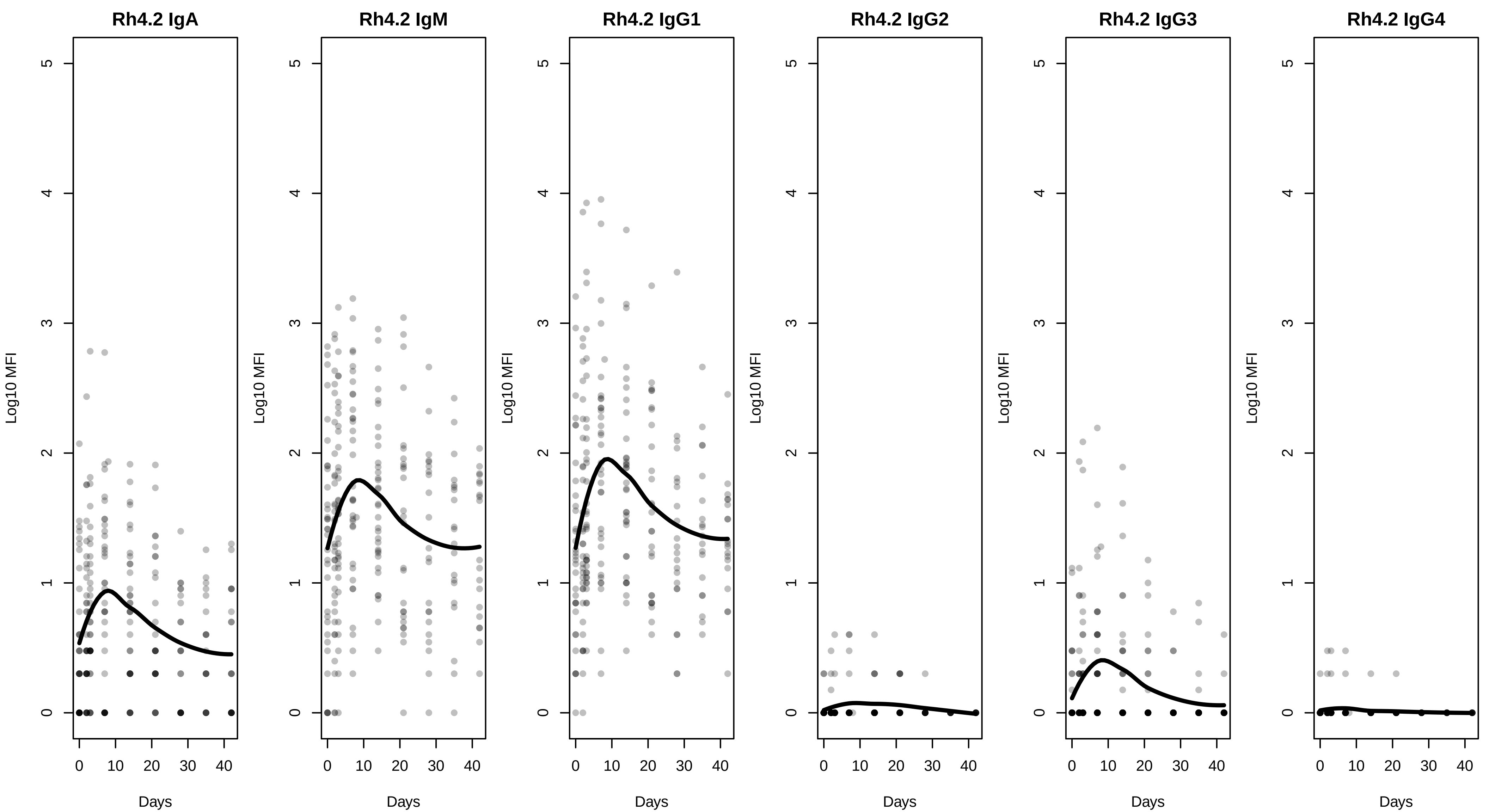

Rh4.2 IgA
Rh4.2 IgM
Rh4.2 IgG1
Rh4.2 IgG2
Rh4.2 IgG3
Rh4.2 IgG4
Log10 MFI
0
1
2
3
4
5
Days
0
10
20
30
40

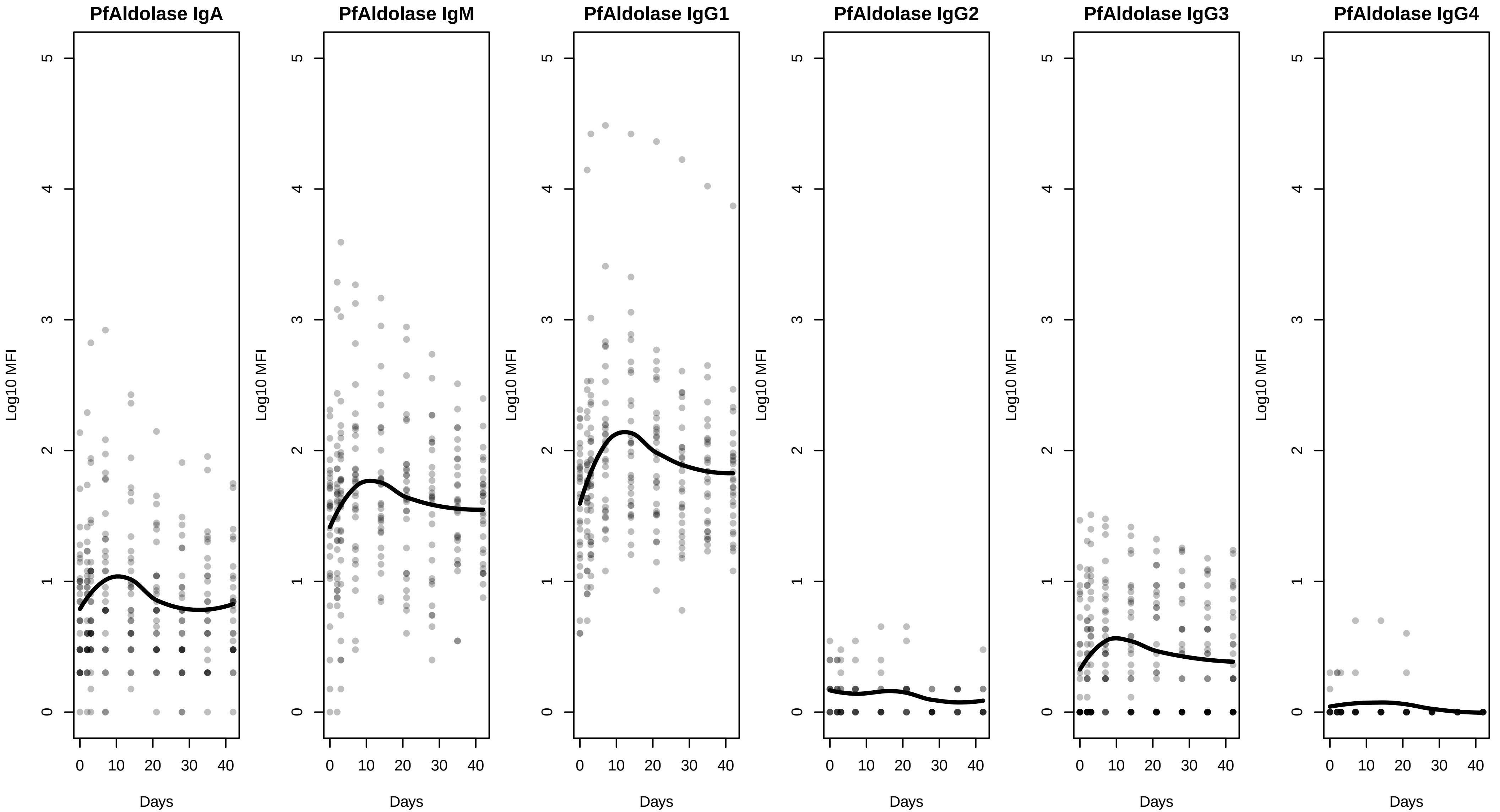
PfAldolase IgA
PfAldolase IgM
PfAldolase IgG1
PfAldolase IgG2
PfAldolase IgG3
PfAldolase IgG4
Log10 MFI
Days

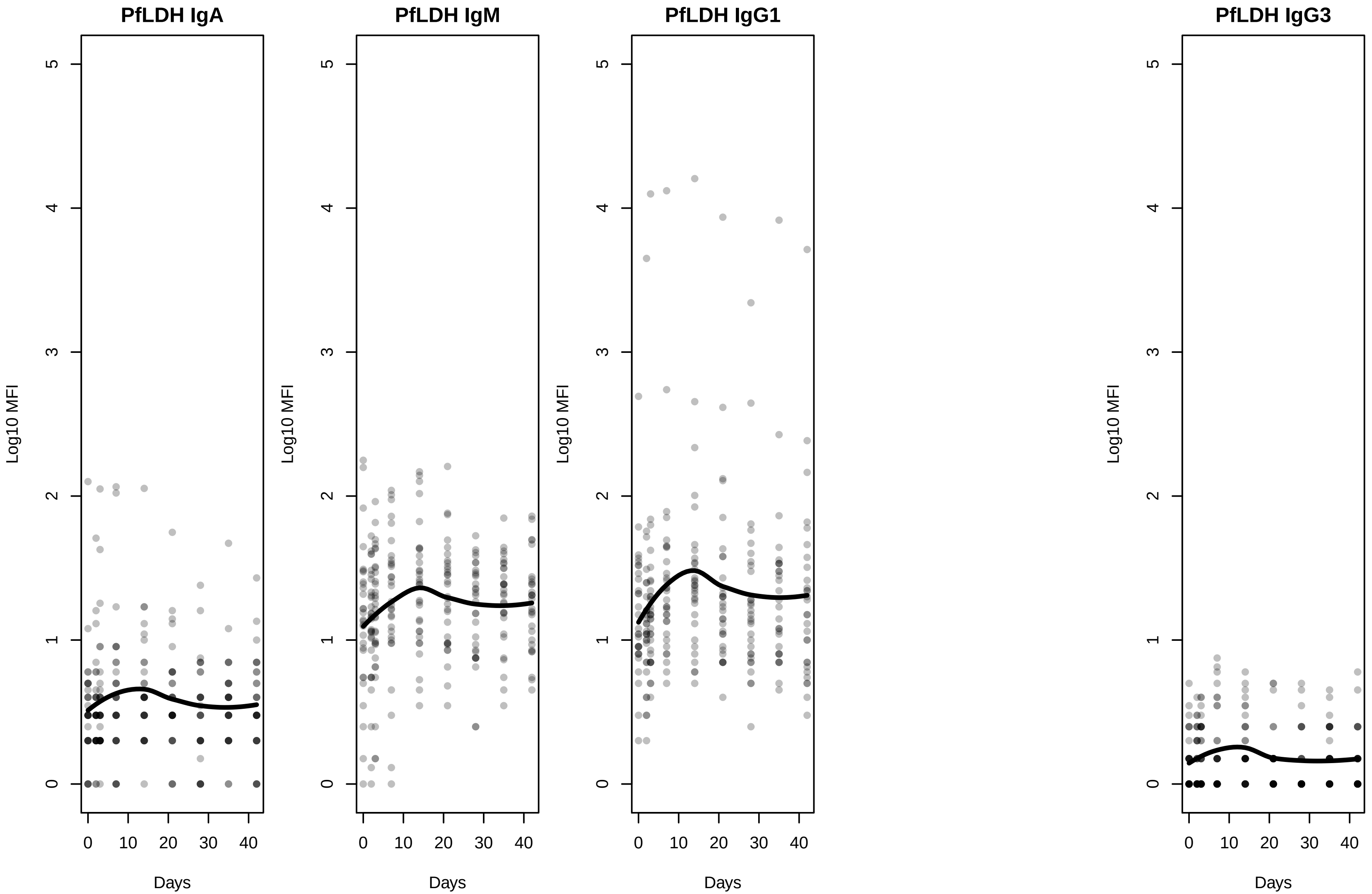

PfLDH IgA
PfLDH IgM
PfLDH IgG1
PfLDH IgG3
Log10 MFI
Days
0
1
2
3
4
5
0
10
20
30
40

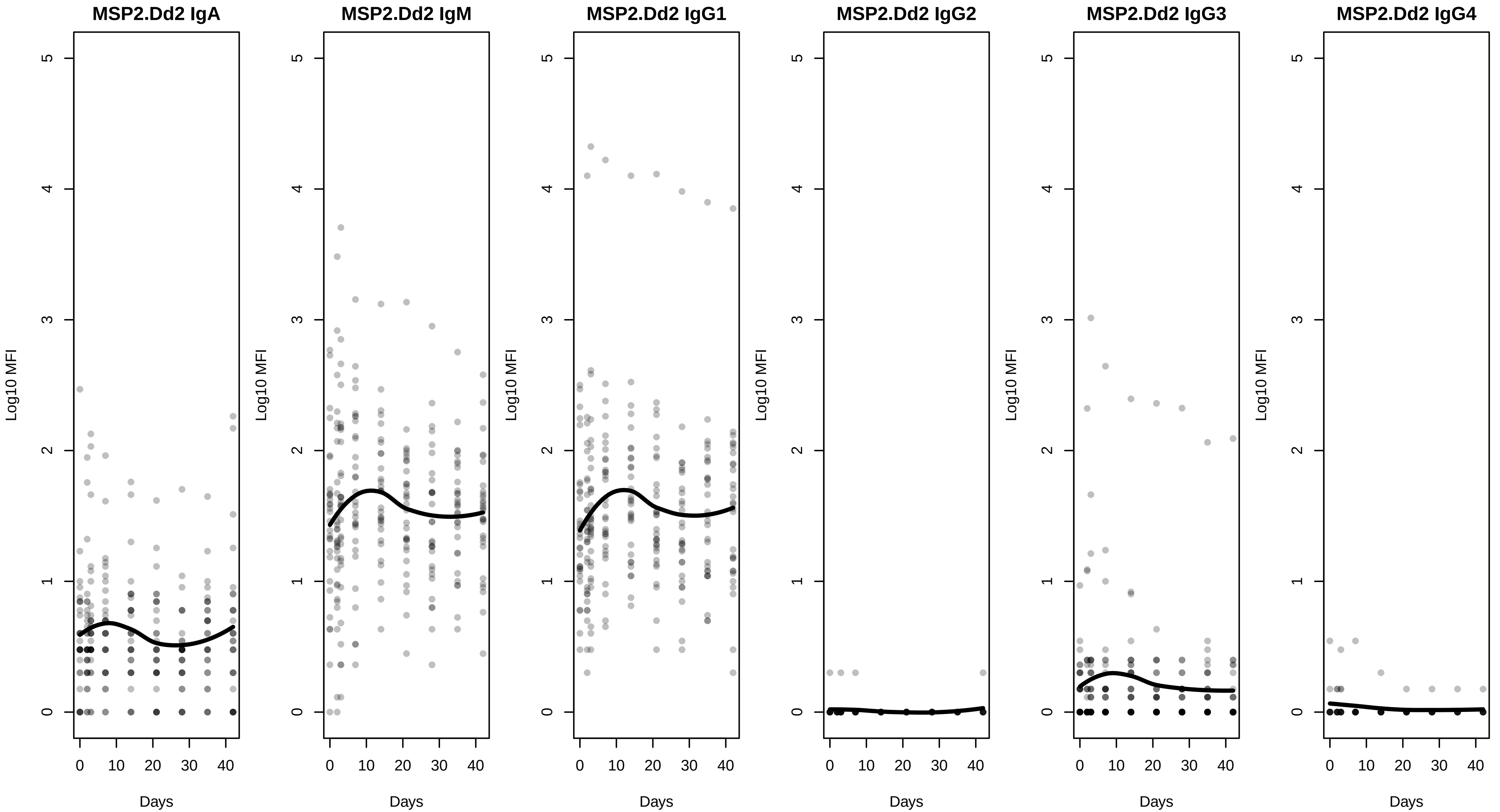
MSP2.Dd2 IgA
MSP2.Dd2 IgM
MSP2.Dd2 IgG1
MSP2.Dd2 IgG2
MSP2.Dd2 IgG3
MSP2.Dd2 IgG4
Log10 MFI
Days
0
1
2
3
4
5
0
10
20
30
40

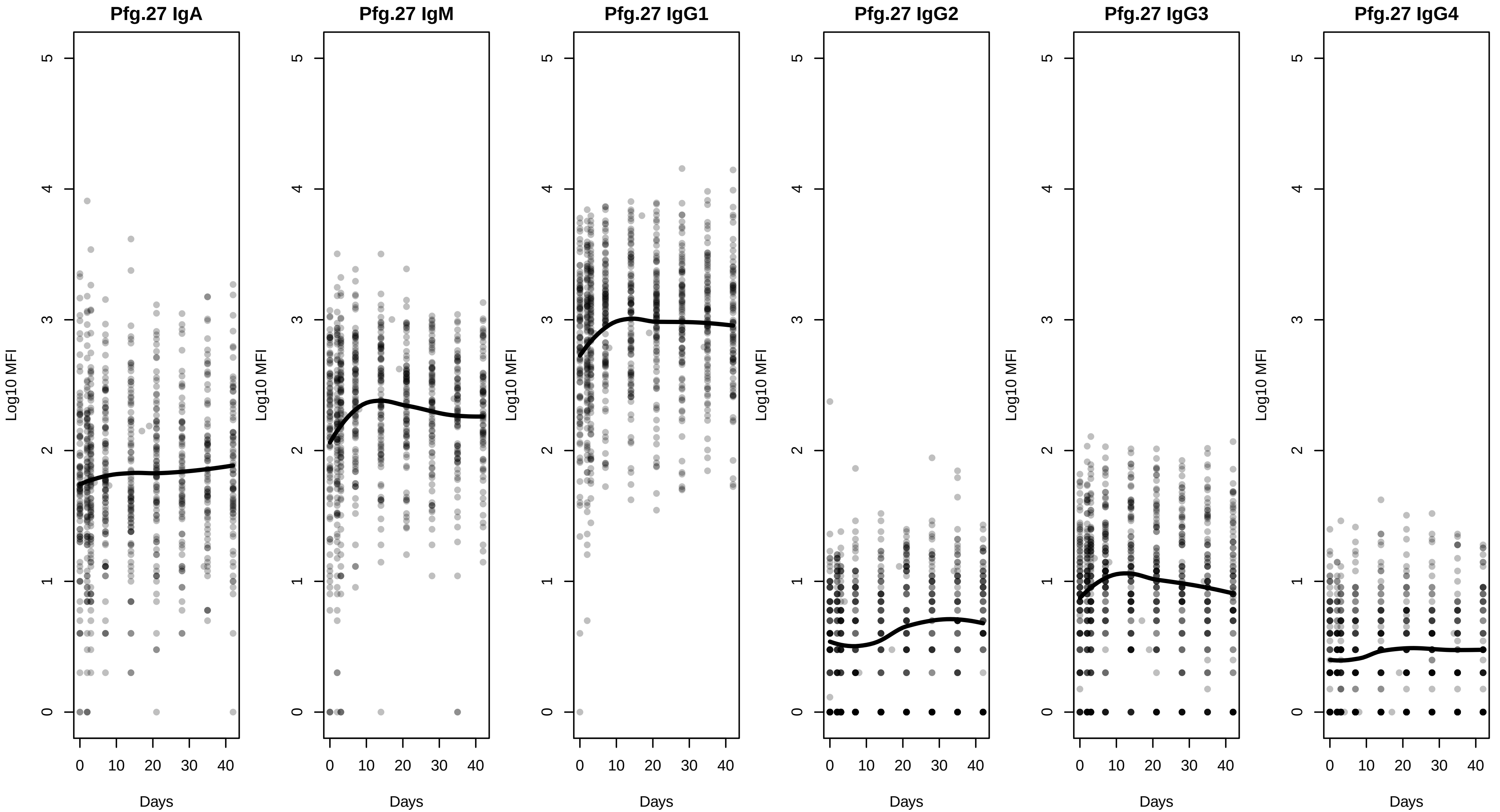
Pfg.27 IgA
Pfg.27 IgM
Pfg.27 IgG1
Pfg.27 IgG2
Pfg.27 IgG3
Pfg.27 IgG4
Log10 MFI
Days
0
1
2
3
4
5
0
10
20
30
40

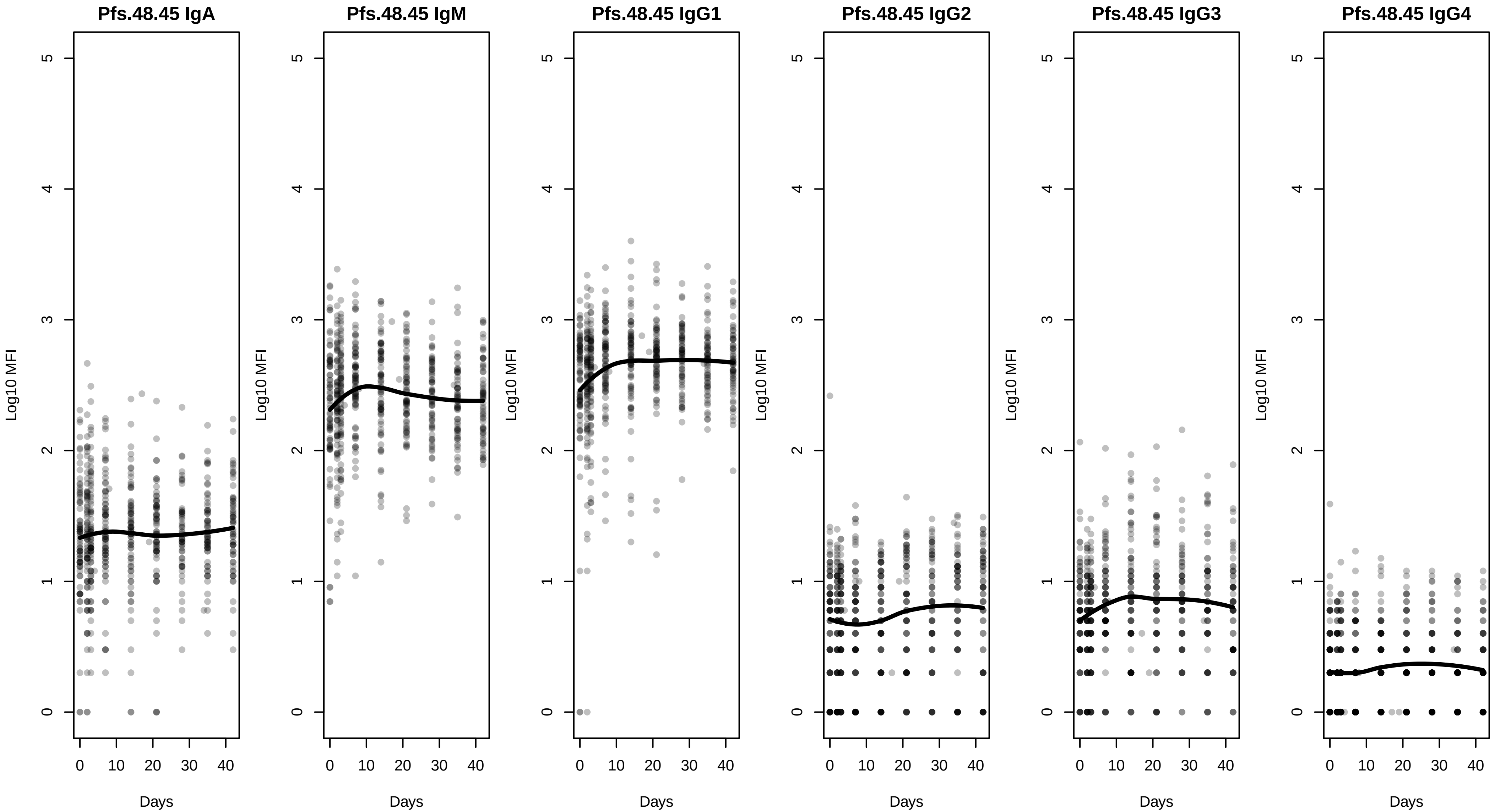

Pfs.48.45 IgA
Pfs.48.45 IgM
Pfs.48.45 IgG1
Pfs.48.45 IgG2
Pfs.48.45 IgG3
Pfs.48.45 IgG4
Log10 MFI
Days

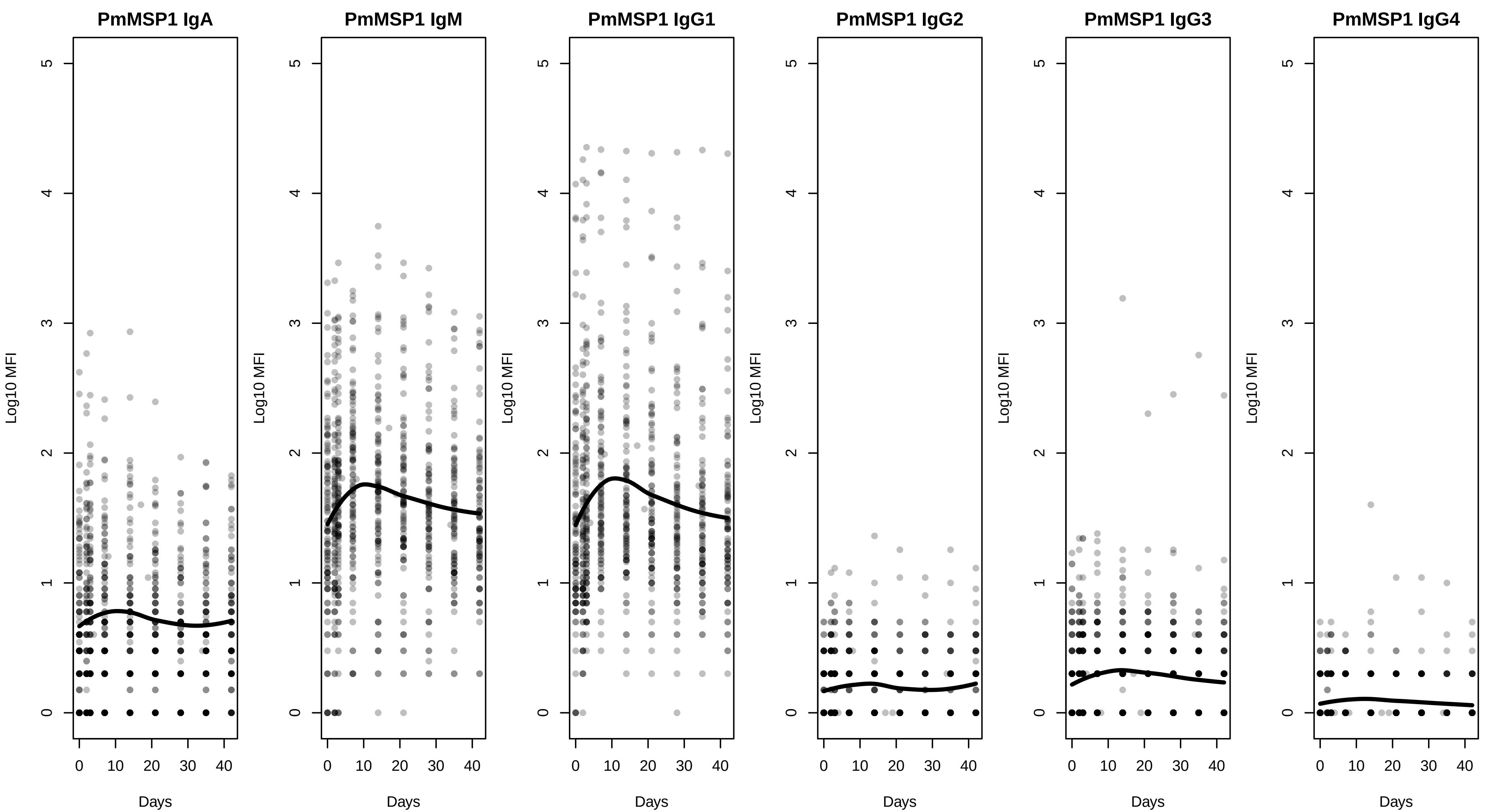

PmMSP1 IgA
PmMSP1 IgM
PmMSP1 IgG1
PmMSP1 IgG2
PmMSP1 IgG3
PmMSP1 IgG4
Log10 MFI
Days
0
1
2
3
4
5
10
20
30
40

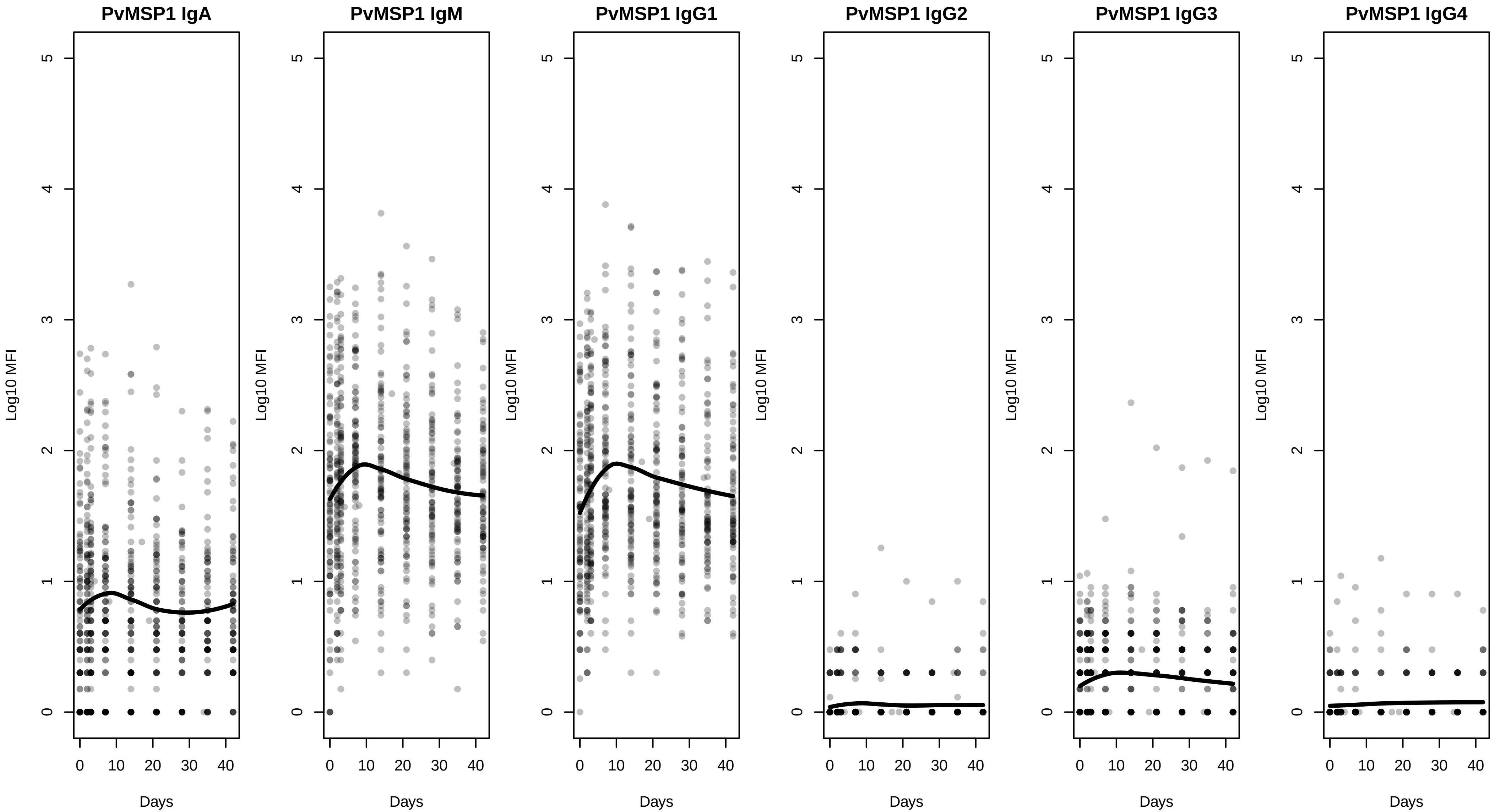
PvMSP1 IgA
PvMSP1 IgM
PvMSP1 IgG1
PvMSP1 IgG2
PvMSP1 IgG3
PvMSP1 IgG4
Log10 MFI
Days
0
1
2
3
4
5
0
10
20
30
40

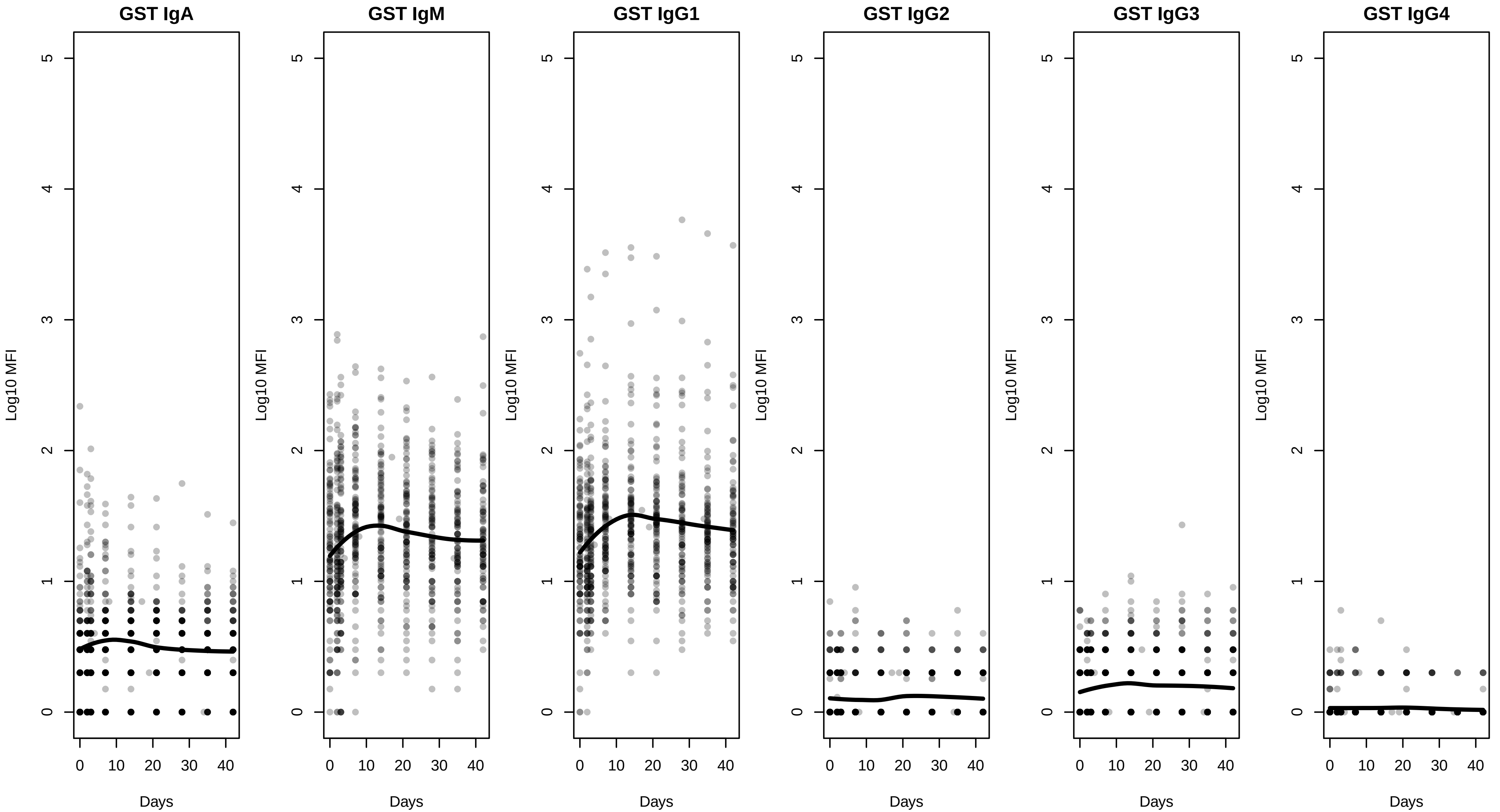
GST IgA
GST IgM
GST IgG1
GST IgG2
GST IgG3
GST IgG4
Log10 MFI
Days
0
1
2
3
4
5
0
10
20
30
40
